# SLC7A11, a Potential Therapeutic Target Through Induced Ferroptosis in Colon Adenocarcinoma

**DOI:** 10.3389/fmolb.2022.889688

**Published:** 2022-04-20

**Authors:** Xin Cheng, Yadong Wang, Liangchao Liu, Chenggang Lv, Can Liu, Jingyun Xu

**Affiliations:** ^1^ General Surgery Department, Wuhu Hospital of Traditional Chinese Medicine, Wuhu, China; ^2^ The First Affiliated Hospital of Wannan Medical College, Wuhu, China; ^3^ School of Basic Medicine, Wannan Medical College, Wuhu, China

**Keywords:** SLC7A11, ferroptosis, immune infiltrate, immune microenvironment, COAD

## Abstract

**Background:** Ferroptosis induced by SLC7A11 has an important translational value in the treatment of cancers. However, the mechanism of SLC7A11 in the pathogenesis of colon adenocarcinoma (COAD) is rarely studied in detail.

**Methods:** SLC7A11 expression was explored with The Cancer Genome Atlas (TCGA), Gene Expression Omnibus (GEO) databases, and Western blot assay. The correlation of SLC7A11 expression with the abundance of infiltrating immune cells was evaluated *via* the TIMER database. The relation of SLC7A11 expression with immune cell markers was investigated *via* Gene Expression Profiling Interactive Analysis (GEPIA). The co-expression genes of SLC7A11 were screened by R packages, and the PPI was constructed *via* the STRING database. SLC7A11 and co-expressed gene modulators were selected by NetworkAnalyst and DSigDB database. The correlations between SLC7A11 and cancer immune characteristics were analyzed *via* the TIMER and TISIDB databases.

**Results:** SLC7A11 is overexpressed in most tumors, including COAD. The expression level of SLC7A11 has a significant correlation with the infiltration levels of CD8^+^ T cells, neutrophils, and dendritic cells in COAD. The infiltrated lymphocyte markers of Th1 cell such as TBX21, IL12RB2, IL27RA, STAT1, and IFN-γ were strongly correlated with SLC7A11 expression. Five hub genes co-expressed with SLC7A11 that induce ferroptosis were identified, and mir-335-5p, RELA, and securinine have regulatory effects on it. SLC7A11 was negatively correlated with the expression of chemokines and chemokine receptors, such as CCL17, CCL19, CCL22, CCL23, CXCL14, CCR10, CX3CR1, and CXCR3, in COAD.

**Conclusion:** SLC7A11 may play a role in induced ferroptosis and regulating tumor immunity, which can be considered as potential therapeutic targets in COAD.

## Introduction

Colon adenocarcinoma (COAD) is one of the most common malignant tumors of the digestive system; the diagnosis rate and the mortality rate represent about 1/10 of the total cancer cases (El Kinany et al., 2020). In recent years, the incidence of COAD is increasing annually, with the highest incidence in China (Roslan et al., 2019). Traditional treatments for COAD include radiation, chemotherapy, and surgery. Currently, new therapeutic methods such as bio-targeted therapy, precision therapy, and immunotherapy have been gradually applied in the treatment of colon cancer (Sandhu et al., 2019; Bao et al., 2020). However, drug resistance and immune escape restrict the therapeutic effect of these therapies (Fidelle et al., 2020). Therefore, finding new therapeutic targets to halt or slow disease progression is an urgent priority.

Studies showed that ferroptosis as a novel-induced programmed cell death, which can effectively inhibit tumor growth by inducing ferroptosis of tumor cells (Mou et al., 2019), and it also plays a critical role in reversing cisplatin resistance of tumors (Guo et al., 2018). Therefore, modulating ferroptosis may have important transforming significance in various ferroptosis-associated diseases. Glutathione peroxidase 4 (GPX4) was shown to be a central regulator of ferroptosis; glutathione (GSH) is a necessary cofactor for the biological activity of GPX4; GSH synthesis depends on the Gys/Glu reverse transfer system (Xc-); System Xc- consists of two subunits, including solute carrier family 7, membrane 11 (SLC7A11), and heavy chain subunit SLC3A2; SLC7A11 is responsible for the primary activity of System Xc-, while SLC3A2 plays an auxiliary coordinating role (Xie et al., 2016; Koppula et al., 2018; Song et al., 2018). Latest studies have shown that 2-imino-6-methoxy-2H-chromene-3-carbothioamide (IMCA) downregulates SLC7A11 expression through the AMPK/mTOR pathway and induces ferroptosis (Zhang et al., 2020). However, whether SLC7A11 can be an effective target for COAD treatment needs more theoretical support.

In this study, bioinformatics methods were used to analyze SLC7A11 expression and clinical information in COAD patients. Then, we used the TIMER and GEPIA databases to study the relationship between SLC7A11 expression and infiltrating immune cells and their corresponding gene marker sets. In addition, the protein–protein interaction network of SLC7A11 was explored by STRING. The results showed that the overexpression of SLC7A11 inhibited the ferroptosis of tumor cells and promoted the development and metastasis of COAD. Targeted inhibition of SLC7A11 may be a promising therapeutic strategy for inducing ferroptosis or in combination with immunotherapy for COAD.

## Materials and Methods

### Clinical Samples and Ethics

Six patients undergoing radical resection of colon cancer were collected in the First Affiliated Hospital of Wannan Medical College and pathologically diagnosed with colon cancer. None of them received chemotherapy or immunotherapy before surgery. Tumor tissues and paracancerous tissues were collected from the six patients (the adjacent normal tissues were extracted >3 cm from the tumor margin). This study was approved by the Ethics Committee of Wannan Medical College (NO. 2021081).

### Data Source

The Cancer Genome Atlas (TCGA) (https://genomecancer.ucsc.edu/), a free data portal of largescale cancer genome project. The data of COAD patients with the expression of RNA-Seq and matching clinical information (include pathologic stage, histologic grade, OS time, gender, and age) were obtained by the TCGA tools.

### Retrieval of Datasets

In order to compare the expression level of SLC7A11 in cancer and normal tissues, two datasets were involved in this study, GSE21510 and GSE24514, where both were from the GEO database (https://www.ncbi.nlm.nih.gov/geo/). In GSE21510, the expression profiles of cancer cells in 104 patients with colorectal cancer were examined by laser microdissection and oligonucleotide microarray analysis (Tsukamoto et al., 2011). GSE24514 illustrated microsatellite instability in colorectal cancer patients (Alhopuro et al., 2012).

### Western Blot (WB)

Total proteins were extracted from the cancer and paracancerous tissues, after protein concentration was detected, WB assay was performed. The primary antibodies used were as follows: SLC7A11 antibody (Abcam, ab175186) (diluted at 1:5,000) and GAPDH recombinant antibody (proteintech, 80570-1-RR) (diluted at 1:5,000) at room temperature (RT) for 1 h. After washing three times with TBST, 5 min/wash, the membrane was incubated with HRP-labeled goat antirabbit IgG (Beyotime, A0208) (diluted at 1:1,000) as the secondary antibody for 1 h at RT. Finally, the signal was detected by chemiluminescence, and the bands’ gray value was measured using ImageJ software.

### Correlation Analysis Between SLC7A11 Expression and Immune Cell Infiltration

The correlation of SLC7A11 expression with the abundance of infiltrating immune cells, including tumor-associated macrophage (TAM), neutrophils, macrophages, dendritic cells, B cells, CD8^+^ T cells, CD4^+^ T cells, and their subtypes in COAD patients was evaluated *via* the TIMER database (http://cistrome.org/TIMER/). The relationship between the expression of the SLC7A11 gene and tumor purity was also displayed.

### Correlation Analysis of SLC7A11 Expression and Immune Cell Markers

The relation of SLC7A11 expression with multiple markers for immune cells was investigated *via* The Gene Expression Profiling Interactive Analysis (GEPIA) (http://gepia.cancer-pku.cn/index.html). Moreover, we used TIMER data to validate the genes which were of significant correlation with SLC7A11 expression in the GEPIA web.

### Co-Expression Network Establishment of SLC7A11 and GO–KEGG Analysis

The co-expression genes of SLC7A11 were screened by R packages (limma). The correlation between the SLC7A11 expression level and co-expression genes were examined by Pearson correlation coefficients and the Z-test from the TCGA databases (related parameter settings: |Pearson correlation coefficient| > 0.5 and *p*-value <0.001). The STRING database (v11.5, https://www.string-db.org/) was used to construct the protein–protein interactions (PPI). In addition, GO–KEGG enrichment analysis was performed by Bioconductor package ‘‘clusterProfiler.”

### Construction of the TF-miRNA Co-Regulatory Network

The NetworkAnalyst database (https://www.networkanalyst.ca/) was used to identify TF-miRNA co-regulate with co-expression genes. The common network topology measures were also computed based on well-established igraph R package. TF and gene target data were derived from ENCODE ChIP-seq data. Only the peak intensity signal <500 and the predicted regulatory potential score <1 were used.

### Screening of Potential Therapeutic Drugs

The Enrichr platform was used for drug screening of SLC7A11 and co-expression genes. The access of the DSigDB database was acquired using Enrichr (https://amp.pharm.mssm.edu/Enrichr/). Potential therapeutic agents were determined by the adj. *p* values and the abundance of acting on SLC7A11 and co-expression genes.

### TISIDB Database Analysis

To study the association between SLC7A11 and chemokine/chemokine receptor expression, we evaluated the expression levels of chemokine/chemokine receptors of tumor-infiltrating immune cells through the “chemokine” module of the TISIDB database (http://cis.hku.hk/TISIDB/). To further clarify the immune correlation of SLC7A11 in cancer, the “immunomodulator” module was used to analyze and evaluate the correlation between SLC7A11 expression and the levels of immune checkpoint genes.

### Enzyme-Linked Immunosorbent Assay (ELISA)

The levels of CCL17, CXCL14, and CXCR3 in cancer and paracancerous tissues were measured by ELISA kits from Yuanju, Inc. (Shanghai, China) (Yuanju, YJ779969/YJ714549/YJ719414) according to the manufacturer’s protocol.

## Results

### Patient Characteristics

In total, the RNA-sequencing data and detailed clinical prognostic information resources of 478 COAD samples were obtained from the TCGA database. The clinical information including tumor extent (T), lymph node invasion (N), detectable metastasis (M), histologic grade, age at diagnosis, overall survival (OS), and lymphatic invasion are summarized in Table 1.

**TABLE 1:** Clinical characteristics of the COAD patients.

Characteristic	Levels	Overall
N		478
Tumor extent (T) stage, n (%)	T1	11 (2.3%)
	T2	83 (17.4%)
	T3	323 (67.7%)
	T4	60 (12.6%)
Lymph node invasion (N) stage, n (%)	N0	284 (59.4%)
	N1	108 (22.6%)
	N2	86 (18%)
Detectable metastasis (M) stage, n (%)	M0	349 (84.1%)
	M1	66 (15.9%)
Pathologic stage, n (%)	Stage I	81 (17.3%)
	Stage II	187 (40%)
	Stage III	133 (28.5%)
	Stage IV	66 (14.1%)
Age, n (%)	<=65	194 (40.6%)
	>65	284 (59.4%)
Lymphatic invasion, n (%)	NO	266 (61.3%)
	YES	168 (38.7%)
Overall survival (OS) event, n (%)	Alive	375 (78.5%)
	Dead	103 (21.5%)
Gender, n (%)	Female	226 (47.3%)
	Male	252 (52.7%)
Age, median (IQR)		69 (58, 77)

**Note:** A small amount of samples may be lost during the statistics of different clinical variables in the TCGA database. T1, the carcinoma invaded the submucosa but not involve the lamina propria; T2, the carcinoma invaded the lamina propria; T3, the carcinoma penetrated the lamina propria; T4, the carcinoma invaded the visceral peritoneum or adhered to adjacent organs; N0, no regional lymph node metastasis; N1, limited to 1–3 regional lymph node metastases, or no regional lymph node metastasis, but any number of tumor nodules; N2, four or more regional lymph nodes metastases; M0, imaging showed no distant metastasis; M1, metastases to one or more distant sites, organs, or peritoneum; Stage I, the carcinoma confined within the intestinal wall; Stage II, the carcinoma penetrated the intestinal wall and invaded the mucosa or serosa without lymph node metastasis; Stage III, local lymph node metastasis; and Stage IV, invasion of adjacent organs or distant metastasis.

### Higher SLC7A11 Expression in Tumor Samples Than That in Normal Tissues

**FIGURE 1:**
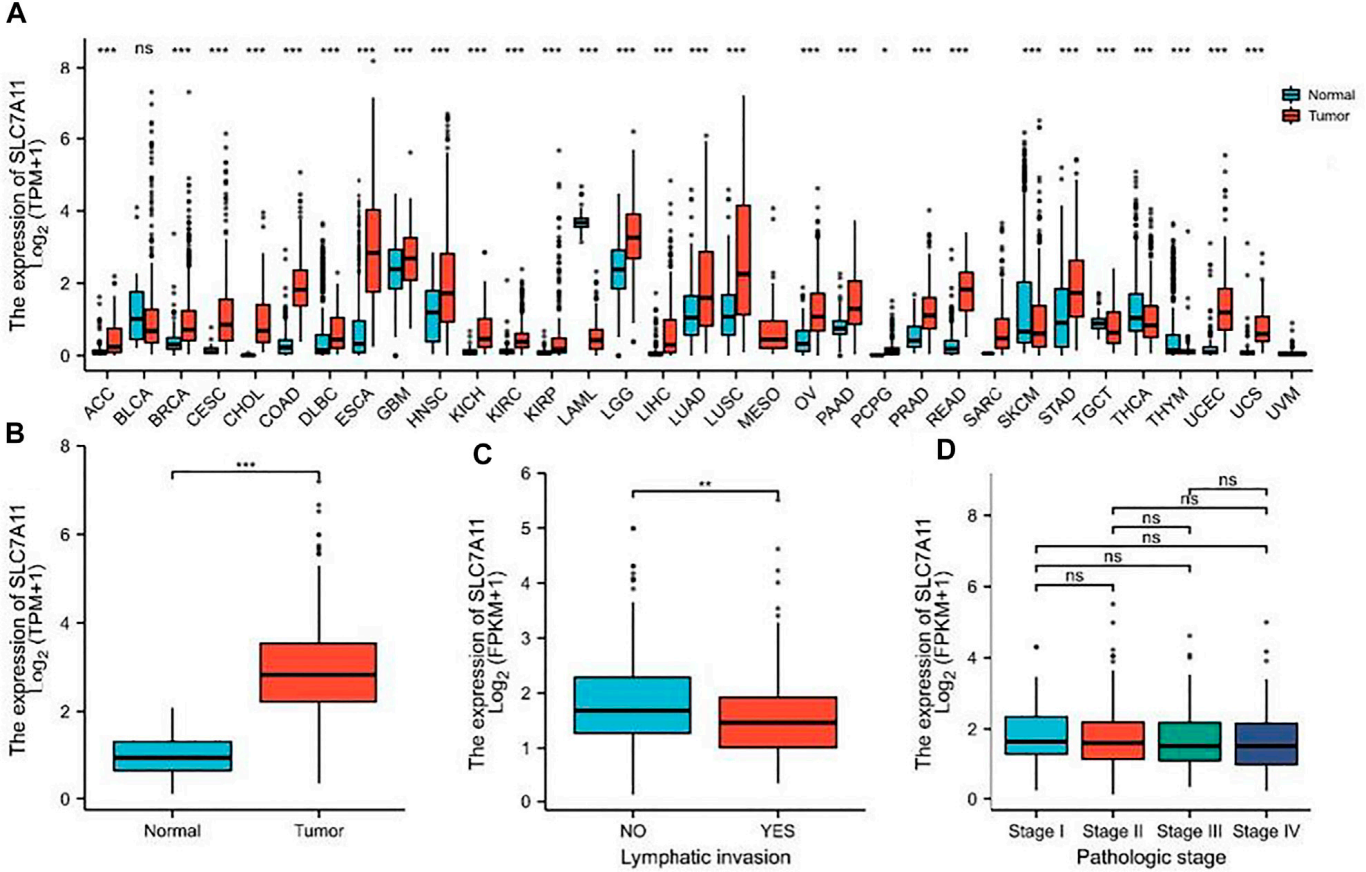
SLC7A11 expression status in pan-cancer. **(A)** Expression of human SLC7A11 in distinct cancers compared with normal tissues. **(B)** Expression of SLC7A11 in COAD compared with normal tissues, n (normal) = 41 and n (tumor) = 480. **(C)** Comparison of SLC7A11 expression levels in lymphatic invasion, n (NO) = 266 and n (YES) = 168. **(D)** There was no significant difference in the SLC7A11 mRNA level among different pathological stages, n (Stage I, II, III, IV) = 81, 187, 133, 66, respectively. *p < 0.05. **p < 0.01. ***p < 0.001. ns *p* > 0.05.

The mRNA expression level of SLC7A11 was analyzed in various cancer types (Figure 1A). The gene expression level of SLC7A11 was significantly higher in tumor samples than that in normal tissues of COAD in TCGA and GEO databases (*p* < 0.01, Figure 1B). The expression of SLC7A11 is downregulated in COAD tissue when lymphatic invasion occurs (*p* < 0.01, Figure 1C). However, SLC7A11 expression is relatively stable in all pathologic stages of COAD (*p* > 0.05, Figure 1D).

**FIGURE 2:**
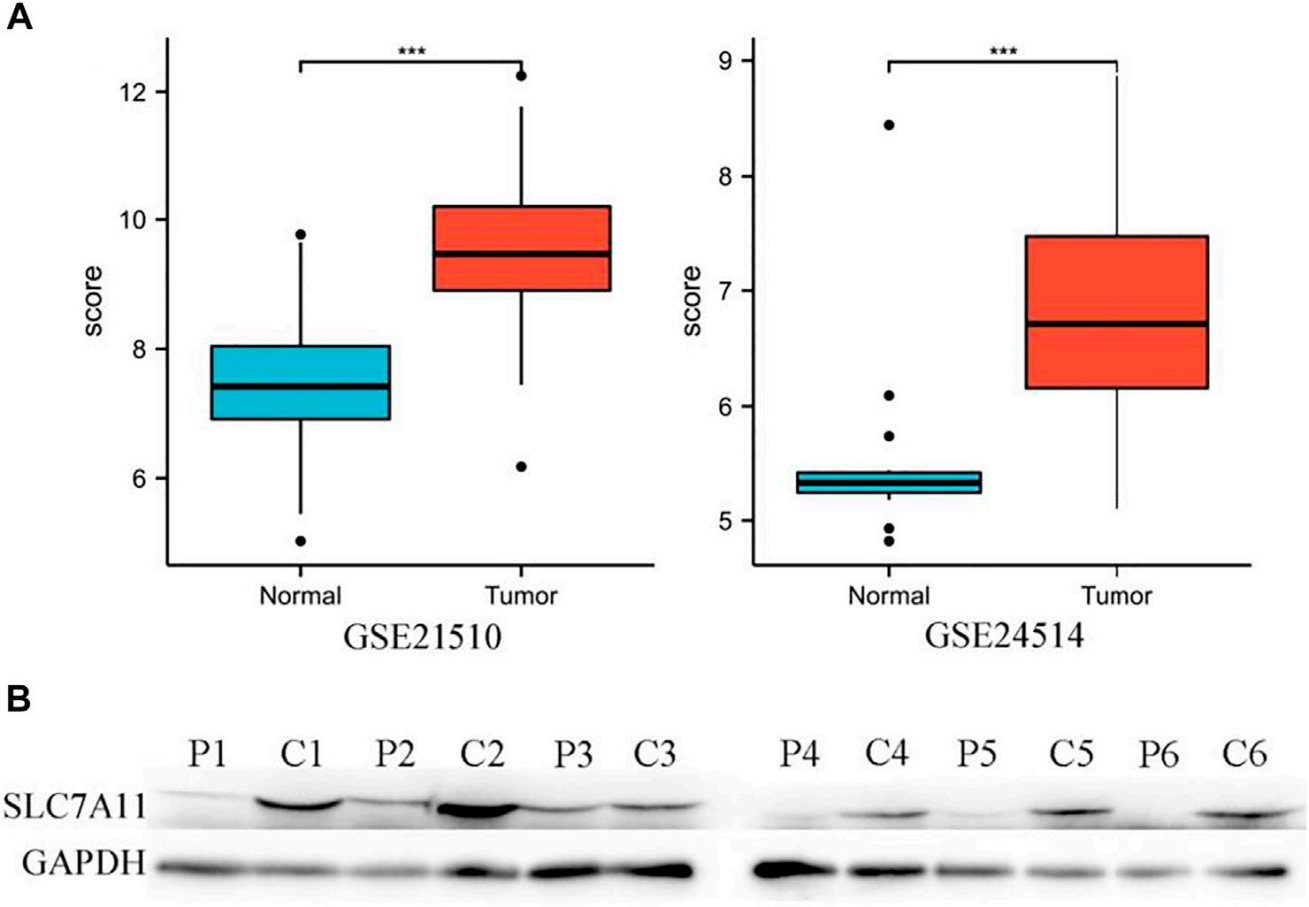
High expression of SLC7A11 in colon cancer. **(A)** Analysis of SLC7A11 gene expression in GEO datasets, n (normal) = 25 and n (tumor) = 123 in GSE21510, n (normal) = 15 and n (tumor) = 34 in GSE24514. ***p < 0.001. **(B)** Expression levels of SLC7A11 were detected by Western blot analysis, P1-6: paracancerous tissues of patients 1–6, C1-6: cancer tissues of patients 1–6, the bands gray value of C1-6 significantly higher than the P1-6 (*p* < 0.01). Correlation analysis between SLC7A11 expression and infiltrating immune cells.

In addition, the high expression of SLC7A11 in COAD was also observed in two datasets of the GEO database (Figure 2A), and the aforementioned results were also verified by WB (Figure 2B).

**FIGURE 3:**
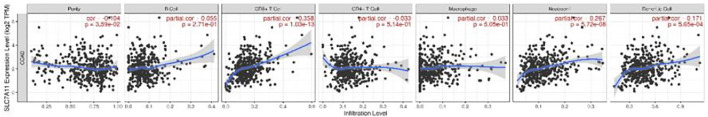
Correlation of SLC7A11 expression with immune cell-infiltrated in COAD.

The expression level of SLC7A11 had obviously positive correlation with infiltrating levels of CD8^+^ T cells (*r* = 0.358, *p* = 1.03e-13), neutrophils (*r* = 0.267, *p* = 5.72e-08), and dendritic cells (*r* = 0.171, *p* = 5.65e-04) in COAD, but it was negatively correlated with tumor purity (*r* = -0.104, *p* = 3.59e-02) (Figure 3).

To intensely explore the possible role of SLC7A11 in the infiltration of various immune cells in COAD, the GEPIA and TIMER databases were used to execute the relationships between SLC7A11 and several immune marker sets. Furthermore, various functional T cells including Th1, Th2, Th17, and Treg were also been examined in this study. Results showed that the levels of most immune sets marking different T cells, TAMs, macrophages, monocytes, and DCs were associated with the SLC7A11 expression in COAD (Table 2).

**TABLE 2:** Correlation analysis between SLC7A11 and immune cell markers in TIMER and GEPIA.

Cell Type	Gene Symbol	None		Purity		Tumor		Normal	
		Core	P	Core	P	R	P	R	P
Tumor associated	MS4A4A	0.08	0.087	−0.379	**	0.06	0.32	0.15	0.34
Macrophage (TAM)	CCL2	0.127	**	−0.354	**	0.003	0.96	0.14	0.38
	CCR5	0.091	0.052	−0.436	**	0.11	0.058	0.38	0.14
	CD80	0.103	*	−0.313	**	0.028	0.64	0.41	**
	CD86	0.131	**	−0.42	**	0.081	0.15	0.2	0.22
Monocyte	CD14	0.003	0.954	−0.394	**	0.07	0.25	0.13	0.43
	CD16	−0.027	0.567	−0.222	**	0.11	0.065	0.2	0.22
	CD115	−0.034	0.468	−0.366	**	0.022	0.72	0.18	0.25
Neutrophil	CD11b	−0.098	*	−0.25	**	0.013	0.83	0.25	0.11
	CD15	0.219	**	0.019	0.699	*	0.86	0.37	*
	CD66b	−0.259	**	0.103	*	−0.045	0.46	0.27	0.09
NK	CD7	0.013	0.783	−0.472	**	0.11	0.061	-0.084	0.6
	XCL1	−0.021	0.66	−0.241	**	0.019	0.76	-0.14	0.39
	KIR3DL1	0.113	*	−0.249	**	0.36	**	0.099	0.54
Dendritic cell	CD1C	−0.073	0.121	−0.331	**	−0.076	0.21	0.085	0.6
	CD11c	0.061	0.196	−0.427	**	0.06	0.32	0.45	**
	CD141	0.038	0.42	−0.361	**	0.018	0.77	0.2	0.21
B cell	CD19	−0.132	**	−0.414	**	−0.0096	0.87	0.23	0.14
	CD20	0.005	0.917	−0.023	0.64	−0.014	0.82	0.31	*
	CD38	−0.023	0.621	−0.384	**	0.05	0.4	0.19	0.23
CD8^+^ T cell	CD8A	0.114	*	−0.397	**	0.22	**	0.043	0.79
	CD8B	0.01	0.832	0.221	**	0.05	0.41	-0.1	0.52
Th1	TBX21	0.123	**	−0.4	**	0.17	**	0.13	0.4
	STAT4	0.147	**	−0.377	**	0.12	0.053	0.095	0.55
	IL12RB2	0.113	*	−0.238	**	0.17	**	0.1	0.52
	IL27RA	0.039	0.408	−0.24	**	0.13	*	0.22	0.16
	STAT1	0.259	**	−0.274	**	0.29	**	0.096	0.55
	IFN-γ	0.176	**	−0.241	**	0.27	**	0.1	0.53
	TNF-α	−0.02	0.67	−0.255	**	0.022	0.72	-0.026	0.87
Th2	GATA3	−0.065	0.16	−0.364	**	-0.028	0.64	0.22	0.17
	CCR3	−0.068	0.15	−0.173	**	0.006	0.92	-0.25	0.12
	STAT6	−0.005	0.91	0.012	0.815	0.012	0.85	-0.03	0.85
	STAT5A	−0.096	*	−0.161	**	0.039	0.52	0.078	0.63
Th17	STAT3	0.187	**	−0.225	**	0.067	0.27	0.28	0.073
	IL-17 A	−0.064	0.175	−0.012	0.814	−0.038	0.53	0.27	0.092
	IL-21R	−0.023	0.626	−0.441	**	0.058	0.34	0.32	*
	IL-23R	0.024	0.61	0.023	0.65	−0.041	0.5	0.098	0.54
Treg	FOXP3	−0.095	*	−0.382	**	−0.038	0.53	0.26	0.1
	IL2RA	0.098	*	−0.401	**	0.11	0.075	0.4	**
	CCR8	0.014	0.76	−0.336	**	−0.029	0.63	0.33	*
Macrophage	CD68	0.031	0.51	−0.317	**	0.047	0.44	0.24	0.14
	CD11b	−0.006	0.89	−0.355	**	0.013	0.83	0.25	0.11

Note: None, Correlation without adjustment. Purity, correlation adjusted by purity. Cor, R value of Spearman’s correlation. *p < 0.05; **p < 0.01.

### Network Establishment for SLC7A11-Correlated Genes in COAD

**FIGURE 4:**
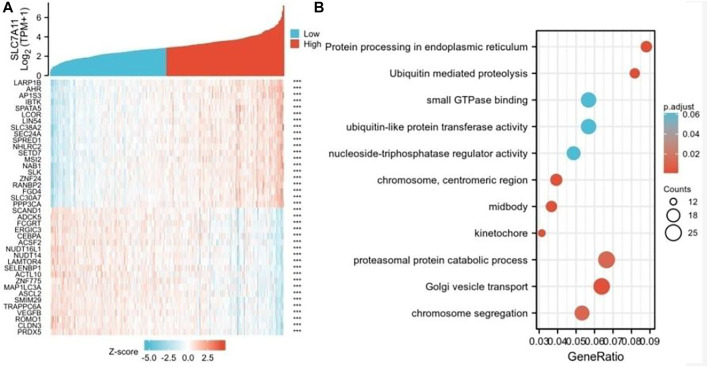
Genes analysis of closely associated with SLC7A11 in COAD. **(A)** The top 50 genes correlated with SLC7A11 in COAD are showed in the heatmap; ***p < 0.01. **(B)** GO–KEGG enrichment analysis of co-expression genes with SLC7A11 in COAD.

To further study the genes closely associated with SLC7A11 in COAD, the top 50 co-expressed genes were showed in a heatmap (Figure 4). Of note, 25 of 50 genes were negatively correlated with SLC7A11 (such as PRDX5, CLDN3, ROMO1, and VEGFB), and 25 genes were positively correlated (such as LARP1B,AHR,AP1S3,IBTK, and SPATA5) (Figure 4A). GO–KEGG enrichment analysis showed that the co-expression genes of SLC7A11 were mainly involved in the proteasomal protein catabolic process, Golgi vesicle transport, chromosome segregation, and other signaling pathways (Figure 4B).

### PPI and the TF-miRNA Co-regulatory Network

**FIGURE 5:**
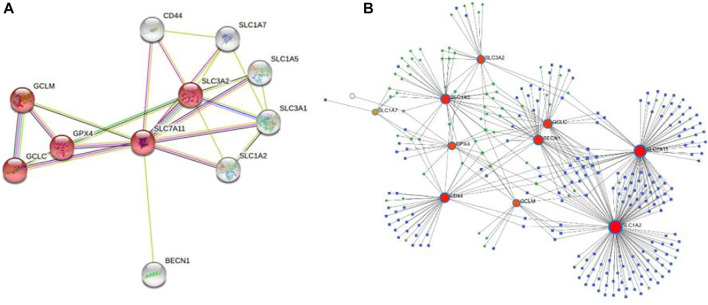
PPI and the co-regulatory network of SLC7A11. **(A)** SLC7A11-interaction proteins in COAD tissue. The network consists of 11 nodes and 23 edges, the red nodes are involved in the ferroptosis signaling pathway. **(B)** TF-miRNA co-regulatory network. The network consists of 270 nodes and 397 edges. The nodes in red color are the co-expression genes, the blue nodes represent miRNA, the green nodes represent TFs, and the yellow nodes represent protein.

To determine the role of SLC7A11 protein interactions in COAD progression, protein–protein interaction (PPI) was constructed by the STRING tool. The proteins that interact most closely with SLC7A11 include GPX4, GCLC, GCLM, SLC3A2, SLC1A7, SLC1A5, SLC3A1, SLC1A2, CD44, and BECN1; among which SLC7A11, GPX4, GCLC, GCLM, and SLC3A2 are involved in the ferroptosis signaling pathway (Figure 5A). Furthermore, 193 miRNAs were identified that regulated the expression level of co-expression genes by NetworkAnalyst analysis, and the main co-regulated genes were SLC7A11, SLC1A2, SLC1A5, BECN1, GCLC, and CD44. The miRNA that hit more frequently were hsa-mir-335-5p, hsa-mir-155-5p, hsa-mir-34a-5p, hsa-mir-16-5p, hsa-mir-20a-5p, hsa-mir-93-5p, hsa-mir-98-5p, and hsa-mir-24-3p. The TF that hit more frequently were RELA, SP1, NFKB1, TP53, BRCA1, JUN, and ESR1 (Figure 5B).

### Screening of Potential Therapeutic Drugs

Enrichr platform analysis found that F0447-0125 PC3 UP, semustine PC3 UP, securinine PC3 UP, alpha-tocopherol CTD 00007387, ampicillin CTD 00005394 *etc*. have been identified as drugs that regulate action for both SLC7A11 and co-expression genes (Table 3).

**TABLE 3:** Potential drugs that regulate action for both SLC7A11 and co-expression genes.

Drug	Adjusted *p*-value	Odds ratio	Combined score	Target genes
F0447-0125 PC3 UP	2.47E-06	369.94	3,921.13	SLC7A11 and GCLM
Semustine PC3 UP	2.47E-06	325.53	4,868.78	GCLC; SLC7A11; and GCLM
Securinine PC3 UP	2.99E-06	308.14	5,892.08	GCLC; SLC3A2; SLC7A11; and GCLM
Alpha-tocopherol CTD 00007387	3.51E-06	278.02	5,209.14	GCLC; SLC3A2; SLC7A11; and GCLM
Ampicillin CTD 00005394	1.75E-05	277.40	2,796.46	SLC7A11 and GCLM
Ebselen MCF7 UP	3.26E-05	277.40	2,796.46	SLC7A11 and GCLM
17-Ethynyl estradiol CTD 00005932	3.26E-05	241.43	3,410.20	GCLC; SLC7A11; and GCLM
N-acetyl-l-cysteine CTD 00005305	3.40E-05	237.39	4,306.60	GCLC; SLC3A2; SLC7A11; and GCLM
Glutathione CTD 00006035	4.90E-05	233.57	2,280.80	SLC7A11 and GCLM
Vanillin CTD 00003324	4.97E-05	221.88	2,145.57	SLC7A11 and GCLM

### Correlation of SLC7A11 Expression With Immune Characteristics

**FIGURE 6:**
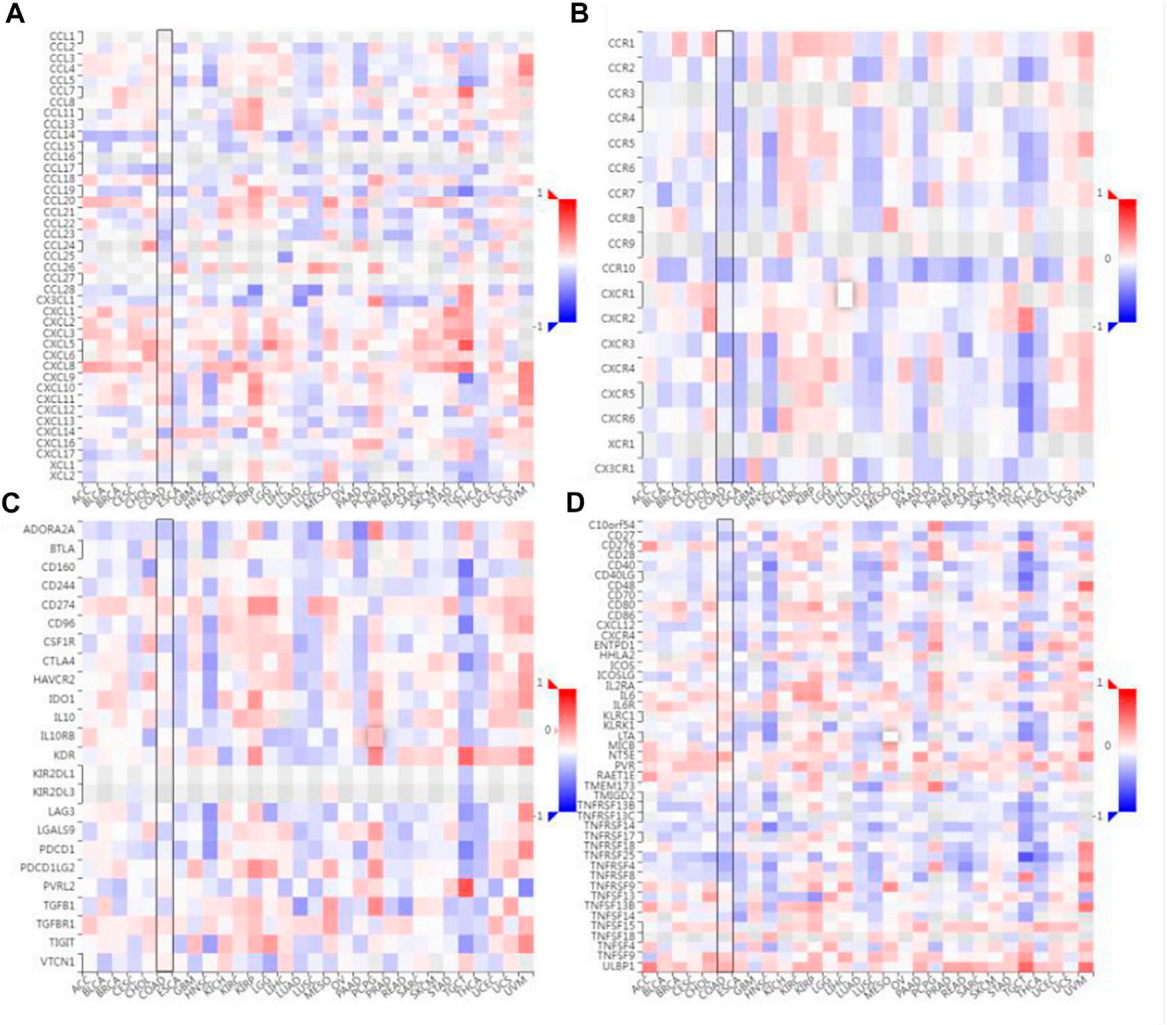
Correlation analysis between SLC7A11 expression and immunomodulators or chemokine receptors. **(A)** Heatmap analysis of the correlation between SLC7A11 and chemokines in tumors. **(B)** Heatmap analysis of the correlation between SLC7A11 and chemokine receptors in tumors. **(C)** Correlation between SLC7A11 and immunoinhibitors in tumors *via* heatmap analysis. **(D)** Correlation between SLC7A11 and immunostimulators in tumors by heatmap analysis.

The correlation between the expression level of SLC7A11 and immune cell chemokines (or receptors) in COAD were analyzed *via* the TISIDB database. The results showed that several chemokines and chemokine receptors were significantly correlated with the expression of SLC7A11 in COAD. Concretely, SLC7A11 expression was negatively correlated with CCL17 (*r* = -0.24 and *p* = 2.07e-07), CCL19 (*r* = -0.201 and *p* = 1.44e-05), CCL23 (*r* = -0.27 and *p* = 4.96e-09), CXCL14 (*r* = -0.289 and *p* = 3.25e-10), CCR10 (*r* = -0.33 and *p* = 5.11e-13), CX3CR1 (*r* = -0.203 and *p* = 1.2e-05), CXCR3 (*r* = -0.332 and *p* = 4.17e-13) *etc* (Figures 6A,B). Subsequently, the correlation between SLC7A11 and the expressions of immunoinhibitors (or immunostimulators) in different cancers were analyzed. The results showed that SLC7A11 was negatively correlated with the expression of several immunoinhibitors and immunostimulators, such as ADORA2A (*r* = −0.224, *p* = 1.36e-06), CSF1R (*r* = −0.176, *p* = 1.54e-04), TGFB1 (*r* = −0.162, *p* = 4.95e-04), CD27 (*r* = −0.247, *p* = 9.01e-08), LTA (*r* = −0.181, *p* = 1.01e-04), TMIGD2 (*r* = −0.141, *p* = 2.47e-03), TNFRSF13B (*r* = −0.248, *p* = 7.64e-08) et al. Interestingly, SLC7A11 was positively correlated with the expression of several immunostimulators, such as TNFSF9 (*r* = 0.209, *p* = 6.41e-06), ULBP1 (*r* = 0.303, *p* = 4.09e-11), and NT5E (*r* = 0.337, *p* = 1.59e-13) (Figures 6C,D). Therefore, these results suggest that SLC7A11 may play a role in regulating tumor immunity.

### Overexpressed SLC7A11-Inhibited Chemokine and Receptors Expression

**FIGURE 7:**
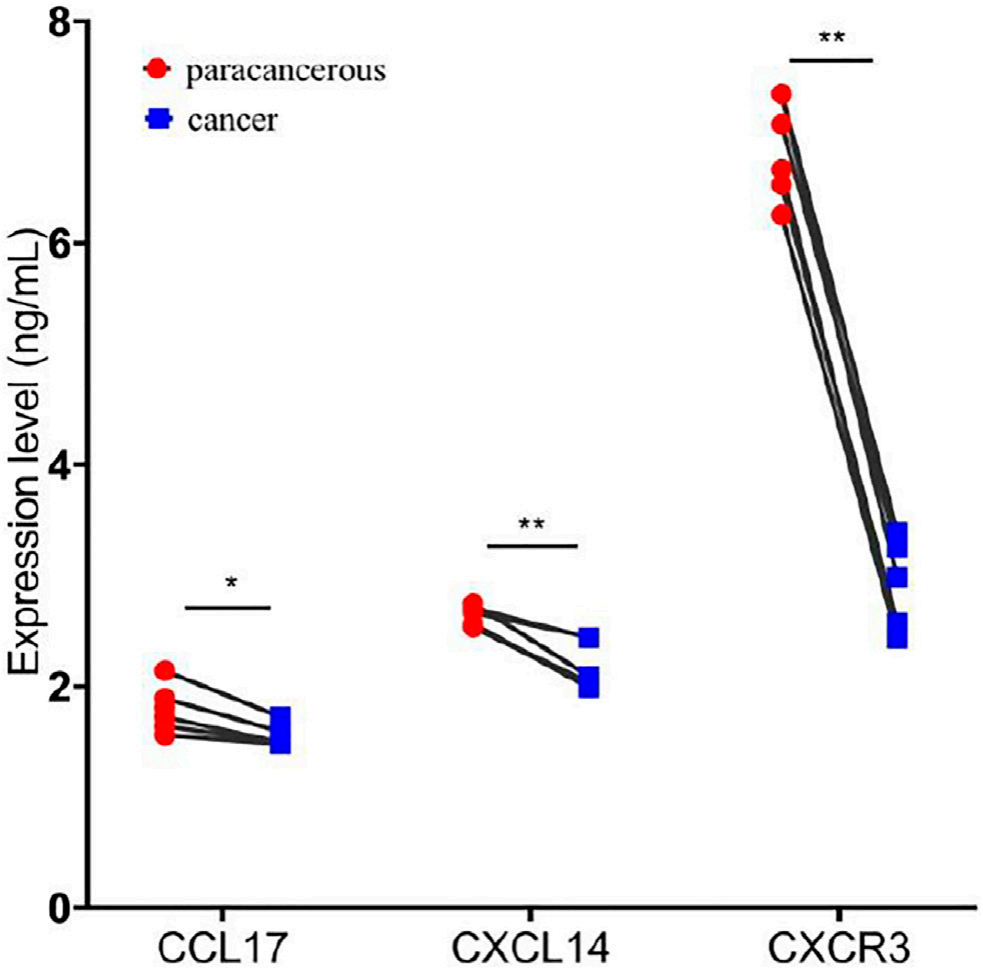
Overexpression of SLC7A11 inhibits the expression of chemokines and chemokine receptors. *n* = 6, *p < 0.05, and **p < 0.01.

For validation, we examined the expression of CCL17, CXCL14, and CXCR3 in cancer and adjacent tissues of six patients with SLC7A11 overexpression by ELISA. The results showed that the levels of CCL17, CXCL14, and CXCR3 in cancer were 1.558 ± 0.104 ng/ml, 2.169 ± 0.214 ng/ml, and 2.843 ± 0.423 ng/ml, respectively, significantly lower than the corresponding adjacent tissues 1.795 ± 0.207 ng/ml, 2.625 ± 0.093 ng/ml, and 6.868 ± 0.455 ng/ml (*p* < 0.05 or 0.01, Figure 7).

## Discussion

Despite adjuvant chemotherapy, immunotherapy, and other anticancer therapies in early cancer treatment have shown encouraging efficacy, the middle and later stages of treatment in most cases are challenging. Therefore, it is necessary and urgent to find a more effective strategy to treat cancer. The ideal therapeutic target to be inhibited by an anticancer drug should have a selective effect on tumor growth, allowing the corresponding drug to produce the desired toxic effect in cancer cells without side effects on normal cells. Ferroptosis is a newly discovered mechanism of iron-dependent cell death, characterized by increased reactive oxygen species (ROS) and lipid peroxidation due to metabolic dysfunction, which is considered as a powerful weapon in the elimination of cancer cells (Tang et al., 2020). SLC7A11 is a core target-regulating ferroptosis, and its overexpression leads to downregulation of the sensitivity of cancer cells to ferroptosis. Recent research suggests that cancer therapies, such as immunotherapy and radiotherapy, can induce ferroptosis partly through modulating SLC7A11 expression (Lang et al., 2019; Lei et al., 2020); knockout or inhibition of SLC7A11 has a significant inhibitory effect on lung cancer and pancreatic cancer cells (Ji et al., 2018; Sharbeen et al., 2021). SLC7A11 has become a central hub linking ferroptosis to its suppressive function on tumor.

The study showed that SLC7A11 is essential to elicit tumor formation and maintain tumorigenicity by relieving oxidative stress in COAD, pancreatic ductal adenocarcinoma (PDAC), and lung adenocarcinoma (LUAD) (Lim et al., 2019). This study found that SLC7A11 is highly expressed in most cancers, such as COAD, LUAD, and esophageal cancer (ESCA). This notion is further supported by the GEO database. The results indicated that many cases may shrink ferroptosis by upregulating SLC7A11 expression. Interestingly, the expression of SLC7A11 was relatively stable in all pathological stages of COAD, but decreased after lymphatic invasion. These results suggest that the overexpression of SLC7A11 promotes lymphatic metastasis of tumor cells, which was also confirmed in pancreatic carcinoma (Zhu et al., 2020).

Our study demonstrated that the expression level of SLC7A11 has a significant correlation with the infiltration levels of CD8^+^ T cells, neutrophils, and dendritic cells in COAD. Studies show that immunotherapy-activated CD8^+^ T cells enhance ferroptosis-specific lipid peroxidation in tumor cells, and that increased ferroptosis contributes to the antitumor efficacy of immunotherapy (Wang et al., 2019). Further analysis of infiltrated lymphocyte markers showed that the markers of Th1 cell such as TBX21, IL12RB2, IL27RA, STAT1, and IFN-γ were strongly correlated with SLC7A11 expression, as well as the CD8^+^ T cell marker CD8A and the NK cell marker KIR3DL1. Intriguingly, IFN-γ upregulates the level of intracellular Fe^2+^ and decreases the level of GPX4, which lead the cells to be more sensitive to ferroptosis; STAT1 inhibitors could reverse the reduction of SLC7A11 expression induced by IFN-γ and induces ferroptosis *via* activation of the JAK1-2/STAT1/SLC7A11 signaling pathway (Wei et al., 2021). Meanwhile, SLC7A11 also affects the infiltration of immune cells in rectal cancer, such as CD8^+^ T cells (Supplementary Figure S1). These results further implied that SLC7A11 was the key molecule for bridging the ferroptosis process and immunotherapy. Moreover, we noticed that most of the immune cells or their markers were weak in the prediction of correlations *via* the TIMER and GEPIA databases. A similar situation has occurred in some recent studies (Zhao et al., 2021). This weak correlation does not mean that the detected target molecule can be ignored. If the correlations of target molecules, which are considered to be highly reliable, are calculated with consistent results in different databases (positive or negative), and *p* values indicate significant differences.

SLC7A11 is a key gene regulating ferroptosis and is overexpressed in COAD. Further study found that the genes closely associated with SLC7A11, such as PRDX5, CLDN3, ROMO1, and VEGFB genes were also overexpressed; LARP1B,AP1S3, IBTK, and SPATA5 genes were under-expressed. PRX5 can promote epithelial-to-mesenchymal transition (EMT) properties by inducing the expression of EMT-inducing transcription factors in colorectal cancer (Ahn et al., 2017). Li, et al. study found that CLDN3 is overexpressed in colorectal cancer tissues, and its high expression may promote the occurrence and progression of colorectal cancer (Li et al., 2017). VEGFB overexpression is thought to be highly associated with poor prognosis (Lautenschlaeger et al., 2013). In contrast, among the genes that are under-expressed, IBTK inhibits the survival and proliferation of tumor cells by influencing the tumor microenvironment (Pal Singh et al., 2018). These results suggest that SLC7A11 and its co-expressed gene may be an important gene promoting the development of COAD.

TFs-miRNAs can jointly regulate target gene expression in the forms of feed-forward loops or feedback loops; these regulatory loops play an important role in gene regulatory networks and disease processes (Zhang et al., 2015). In this study, miRNA (such as hsa-mir-335-5p, hsa-mir-155-5p, and hsa-mir-34a-5p) and TFs (such as RELA, SP1, and NFKB1) with high frequency of action were identified. Studies have shown that mir-335-5P mediates the proliferation, apoptosis, and invasion of osteosarcoma and breast cancer cells (Chen et al., 2018; Zhang et al., 2021). RELA of NF-κB-driven cytokine by myeloid cells is required for colitis-associated cancer growth (Greten et al., 2004). Therefore, it is assumed that targeting TFs and miRNAs to regulate the expression of SLC7A11 may have a potential role in the treatment of COAD.

The construction of the PPI network is of great significance in analyzing protein signal transduction, gene expression regulation, and functional relationship among proteins. According to the PPIs network, SLC7A11, GPX4, GCLC, GCLM, and SLC3A2 were declared as hub genes involved in ferroptosis. Further examination of the hub genes of the PPI network revealed that these genes were also highly expressed in COAD (Supplementary Figure S2). As can be seen from the ferroptosis signaling pathway Supplementary Figure S3), SLC7A11 is the upstream gene, and its expression level is positively correlated with hub genes. These results further suggest that SLC7A11 was a central regulator of ferroptosis in COAD. Furthermore, securinine PC3 UP, alpha-tocopherol CTD 00007387, and N-acetyl-l-cysteine CTD 00005305 are the peak drug candidates for regulating hub genes. Growing evidence suggest that securinine and alpha-tocopherol have anticancer and antimetastasis properties (Middha et al., 2019; Ashraf et al., 2021).

Chemokines and chemokine receptors are essential for the infiltration of immune cells into tumors. In this study, it is proved that the expression level of SLC7A11 was negatively correlated with the expressions of CCL17, CCL19, CCL22, CCL23, CXCL14, CCR10, CX3CR1, and CXCR3, suggesting that the high expression of SLC7A11 may inhibit the migration of immune cells. In contrast, the SLC7A11 expression level was positively correlated with the expressions of CXCL1-6/8-11. Studies have shown that CXCL9/10/11 are the main chemokines of CD8^+^ T cells (Shigeta et al., 2020; Marcovecchio et al., 2021); CXCL2/8 are the main chemokines of neutrophil (Xu et al., 2021). This further confirmed the results of immune cell infiltration. However, a high proportion of neutrophils can inhibit the function of immune cells such as CD8^+^ T cells, and promote tumor growth (Zhou et al., 2018). This may explain the poor immunotherapy response in patients with SLC7A11 high expression. CCL17 and CCL22 are the ligands for CCR4, which effect on the recruitment of Treg, Th2, and Th17 into the tumor (Korbecki et al., 2020). In addition, they exert an anticancer effect by causing the infiltration of tumor-infiltrating lymphocytes (TIL) into the tumor (Kanagawa et al., 2007). Chemokine CXCL14 is a key regulatory factor in cancer and represents a potential target for future cancer immunotherapies (Westrich et al., 2020). The CXCR3 and CX3CR1 are mainly responsible for the tumor-suppressive lymphocytic infiltration into the tumor micromilieu (Bronger et al., 2019). Interestingly, the high expression of SLC7A11 also affects the expression of immunoinhibitors and immunostimulators. Therefore, these results suggest that SLC7A11 may play a role in regulating tumor immunity.

## Conclusion

In summary, our study found that the expression of SLC7A11 and genes interact with it are significantly upregulated in COAD, and results in more GSH synthesis. GPX4 uses GSH to improve the antioxidant activity of tumor cells, thereby suppressing ferroptosis. Meanwhile, the overexpression of SLC7A11 can affect the expression of chemokines, leading to the infiltration of immune cells such as CD8^+^ T cells/neutrophils, and deficiency of other immune cells, and causes immunosuppression of tumor cells. Regulation of SLC7A11 expression would be a potential therapeutic approach in inducing ferroptosis and/or immunotherapy for tumors.

## Data Availability

The original contributions presented in the study are included in the article/Supplementary Material; further inquiries can be directed to the corresponding author.
