# Supplementary figures and images for: SLC7A11, a Potential Therapeutic Target Through Induced Ferroptosis in Colon Adenocarcinoma
Source: Front Mol Biosci. 2022 Apr 20;9:889688. doi: 10.3389/fmolb.2022.889688 (PMC9065265; doi:10.3389/fmolb.2022.889688)

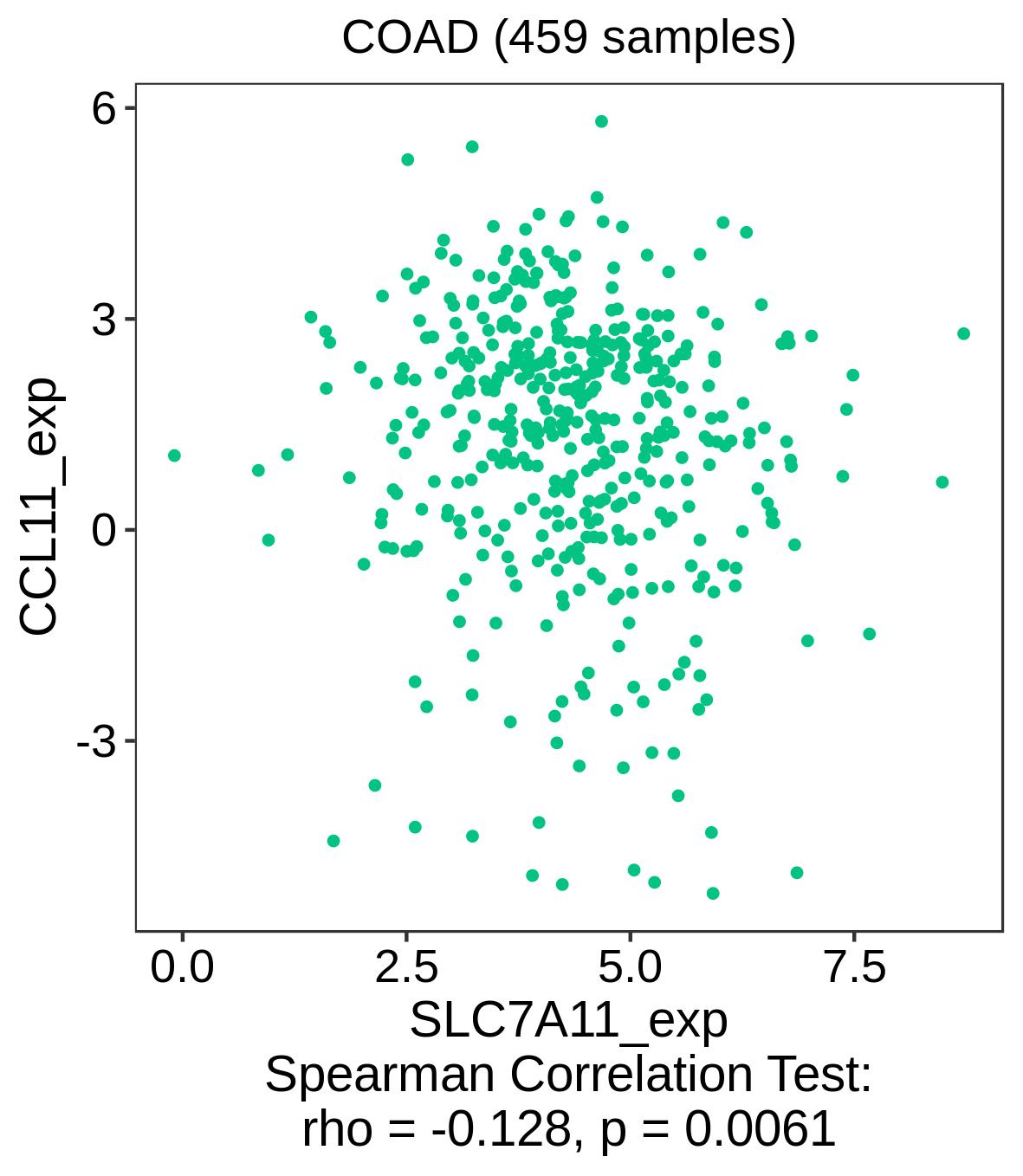

Supplement: Supplementary file 1 [file DataSheet1.ZIP › Raw data/original data/Figure 6 Immune Characteristics/Figure. 6A chemokines/SLC7A11_exp_COAD_chemokine_CCL11.jpg]

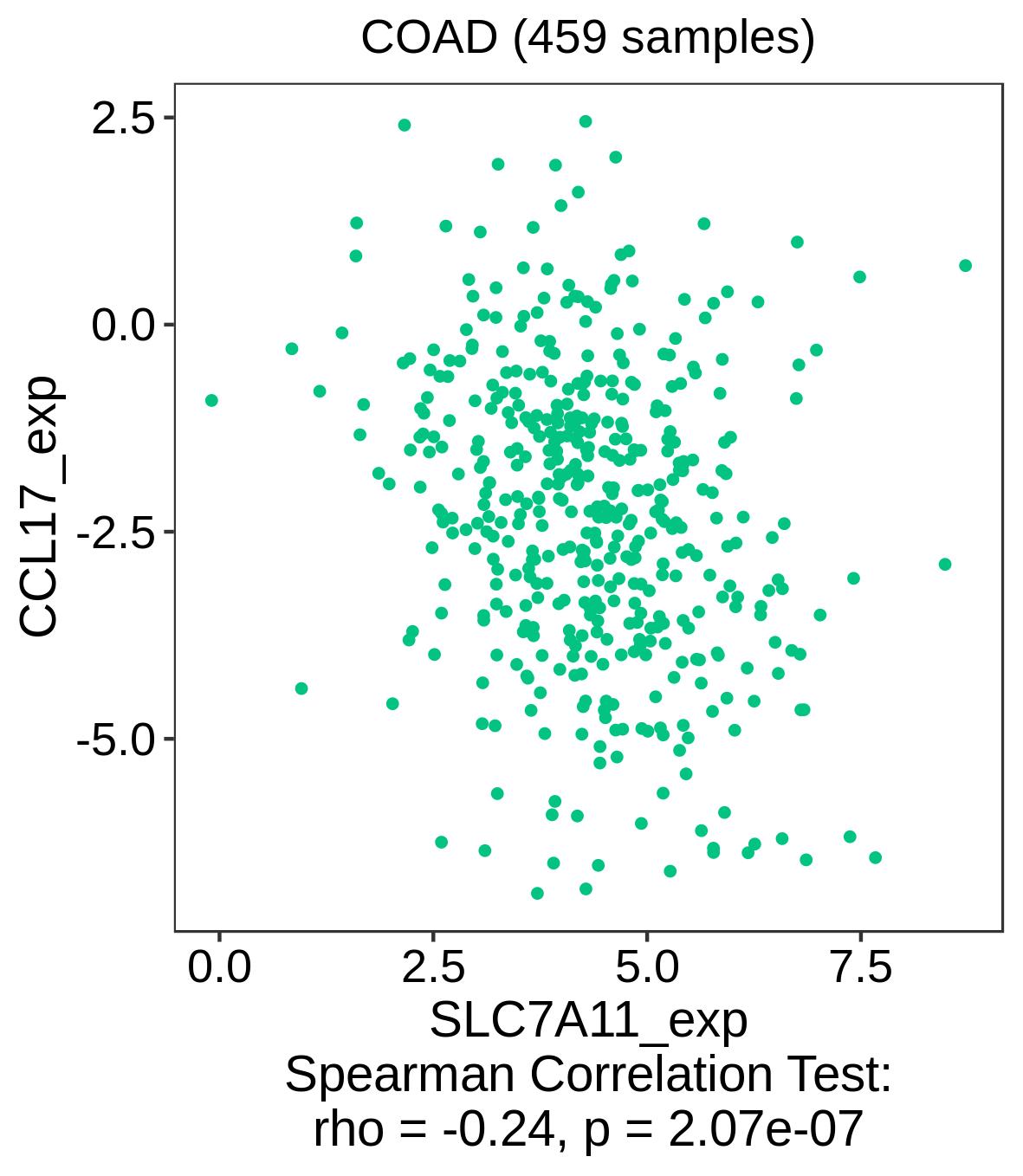

Supplement: Supplementary file 1 [file DataSheet1.ZIP › Raw data/original data/Figure 6 Immune Characteristics/Figure. 6A chemokines/SLC7A11_exp_COAD_chemokine_CCL17.jpg]

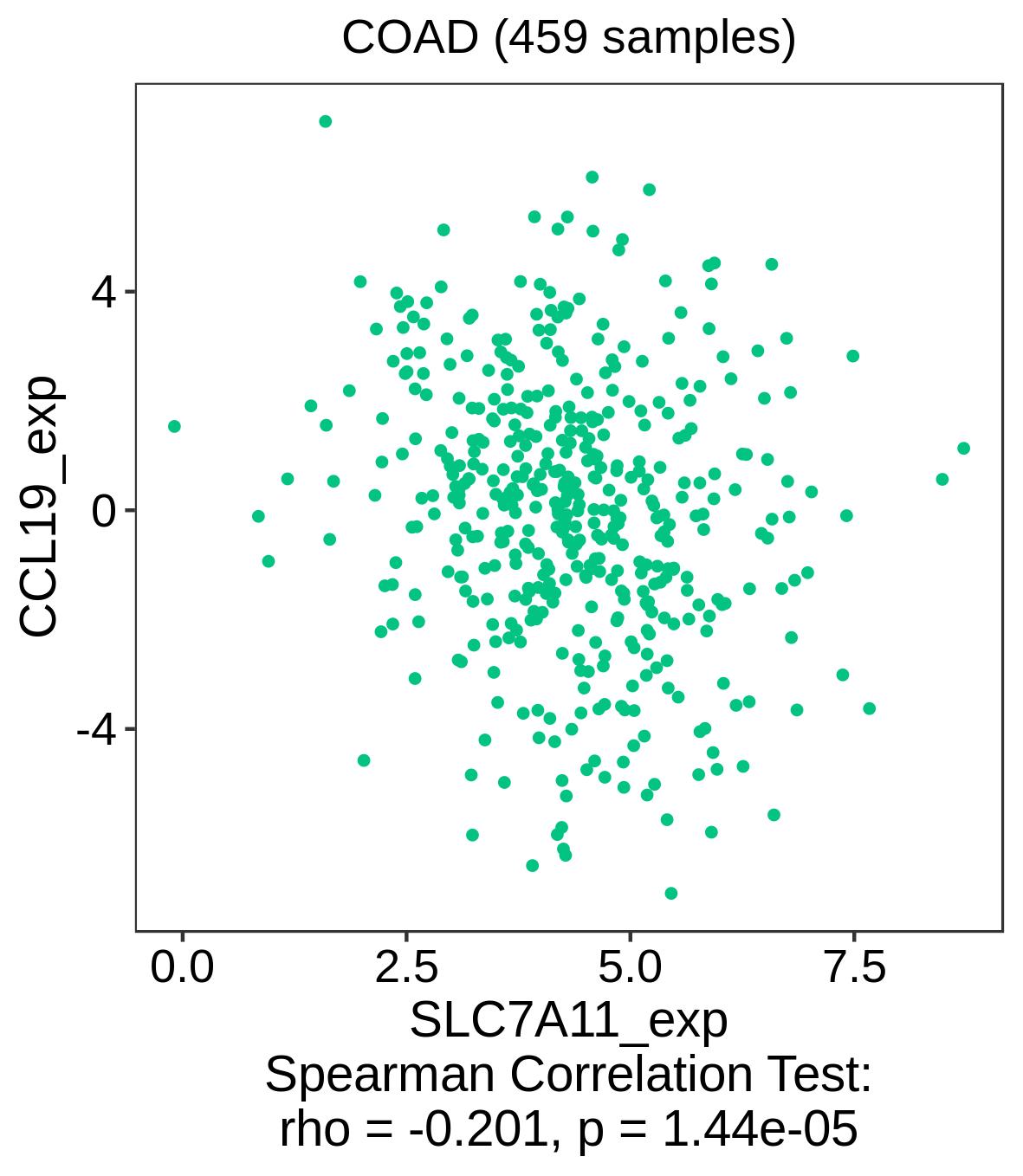

Supplement: Supplementary file 1 [file DataSheet1.ZIP › Raw data/original data/Figure 6 Immune Characteristics/Figure. 6A chemokines/SLC7A11_exp_COAD_chemokine_CCL19.jpg]

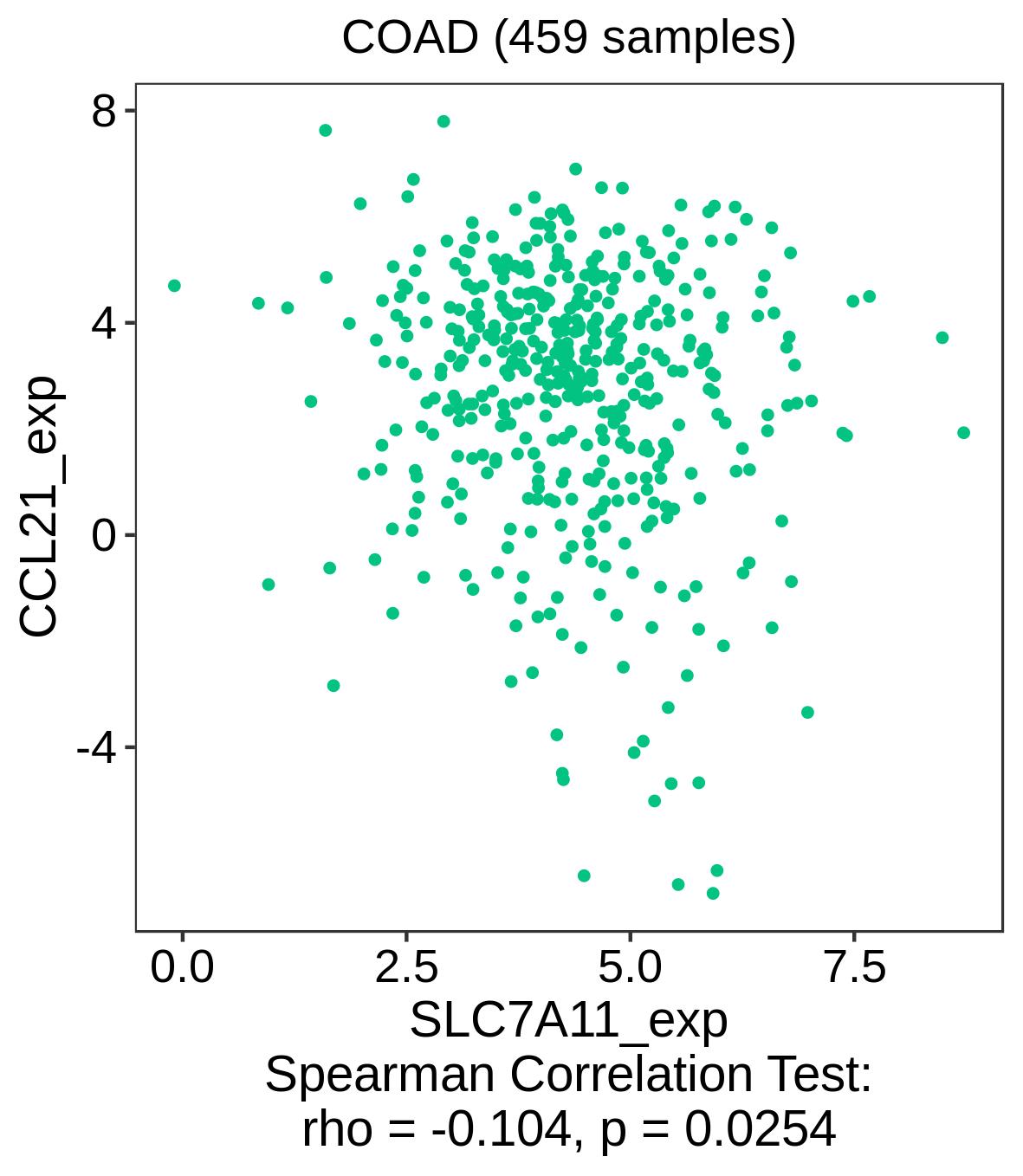

Supplement: Supplementary file 1 [file DataSheet1.ZIP › Raw data/original data/Figure 6 Immune Characteristics/Figure. 6A chemokines/SLC7A11_exp_COAD_chemokine_CCL21.jpg]

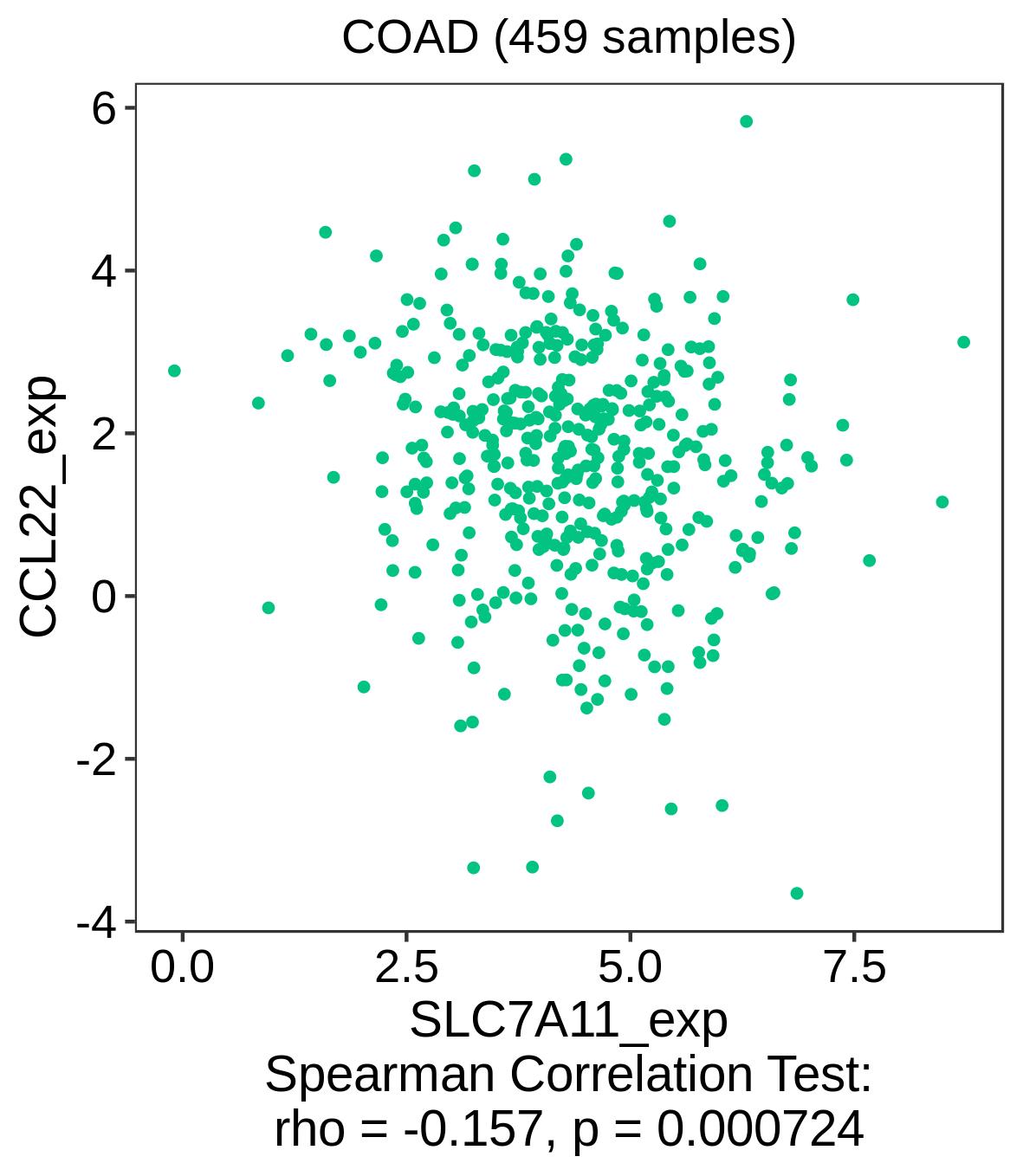

Supplement: Supplementary file 1 [file DataSheet1.ZIP › Raw data/original data/Figure 6 Immune Characteristics/Figure. 6A chemokines/SLC7A11_exp_COAD_chemokine_CCL22.jpg]

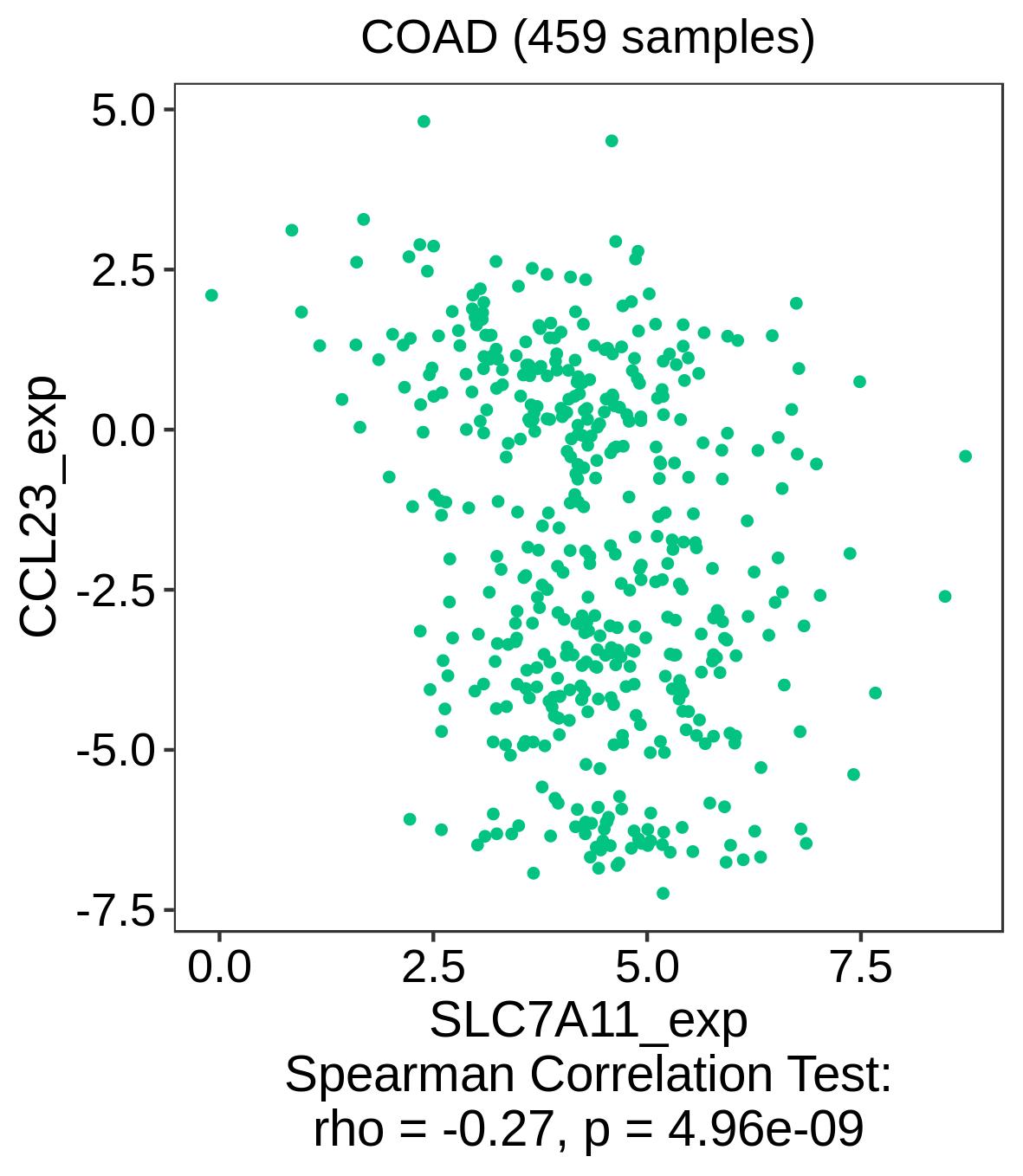

Supplement: Supplementary file 1 [file DataSheet1.ZIP › Raw data/original data/Figure 6 Immune Characteristics/Figure. 6A chemokines/SLC7A11_exp_COAD_chemokine_CCL23.jpg]

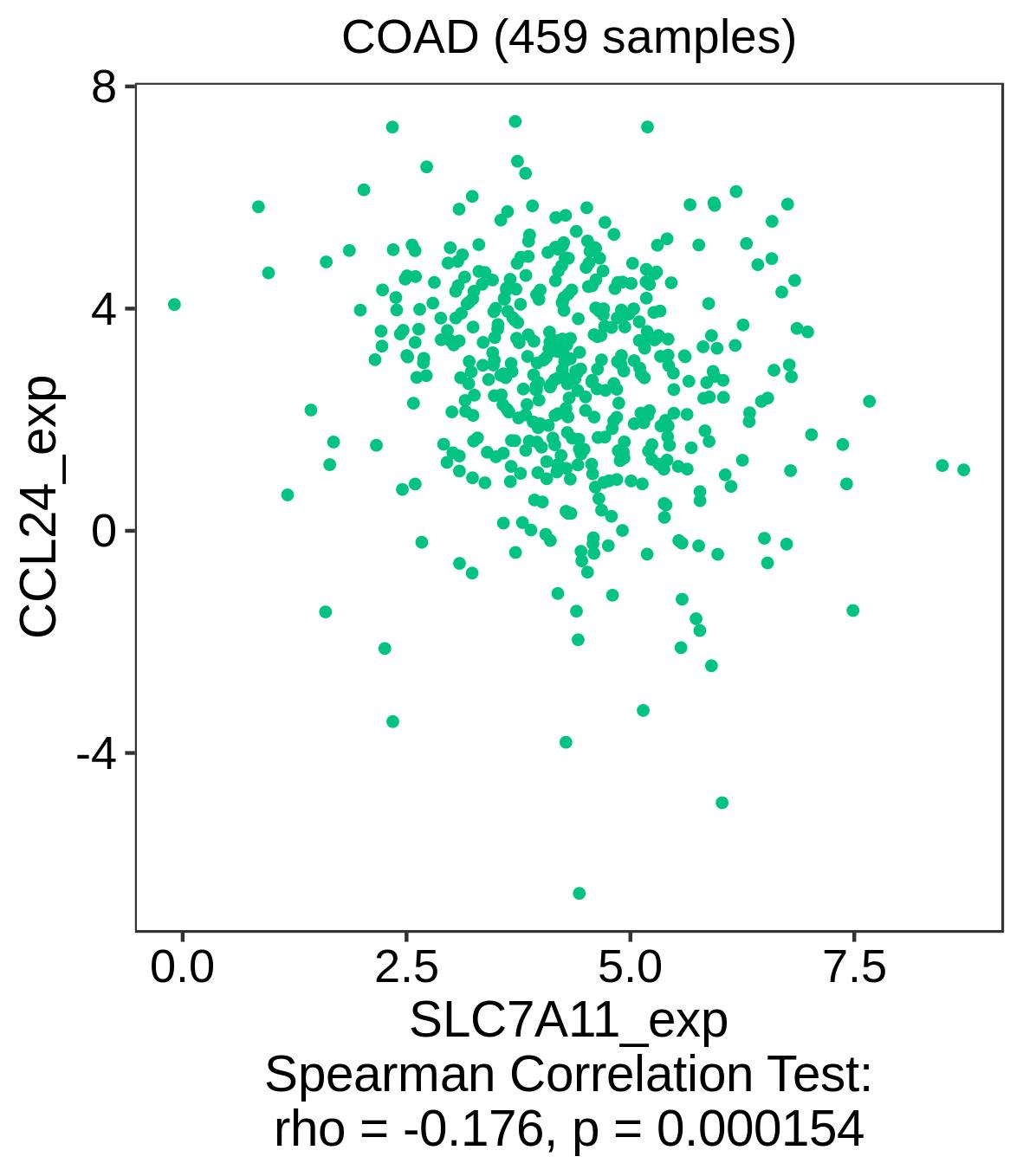

Supplement: Supplementary file 1 [file DataSheet1.ZIP › Raw data/original data/Figure 6 Immune Characteristics/Figure. 6A chemokines/SLC7A11_exp_COAD_chemokine_CCL24.jpg]

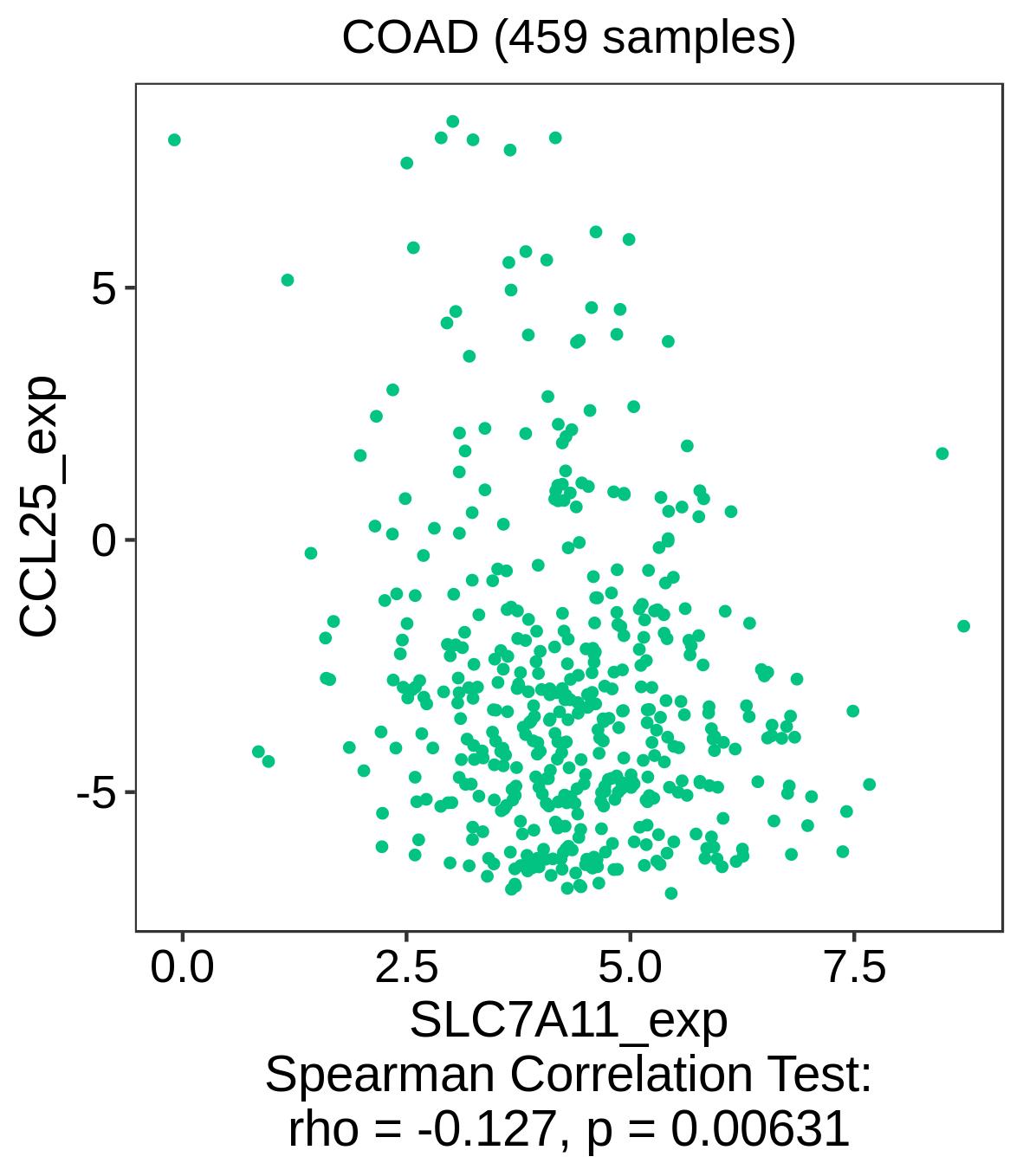

Supplement: Supplementary file 1 [file DataSheet1.ZIP › Raw data/original data/Figure 6 Immune Characteristics/Figure. 6A chemokines/SLC7A11_exp_COAD_chemokine_CCL25.jpg]

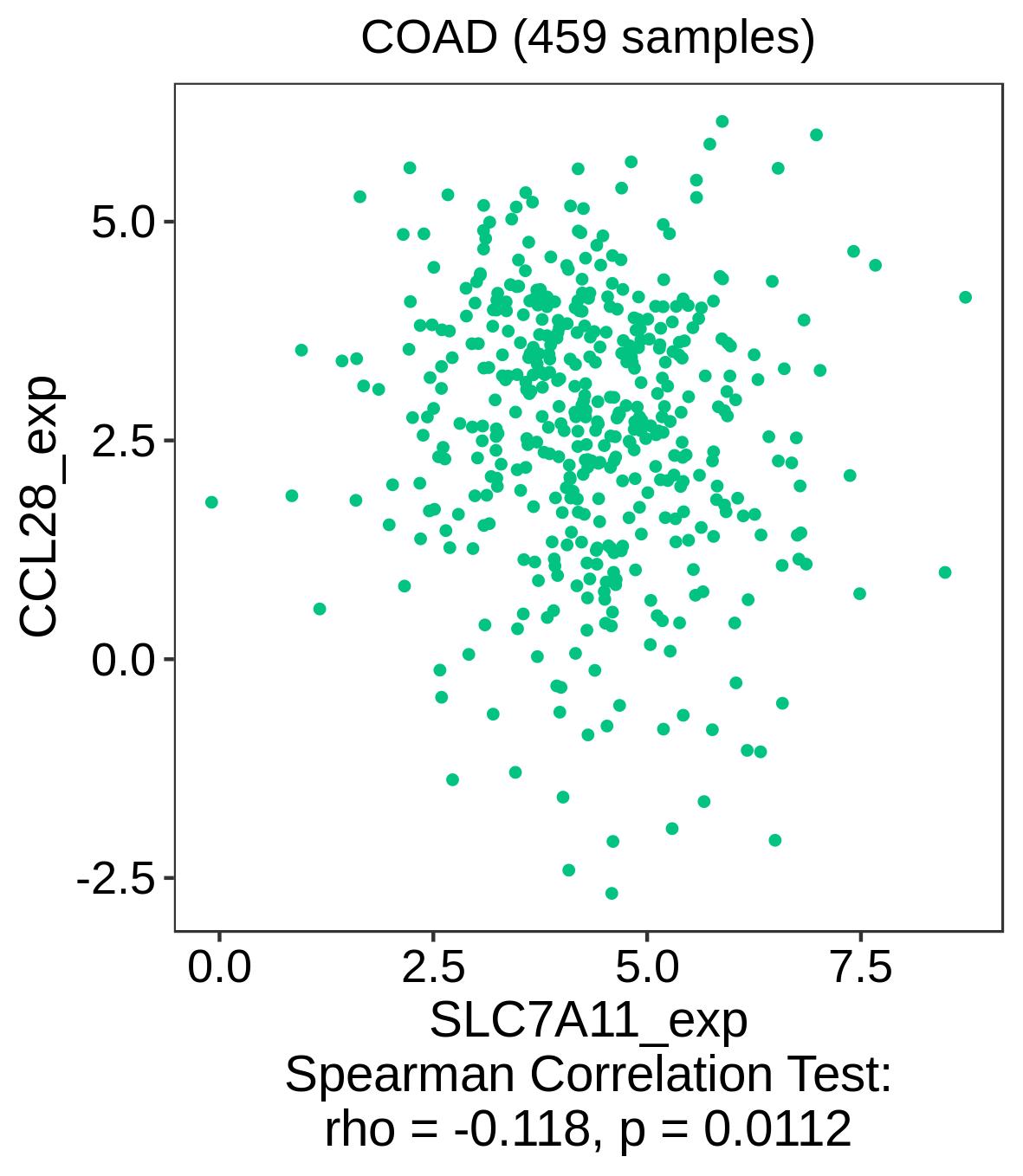

Supplement: Supplementary file 1 [file DataSheet1.ZIP › Raw data/original data/Figure 6 Immune Characteristics/Figure. 6A chemokines/SLC7A11_exp_COAD_chemokine_CCL28.jpg]

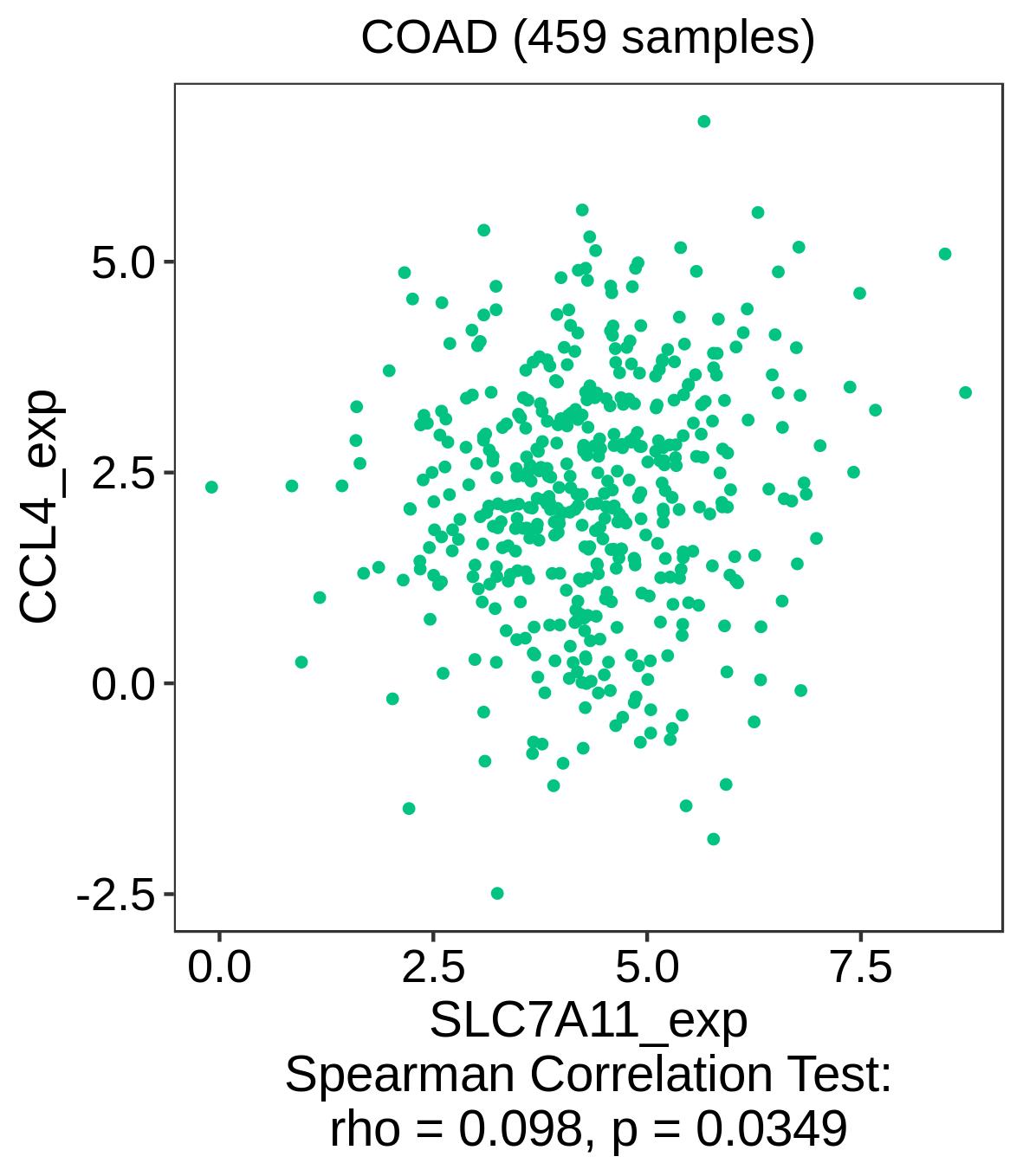

Supplement: Supplementary file 1 [file DataSheet1.ZIP › Raw data/original data/Figure 6 Immune Characteristics/Figure. 6A chemokines/SLC7A11_exp_COAD_chemokine_CCL4.jpg]

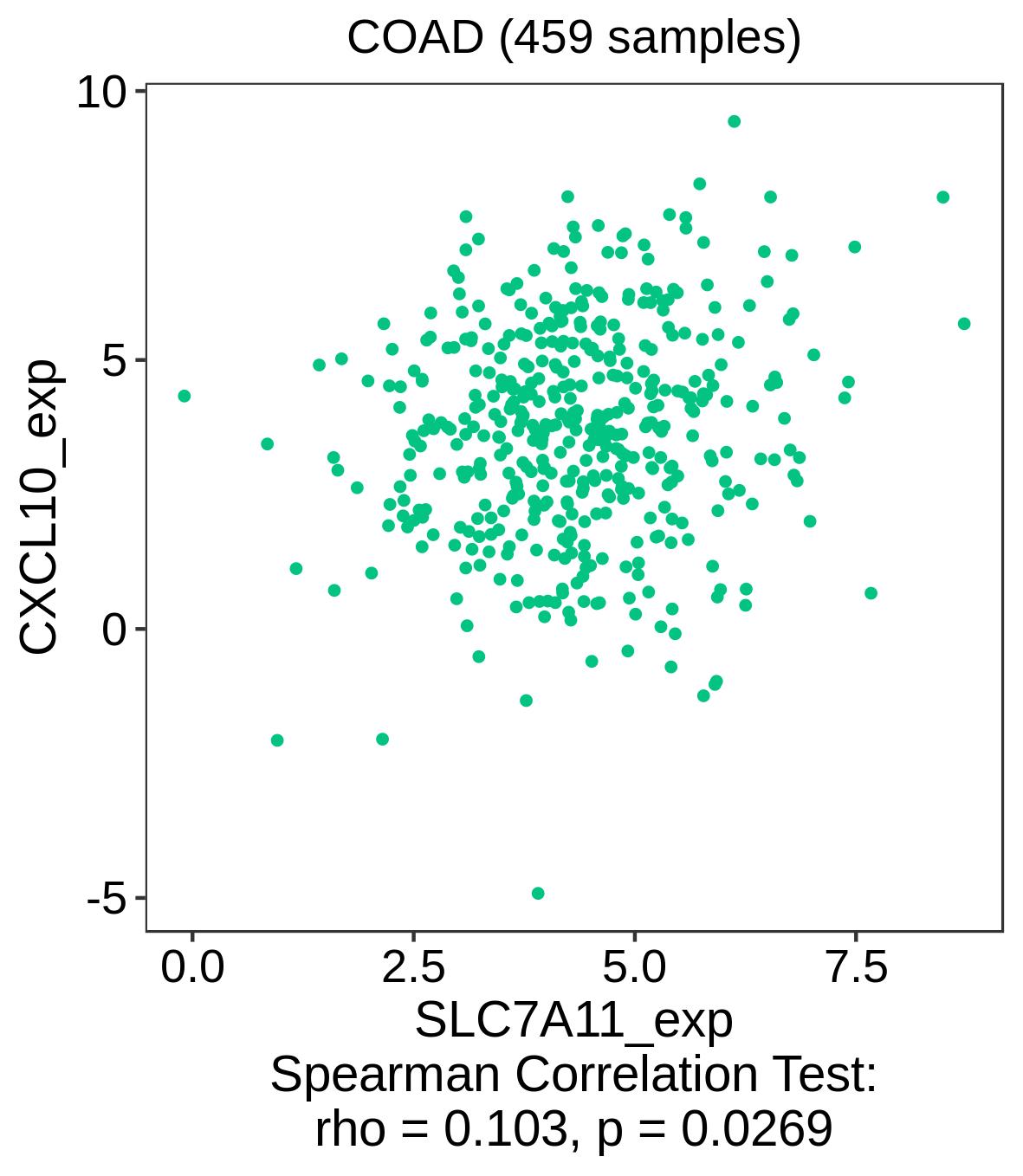

Supplement: Supplementary file 1 [file DataSheet1.ZIP › Raw data/original data/Figure 6 Immune Characteristics/Figure. 6A chemokines/SLC7A11_exp_COAD_chemokine_CXCL10.jpg]

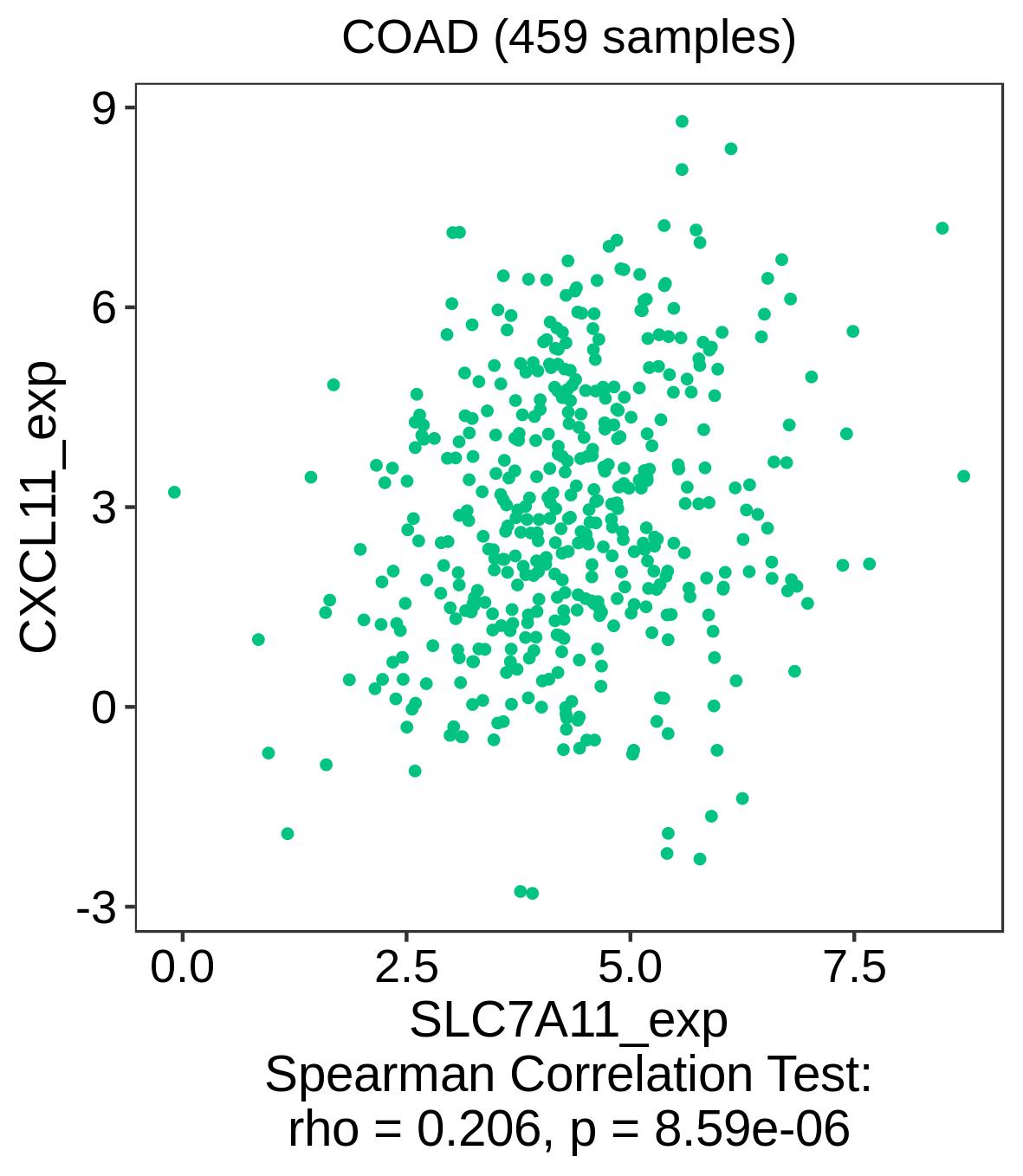

Supplement: Supplementary file 1 [file DataSheet1.ZIP › Raw data/original data/Figure 6 Immune Characteristics/Figure. 6A chemokines/SLC7A11_exp_COAD_chemokine_CXCL11.jpg]

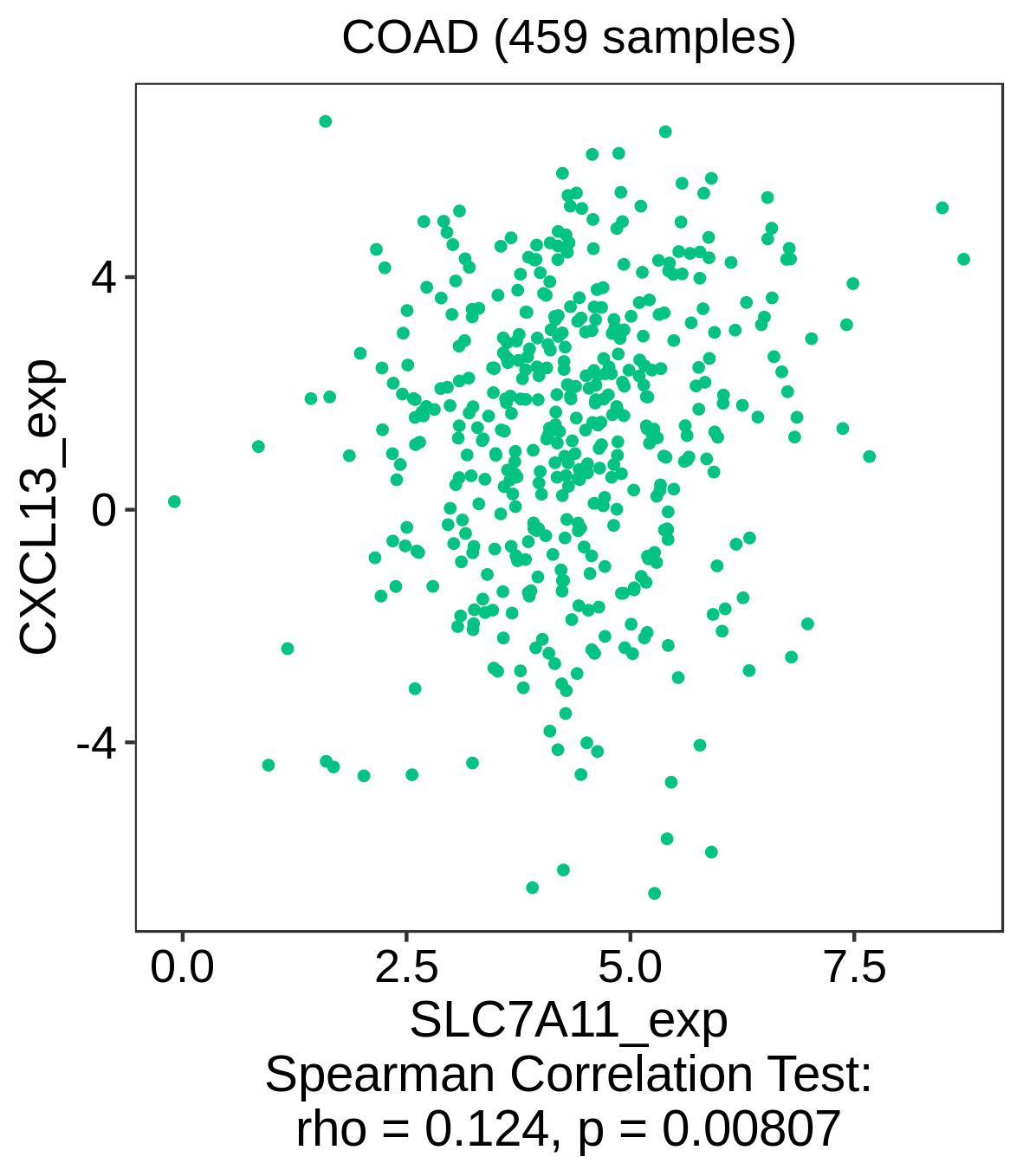

Supplement: Supplementary file 1 [file DataSheet1.ZIP › Raw data/original data/Figure 6 Immune Characteristics/Figure. 6A chemokines/SLC7A11_exp_COAD_chemokine_CXCL13.jpg]

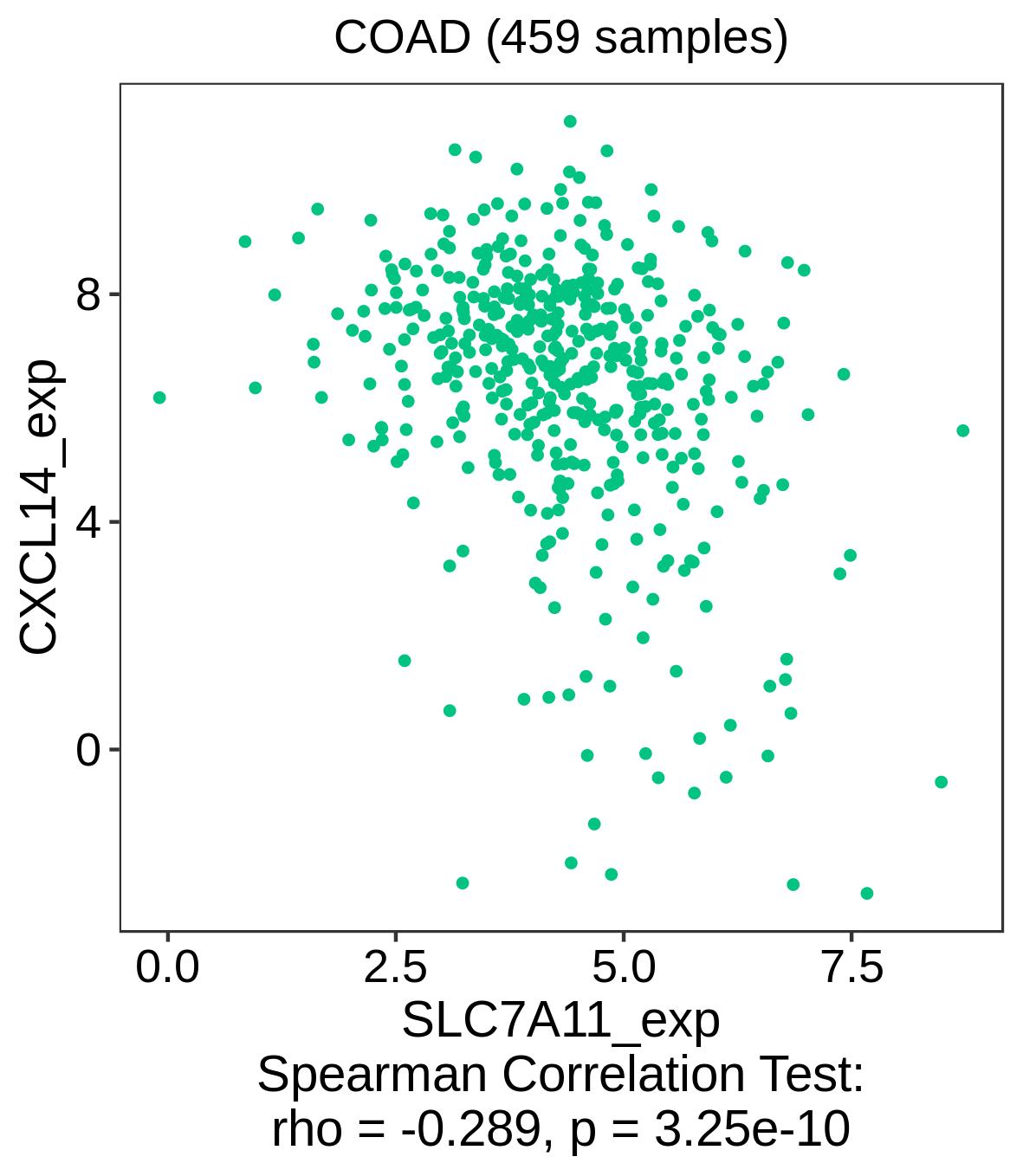

Supplement: Supplementary file 1 [file DataSheet1.ZIP › Raw data/original data/Figure 6 Immune Characteristics/Figure. 6A chemokines/SLC7A11_exp_COAD_chemokine_CXCL14.jpg]

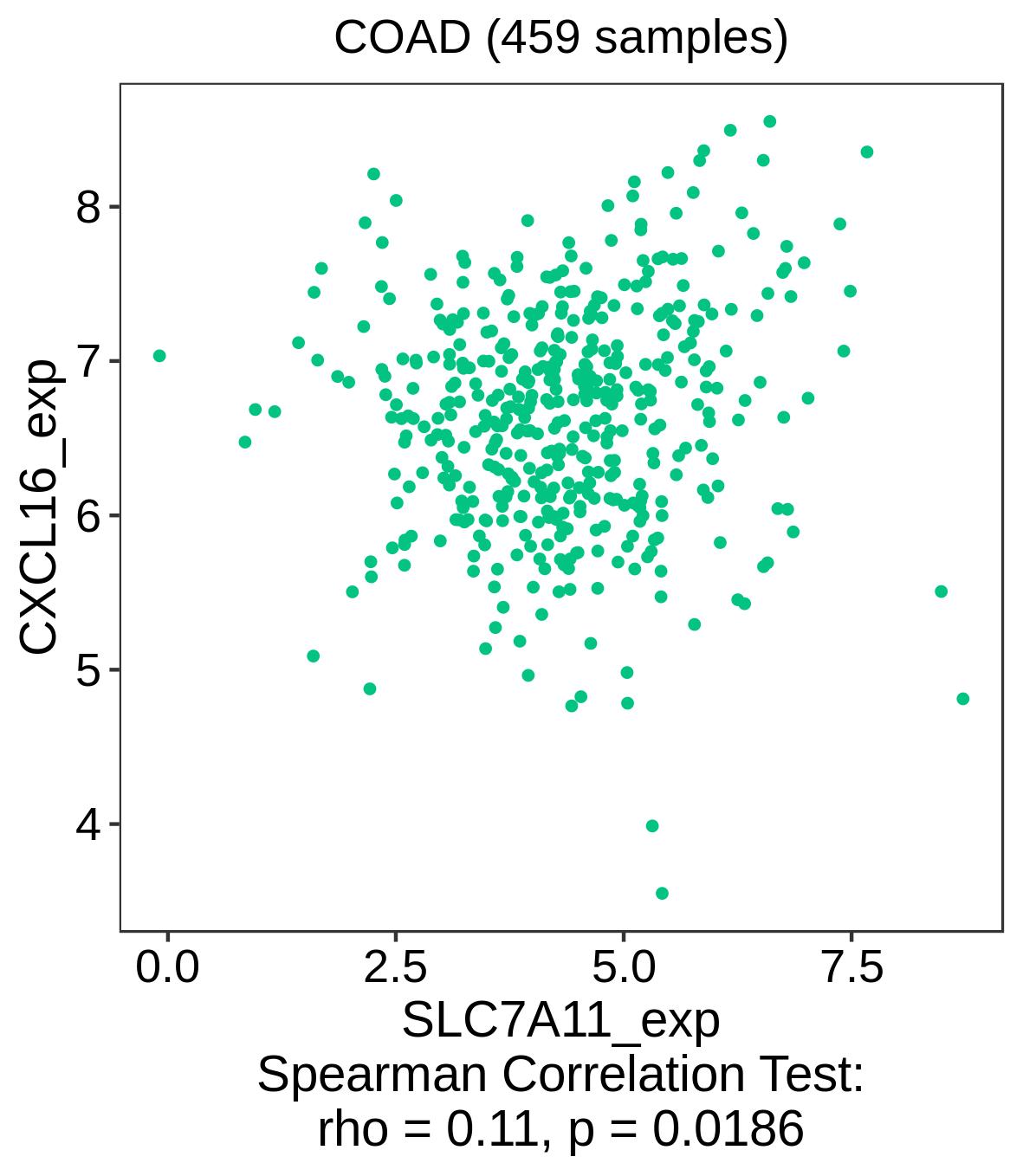

Supplement: Supplementary file 1 [file DataSheet1.ZIP › Raw data/original data/Figure 6 Immune Characteristics/Figure. 6A chemokines/SLC7A11_exp_COAD_chemokine_CXCL16.jpg]

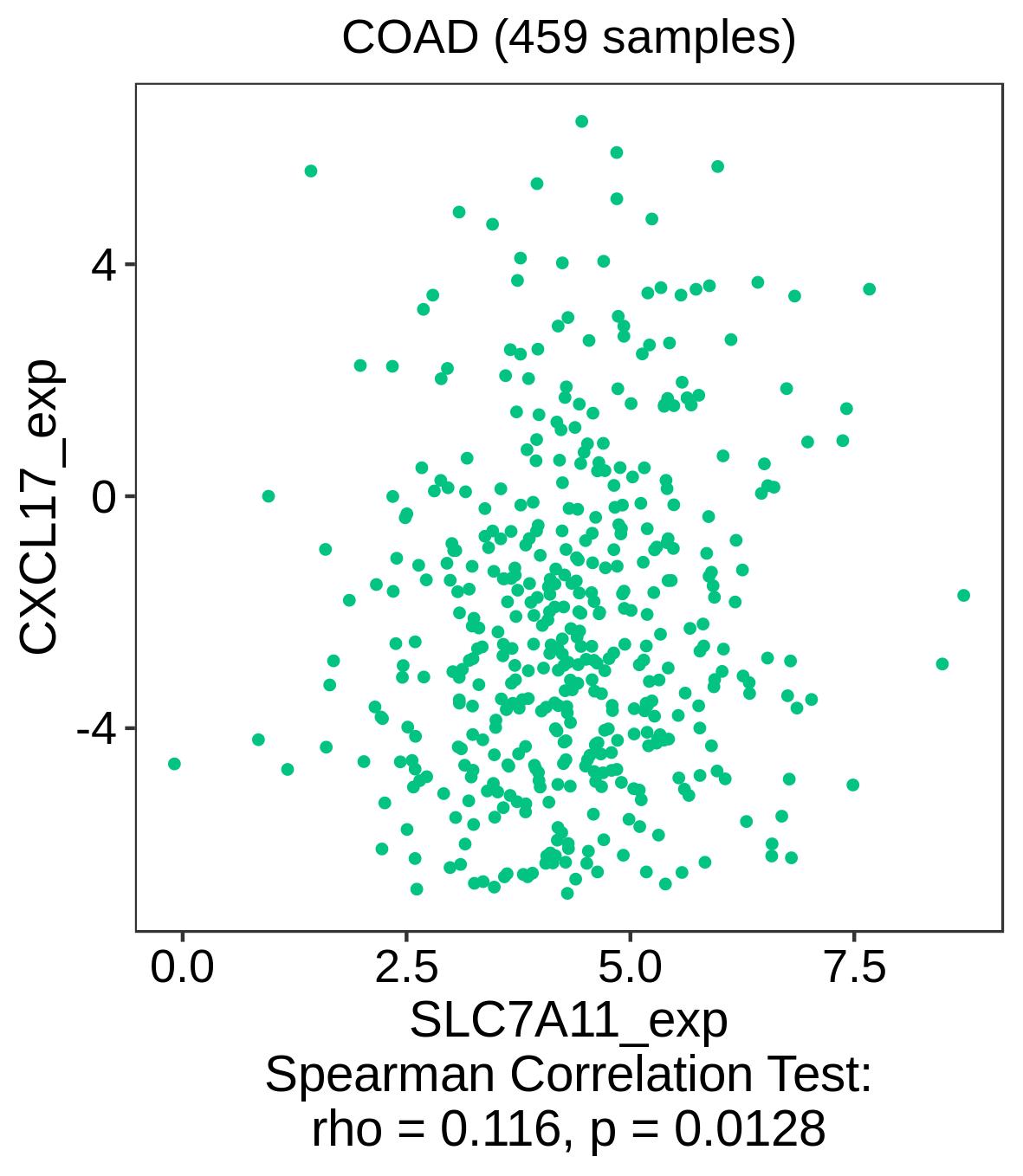

Supplement: Supplementary file 1 [file DataSheet1.ZIP › Raw data/original data/Figure 6 Immune Characteristics/Figure. 6A chemokines/SLC7A11_exp_COAD_chemokine_CXCL17.jpg]

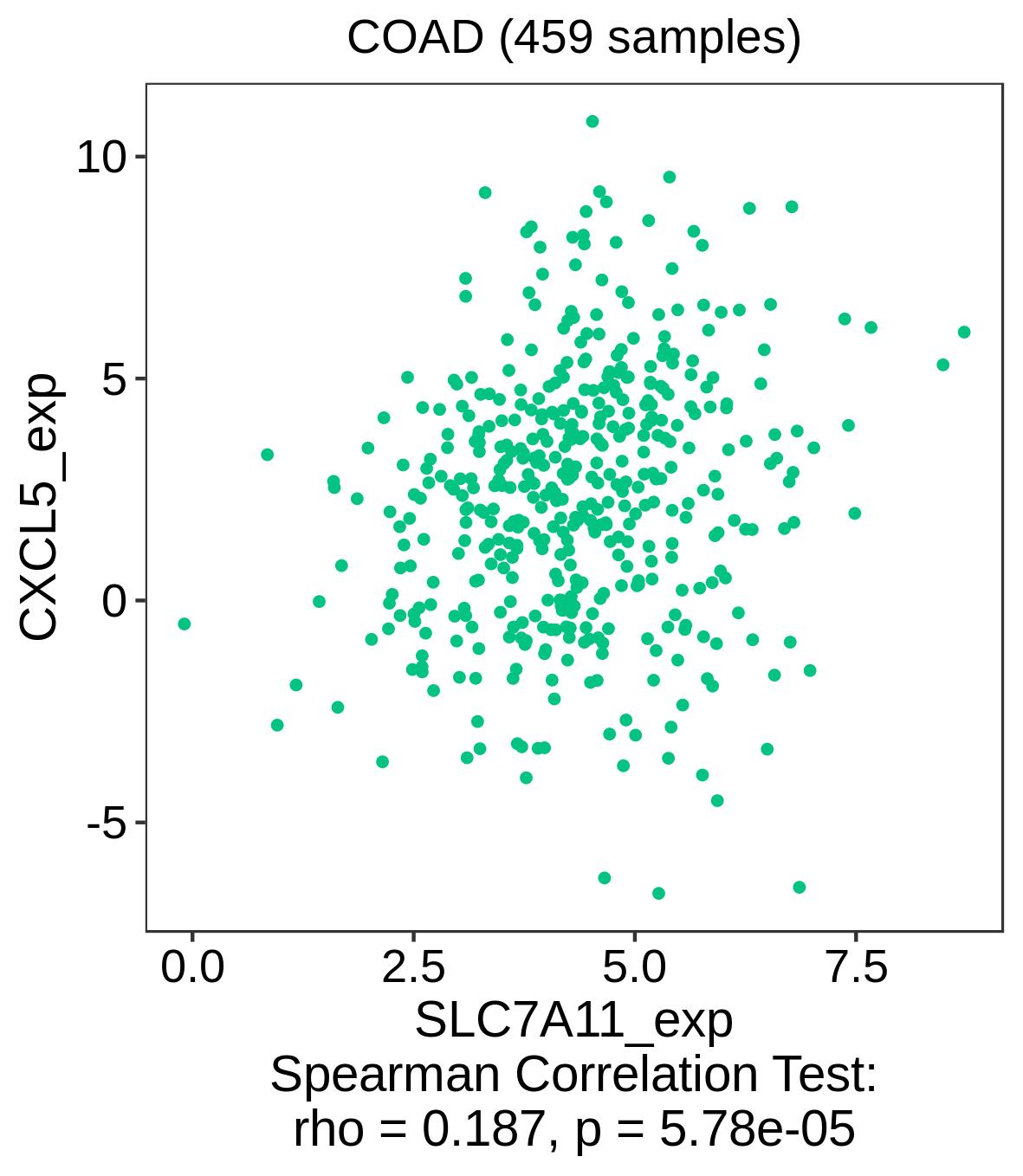

Supplement: Supplementary file 1 [file DataSheet1.ZIP › Raw data/original data/Figure 6 Immune Characteristics/Figure. 6A chemokines/SLC7A11_exp_COAD_chemokine_CXCL5.jpg]

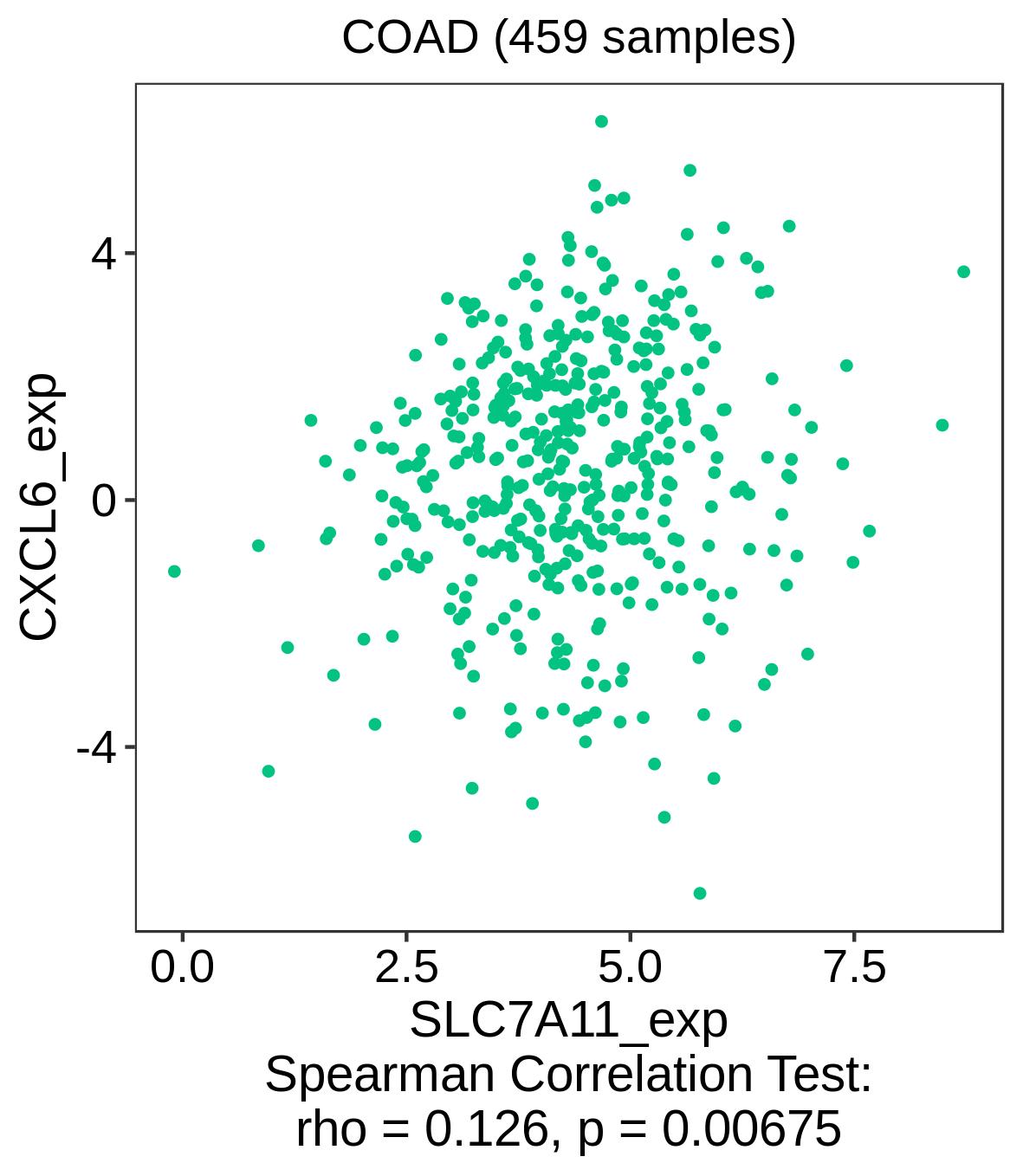

Supplement: Supplementary file 1 [file DataSheet1.ZIP › Raw data/original data/Figure 6 Immune Characteristics/Figure. 6A chemokines/SLC7A11_exp_COAD_chemokine_CXCL6.jpg]

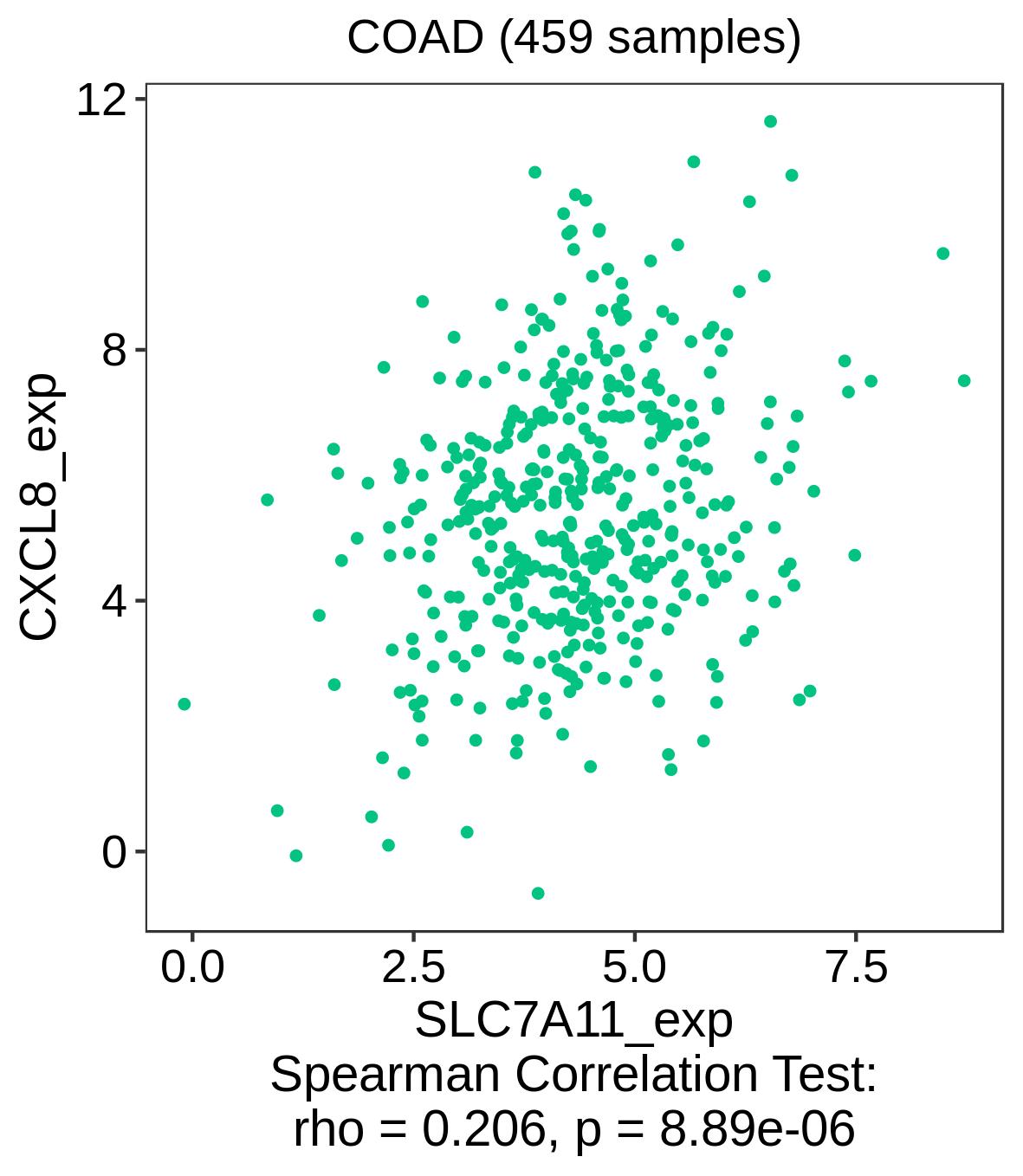

Supplement: Supplementary file 1 [file DataSheet1.ZIP › Raw data/original data/Figure 6 Immune Characteristics/Figure. 6A chemokines/SLC7A11_exp_COAD_chemokine_CXCL8.jpg]

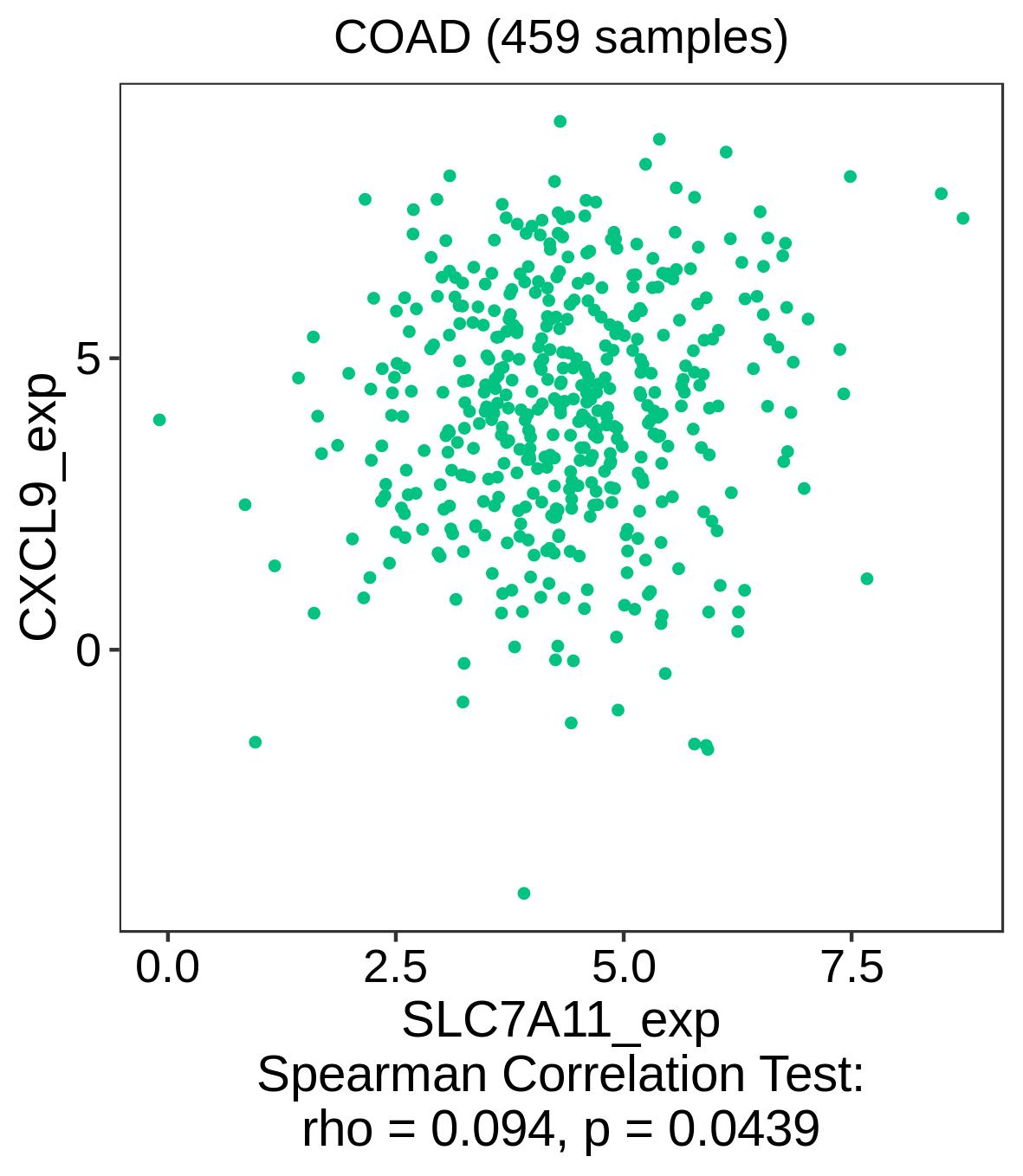

Supplement: Supplementary file 1 [file DataSheet1.ZIP › Raw data/original data/Figure 6 Immune Characteristics/Figure. 6A chemokines/SLC7A11_exp_COAD_chemokine_CXCL9.jpg]

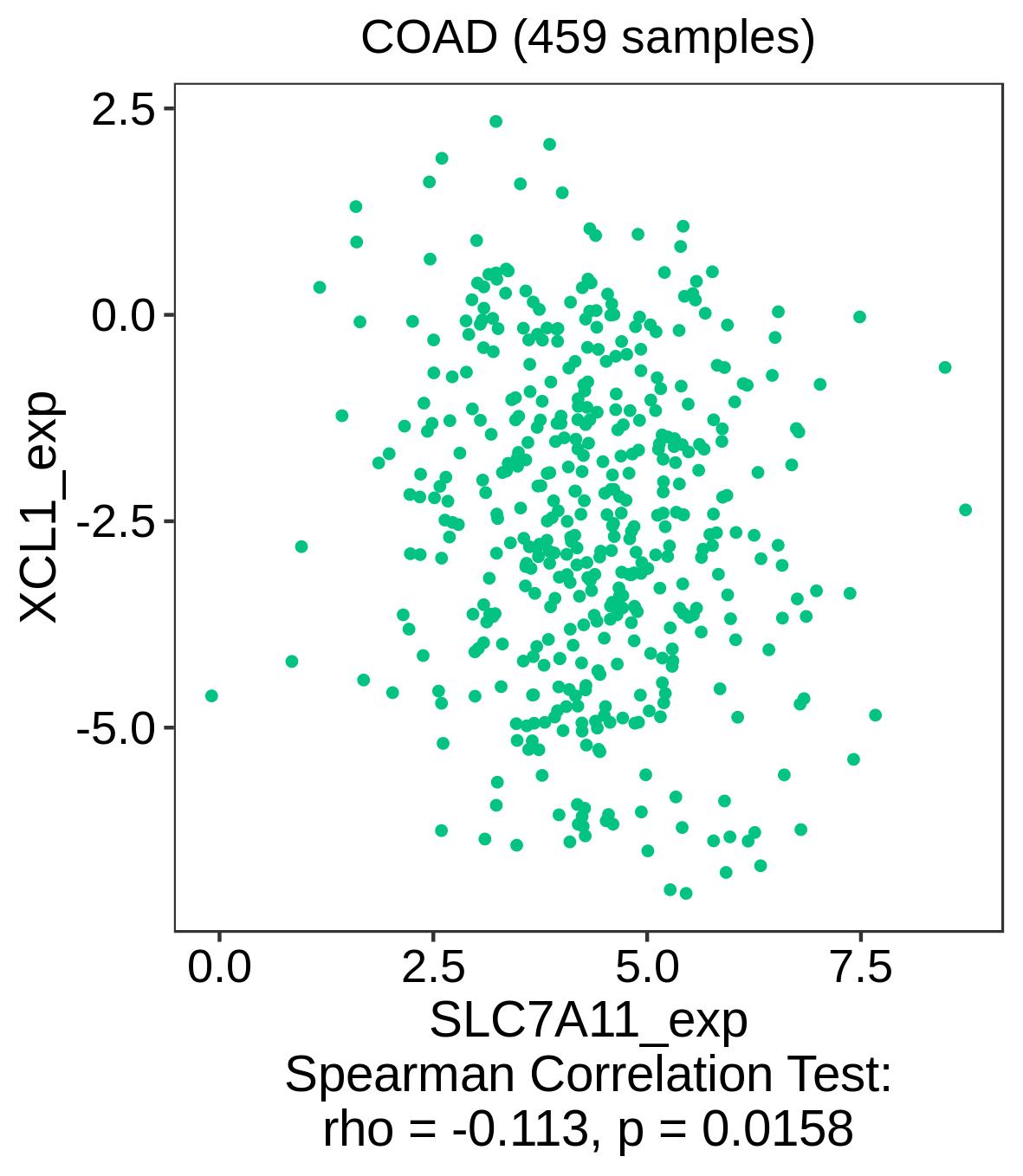

Supplement: Supplementary file 1 [file DataSheet1.ZIP › Raw data/original data/Figure 6 Immune Characteristics/Figure. 6A chemokines/SLC7A11_exp_COAD_chemokine_XCL1.jpg]

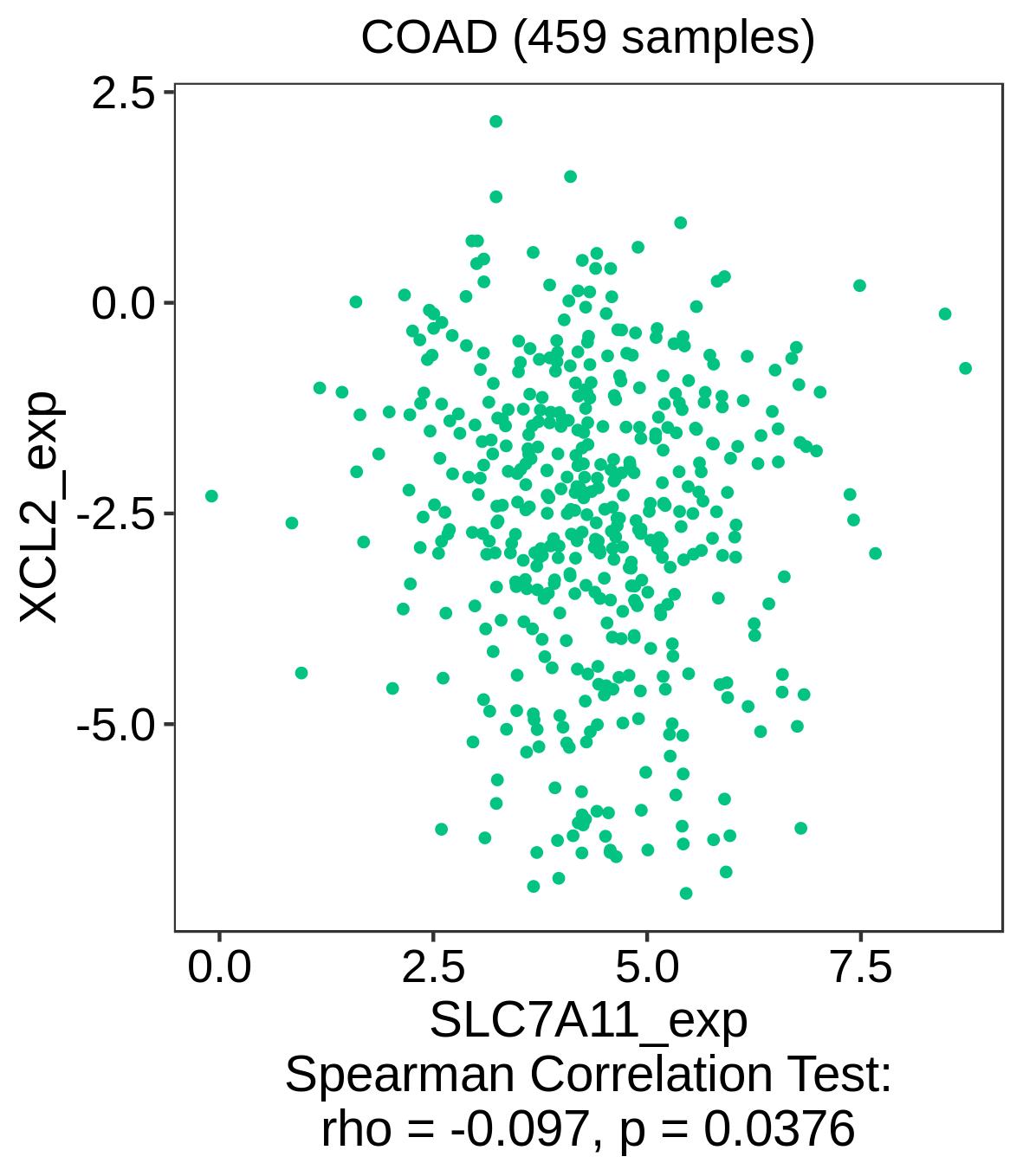

Supplement: Supplementary file 1 [file DataSheet1.ZIP › Raw data/original data/Figure 6 Immune Characteristics/Figure. 6A chemokines/SLC7A11_exp_COAD_chemokine_XCL2.jpg]

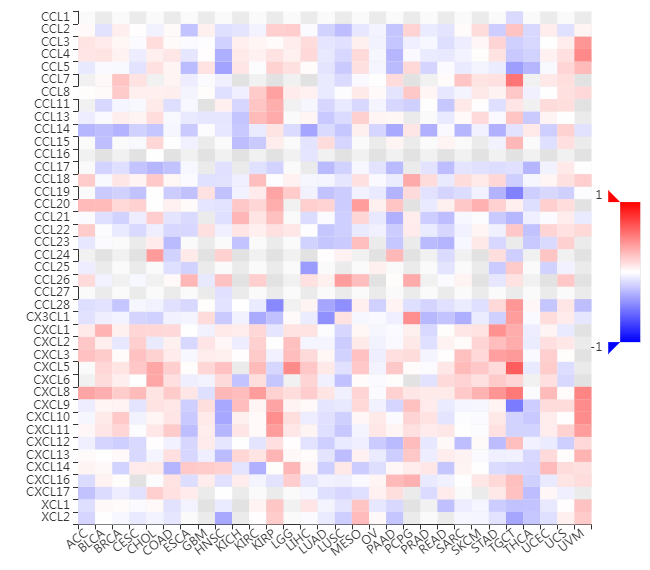

Supplement: Supplementary file 1 [file DataSheet1.ZIP › Raw data/original data/Figure 6 Immune Characteristics/Figure. 6A chemokines/╟≈╗»╥≥╫╙╚╚═╝.png]

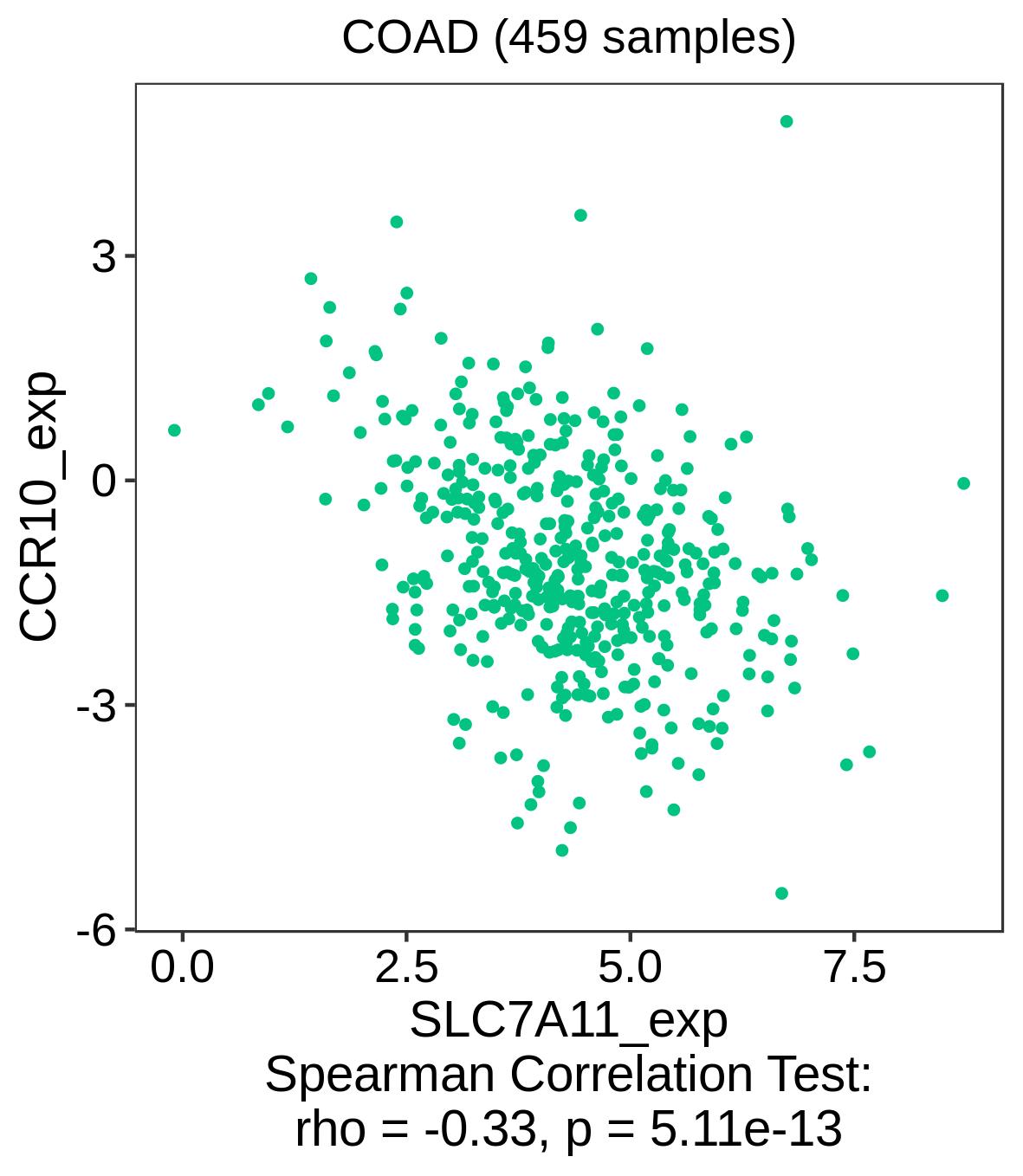

Supplement: Supplementary file 1 [file DataSheet1.ZIP › Raw data/original data/Figure 6 Immune Characteristics/Figure. 6B chemokine receptors/SLC7A11_exp_COAD_receptor_CCR10.jpg]

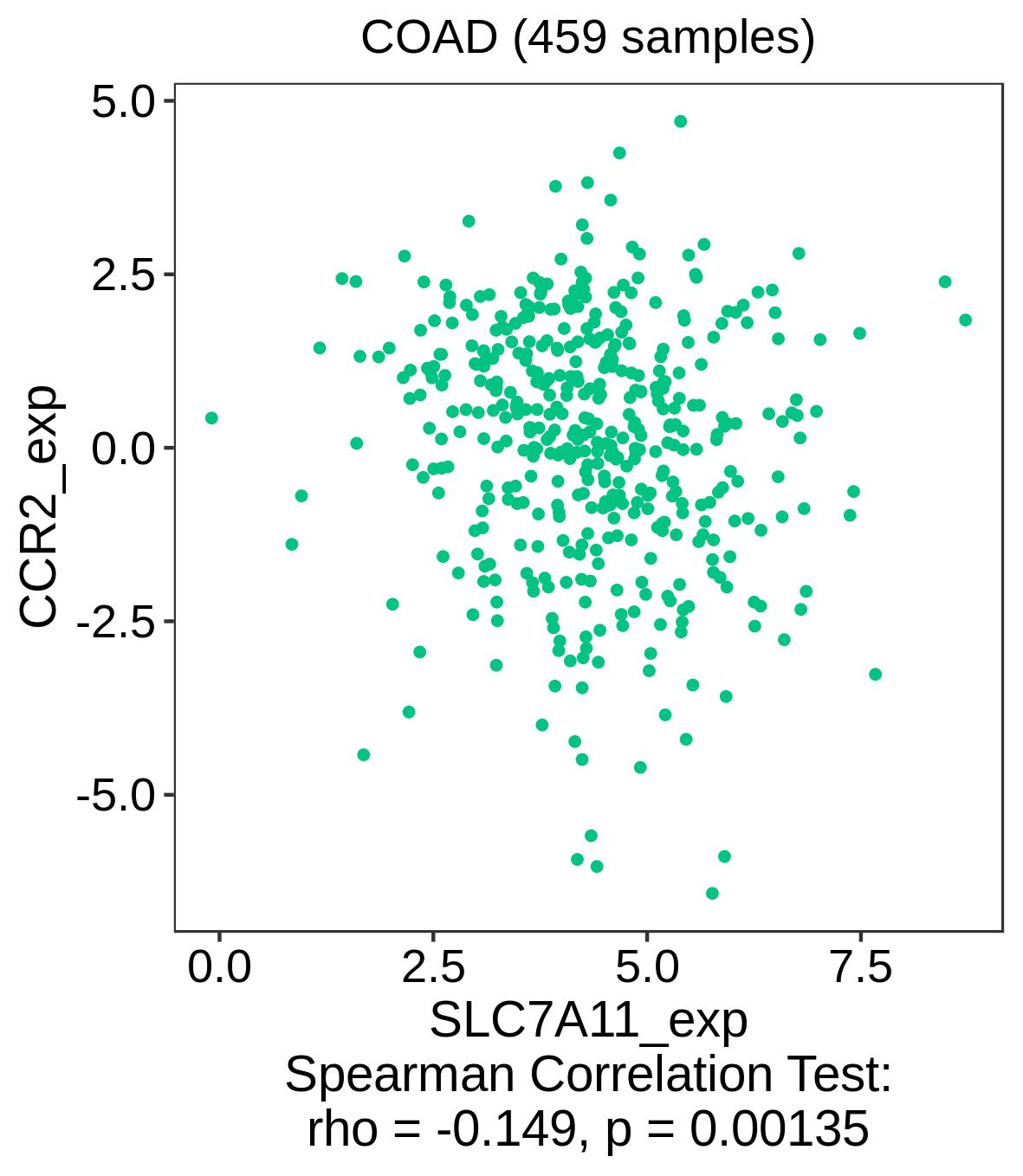

Supplement: Supplementary file 1 [file DataSheet1.ZIP › Raw data/original data/Figure 6 Immune Characteristics/Figure. 6B chemokine receptors/SLC7A11_exp_COAD_receptor_CCR2.jpg]

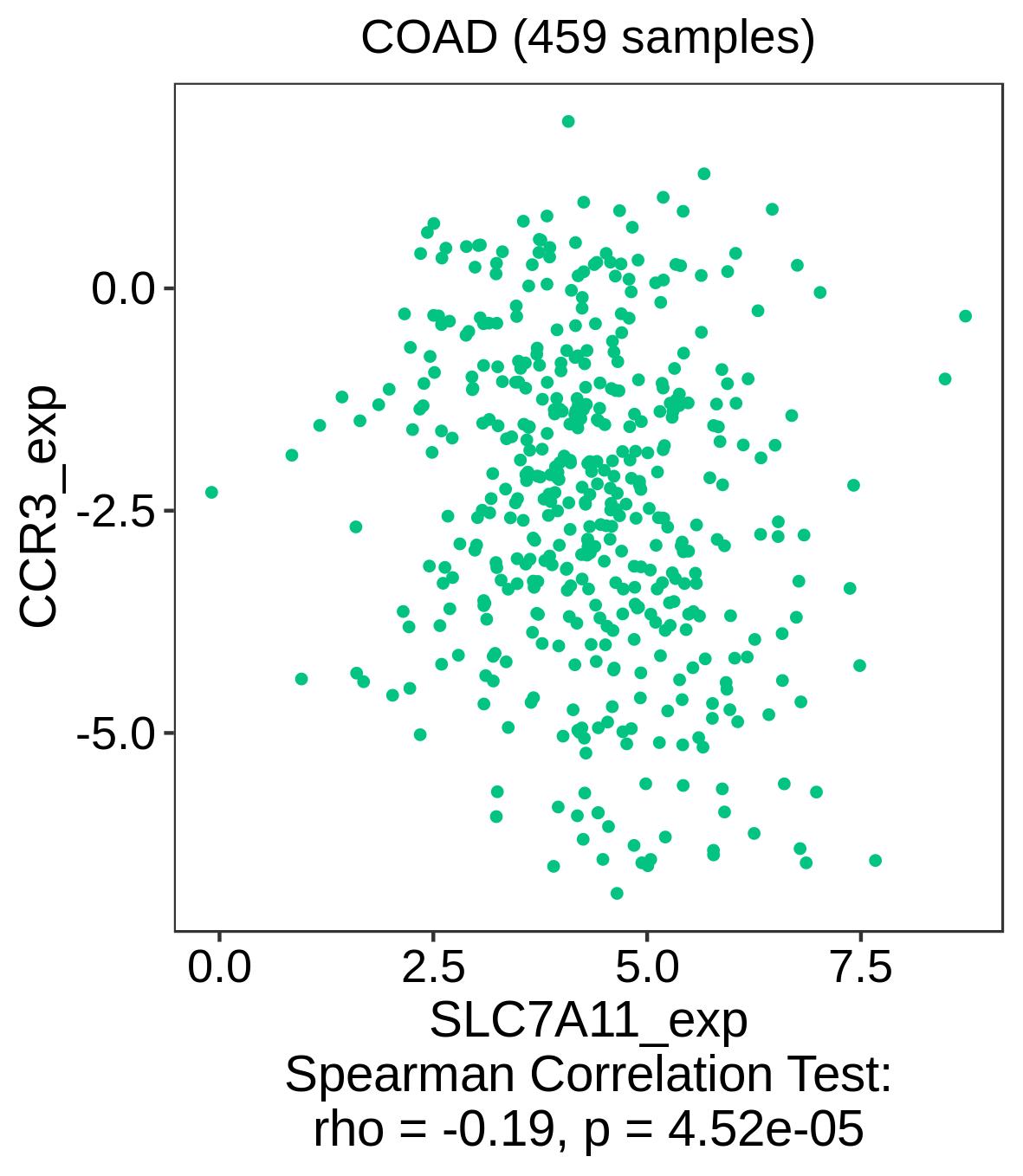

Supplement: Supplementary file 1 [file DataSheet1.ZIP › Raw data/original data/Figure 6 Immune Characteristics/Figure. 6B chemokine receptors/SLC7A11_exp_COAD_receptor_CCR3.jpg]

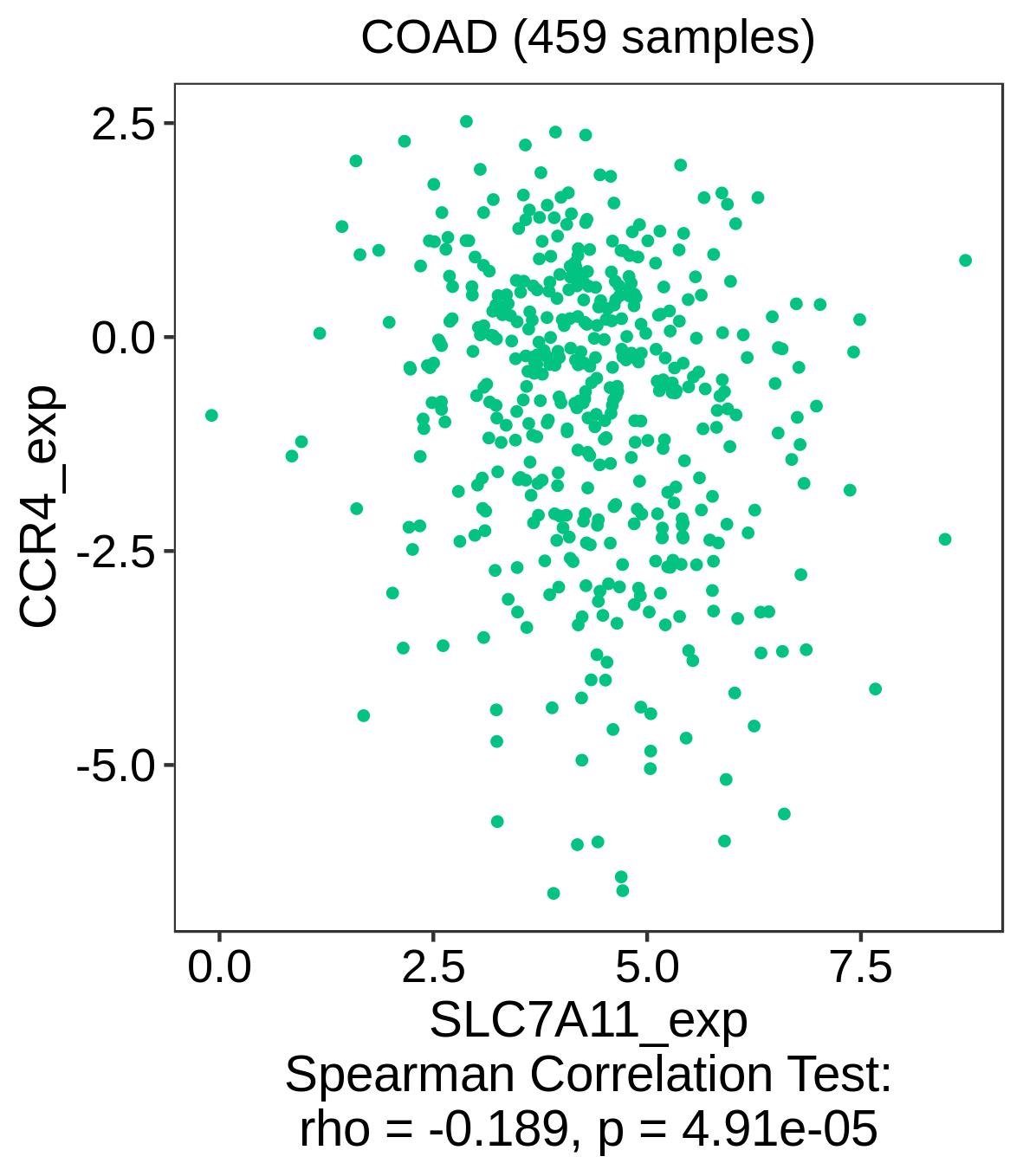

Supplement: Supplementary file 1 [file DataSheet1.ZIP › Raw data/original data/Figure 6 Immune Characteristics/Figure. 6B chemokine receptors/SLC7A11_exp_COAD_receptor_CCR4.jpg]

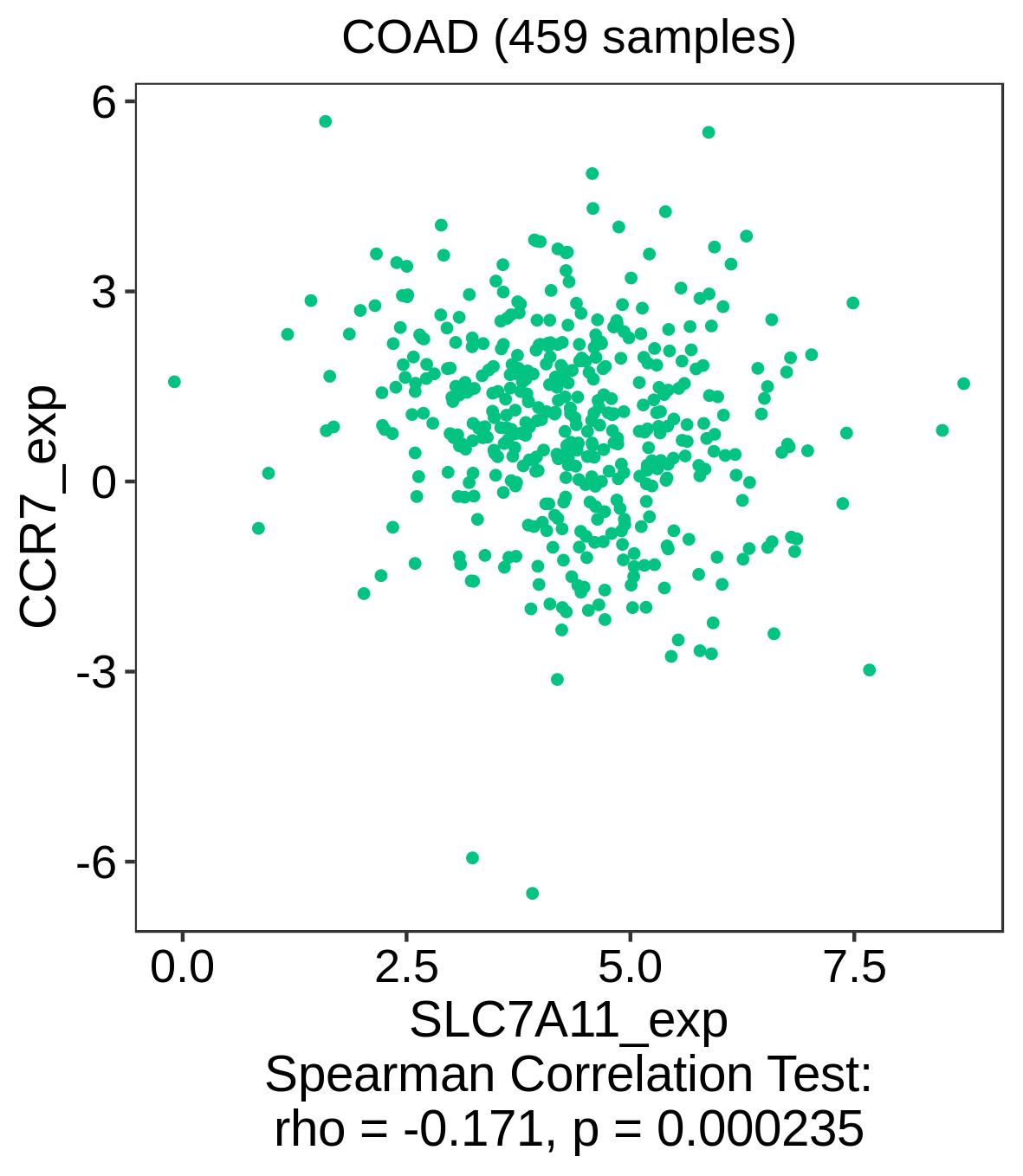

Supplement: Supplementary file 1 [file DataSheet1.ZIP › Raw data/original data/Figure 6 Immune Characteristics/Figure. 6B chemokine receptors/SLC7A11_exp_COAD_receptor_CCR7.jpg]

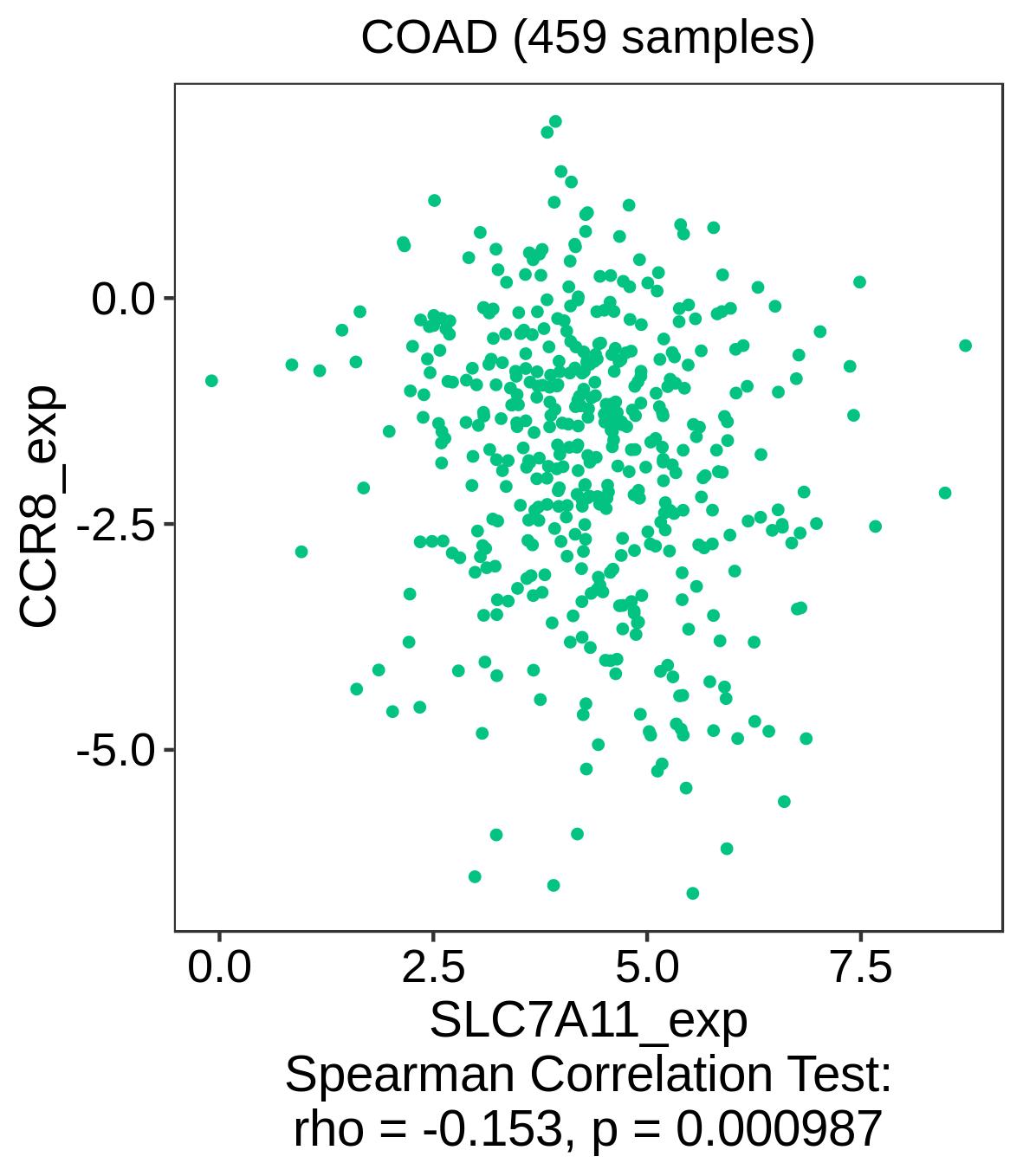

Supplement: Supplementary file 1 [file DataSheet1.ZIP › Raw data/original data/Figure 6 Immune Characteristics/Figure. 6B chemokine receptors/SLC7A11_exp_COAD_receptor_CCR8.jpg]

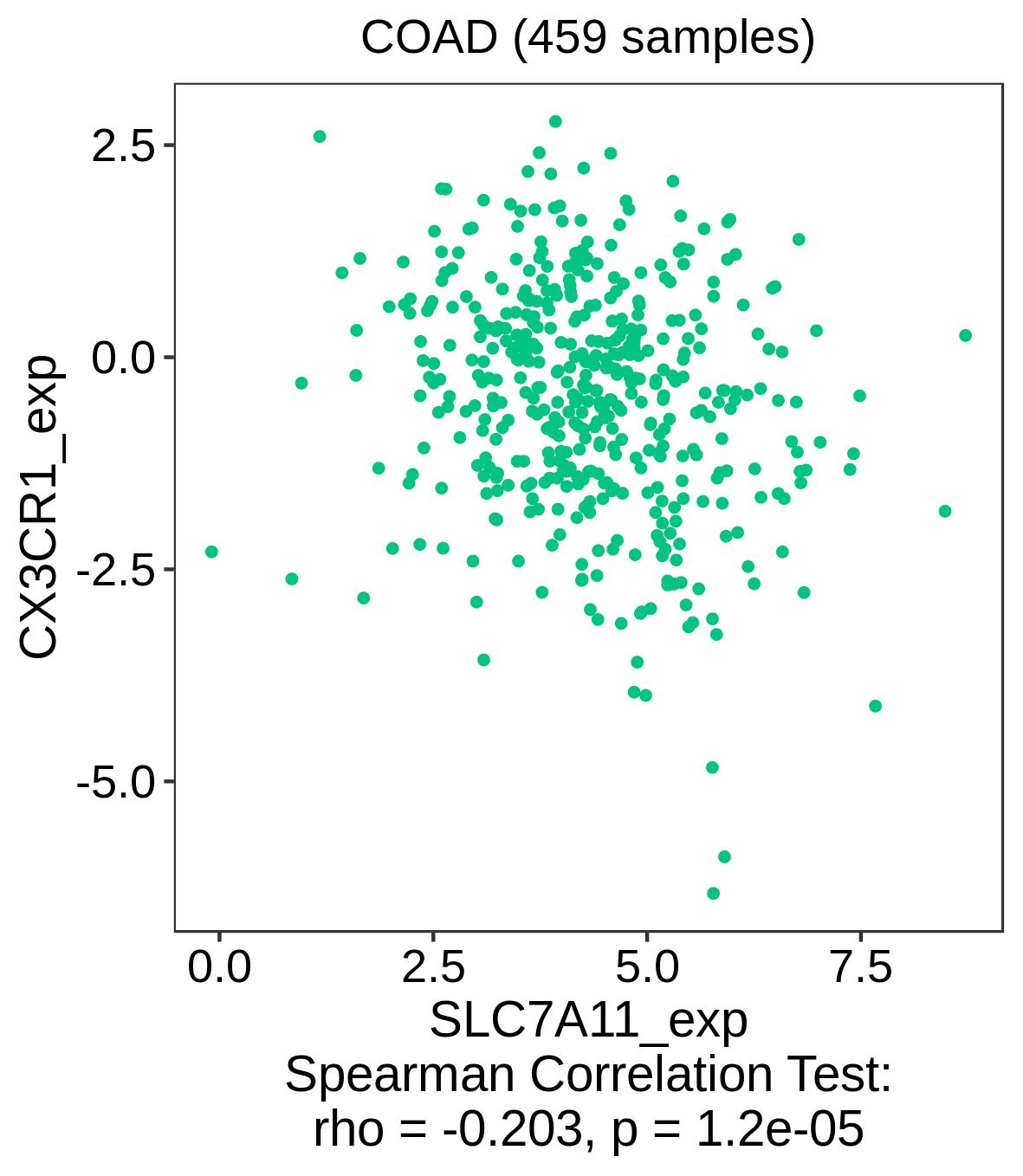

Supplement: Supplementary file 1 [file DataSheet1.ZIP › Raw data/original data/Figure 6 Immune Characteristics/Figure. 6B chemokine receptors/SLC7A11_exp_COAD_receptor_CX3CR1.jpg]

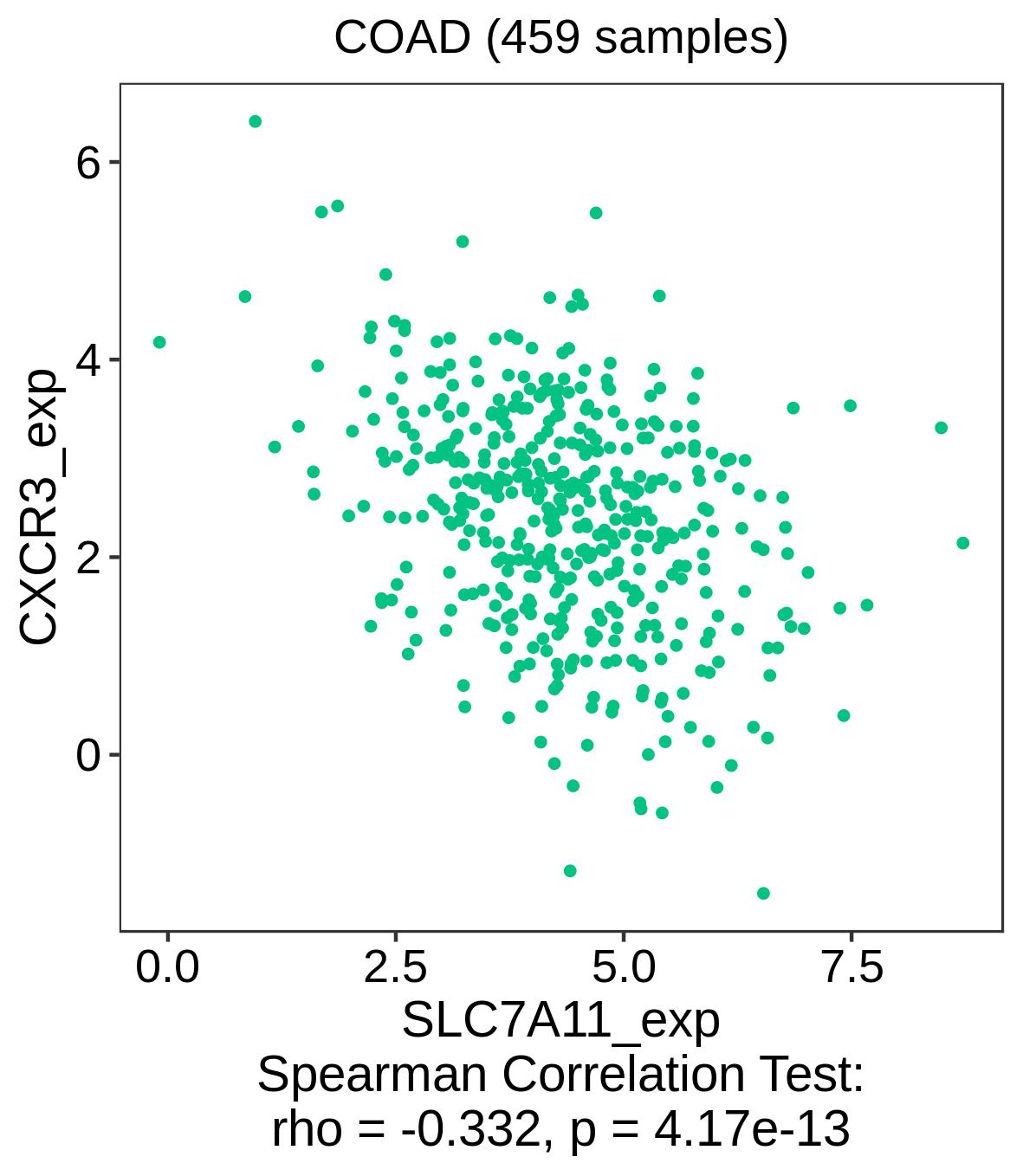

Supplement: Supplementary file 1 [file DataSheet1.ZIP › Raw data/original data/Figure 6 Immune Characteristics/Figure. 6B chemokine receptors/SLC7A11_exp_COAD_receptor_CXCR3.jpg]

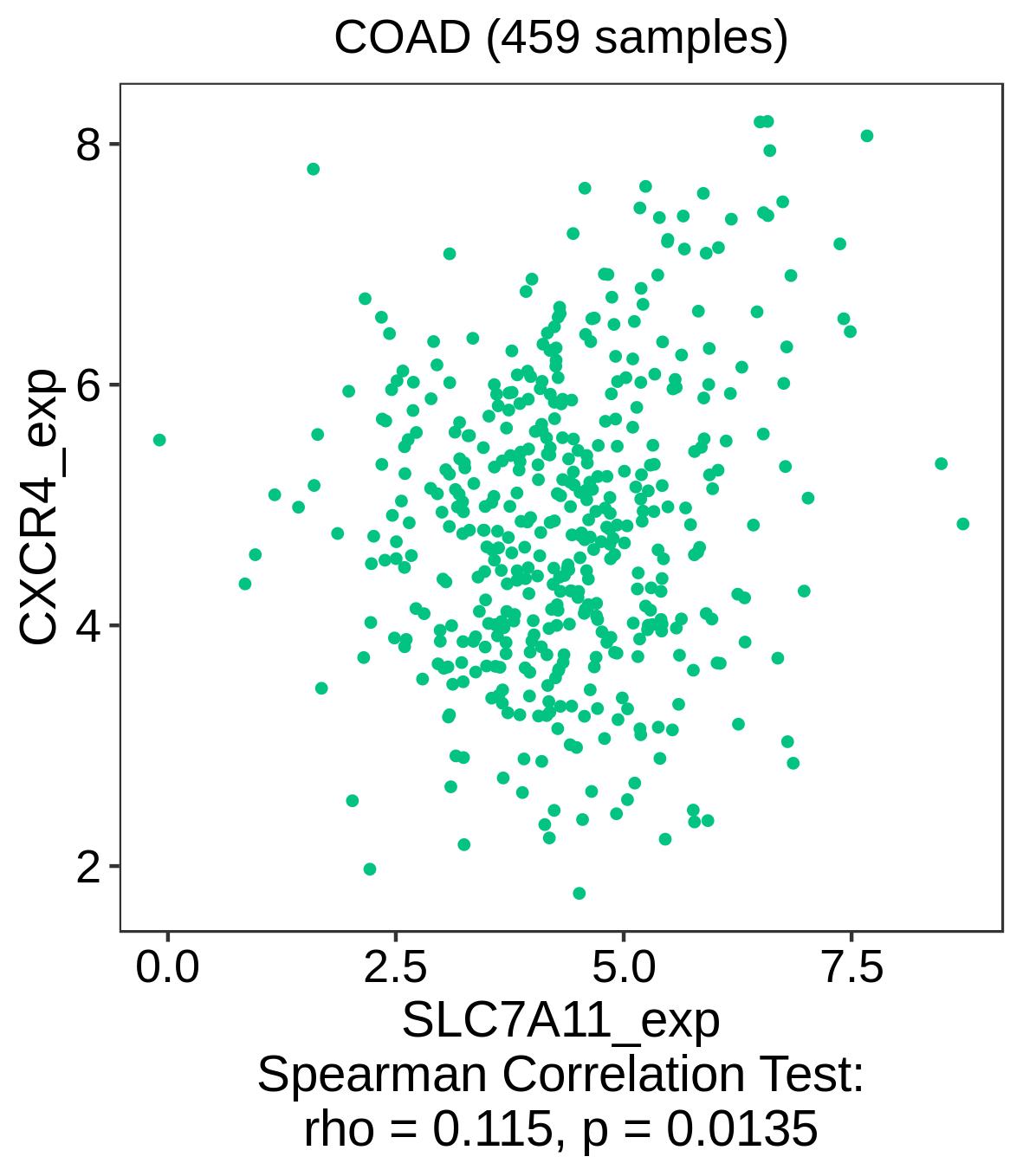

Supplement: Supplementary file 1 [file DataSheet1.ZIP › Raw data/original data/Figure 6 Immune Characteristics/Figure. 6B chemokine receptors/SLC7A11_exp_COAD_receptor_CXCR4.jpg]

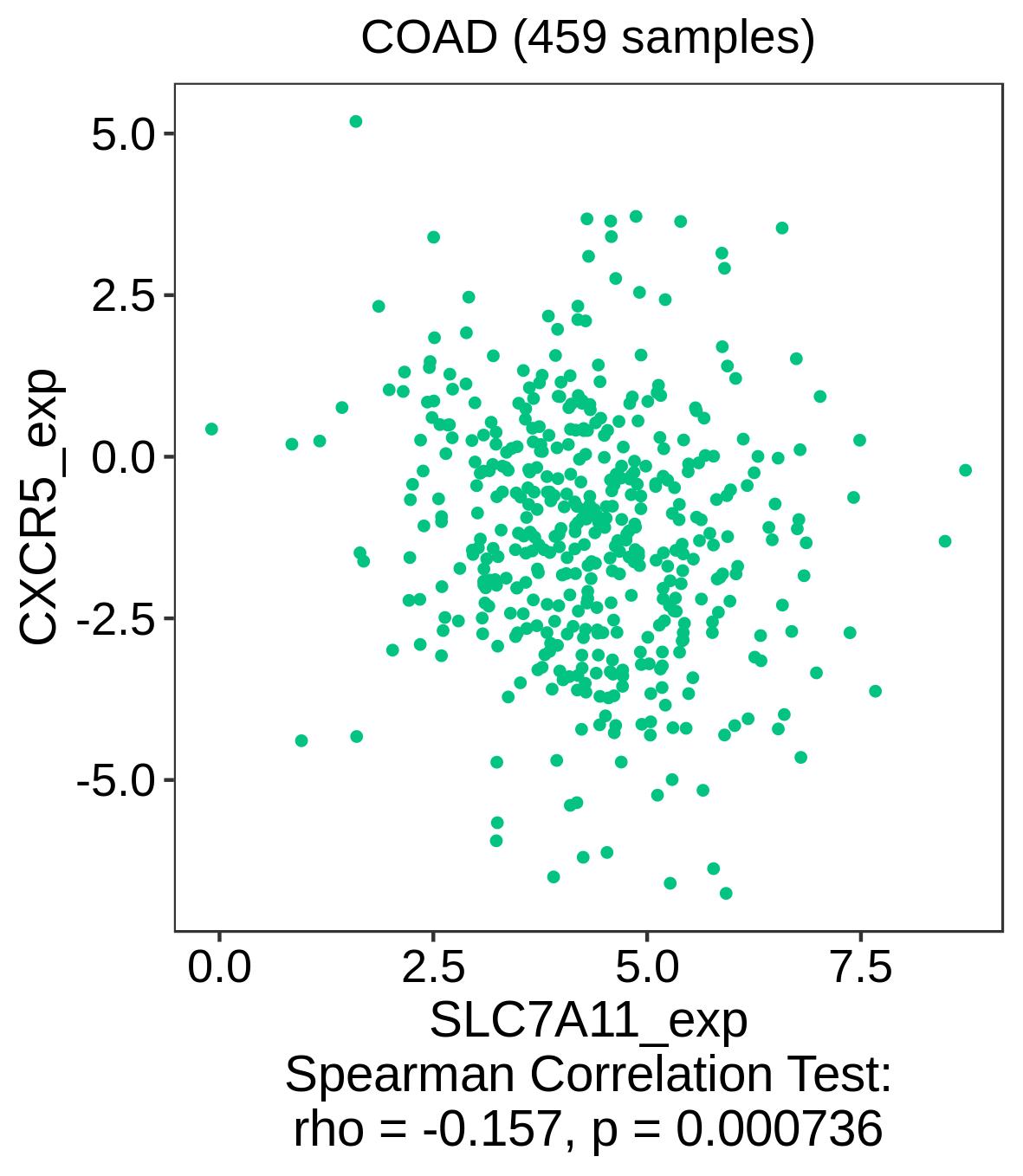

Supplement: Supplementary file 1 [file DataSheet1.ZIP › Raw data/original data/Figure 6 Immune Characteristics/Figure. 6B chemokine receptors/SLC7A11_exp_COAD_receptor_CXCR5.jpg]

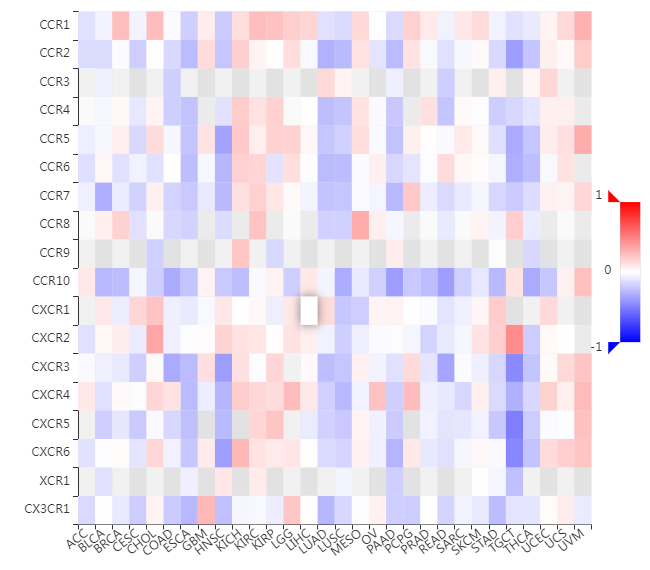

Supplement: Supplementary file 1 [file DataSheet1.ZIP › Raw data/original data/Figure 6 Immune Characteristics/Figure. 6B chemokine receptors/╧┬╘╪.png]

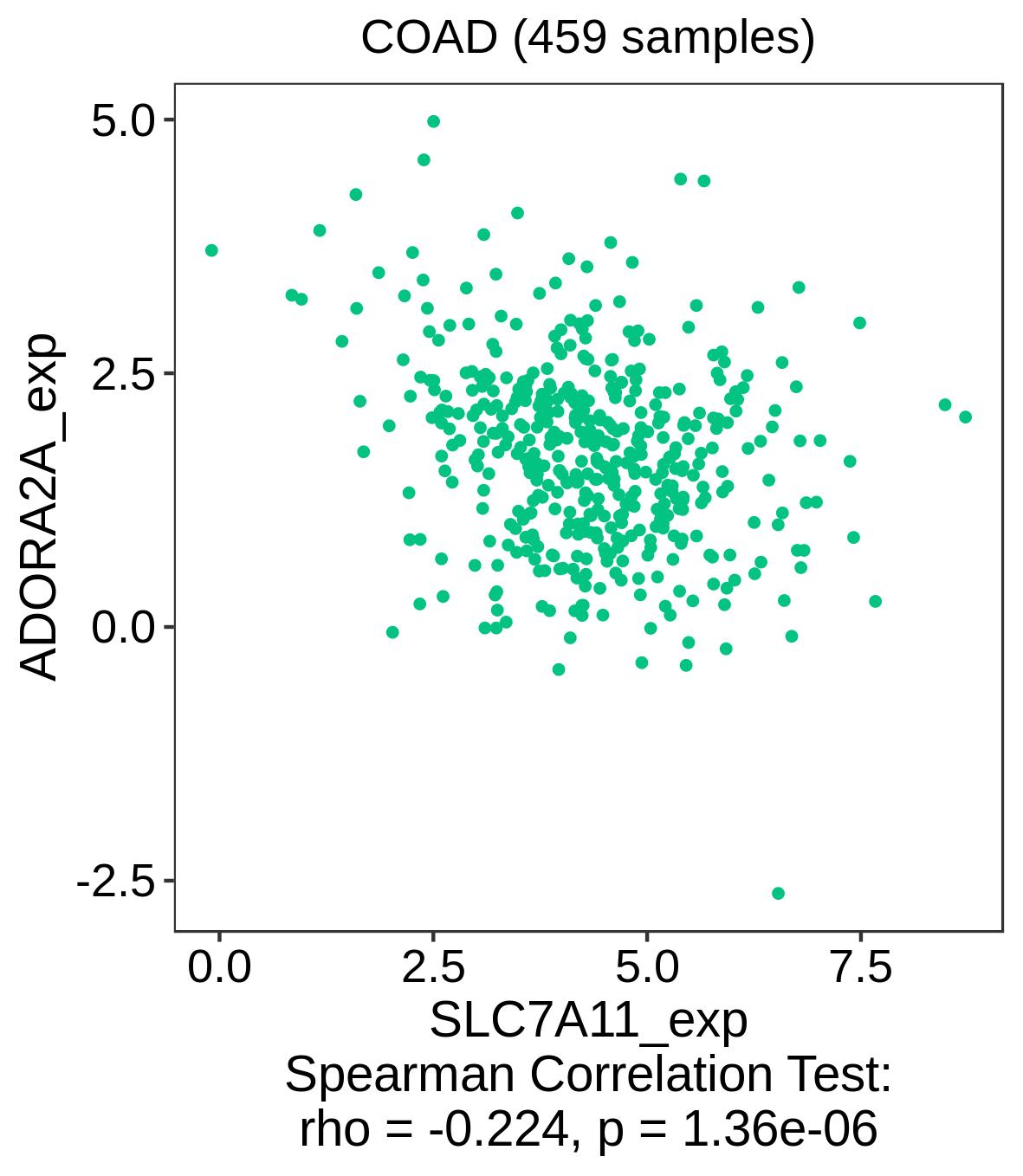

Supplement: Supplementary file 1 [file DataSheet1.ZIP › Raw data/original data/Figure 6 Immune Characteristics/Figure. 6C immunoinhibitors/SLC7A11_exp_COAD_Immunoinhibitor_ADORA2A.jpg]

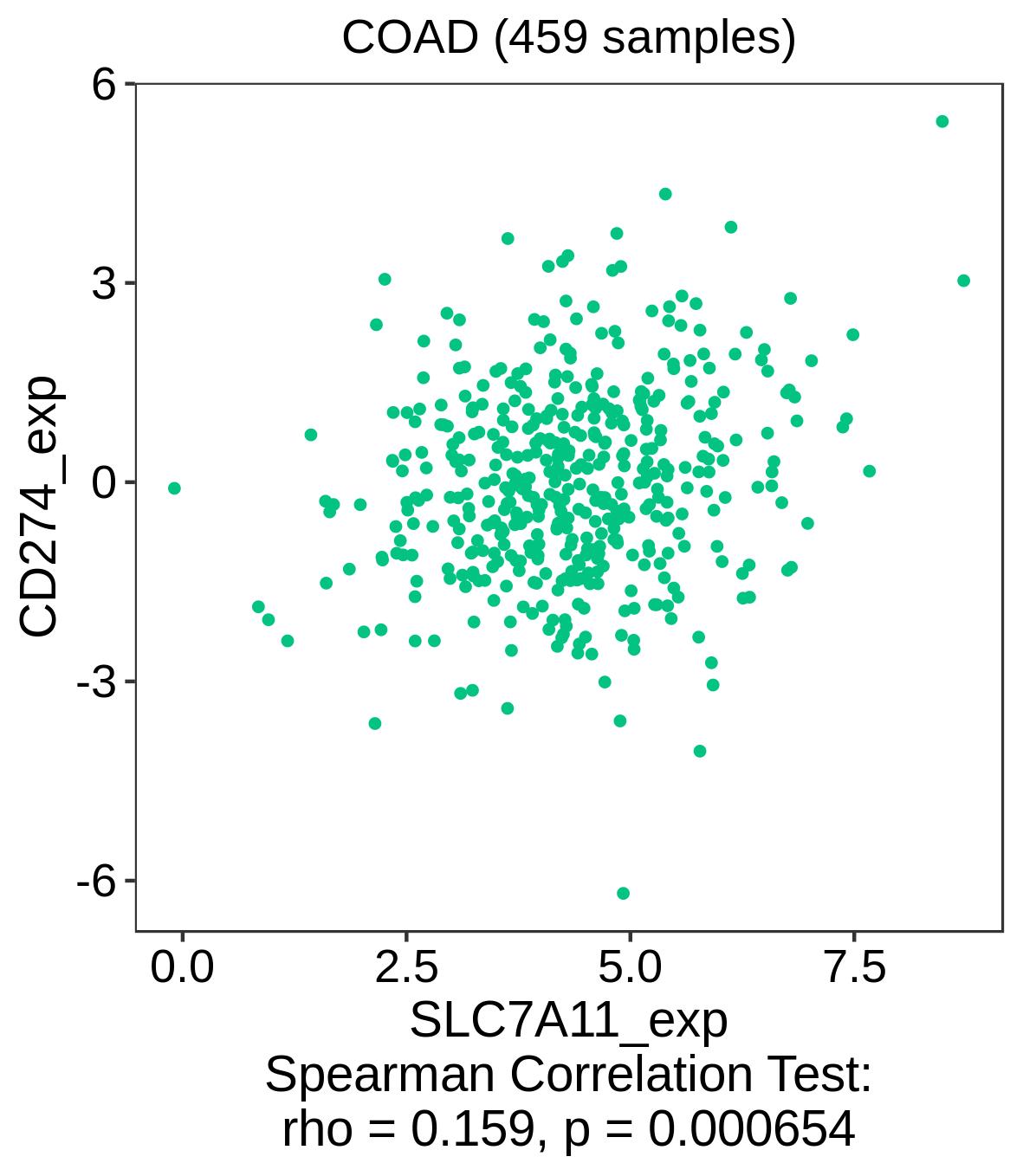

Supplement: Supplementary file 1 [file DataSheet1.ZIP › Raw data/original data/Figure 6 Immune Characteristics/Figure. 6C immunoinhibitors/SLC7A11_exp_COAD_Immunoinhibitor_CD274.jpg]

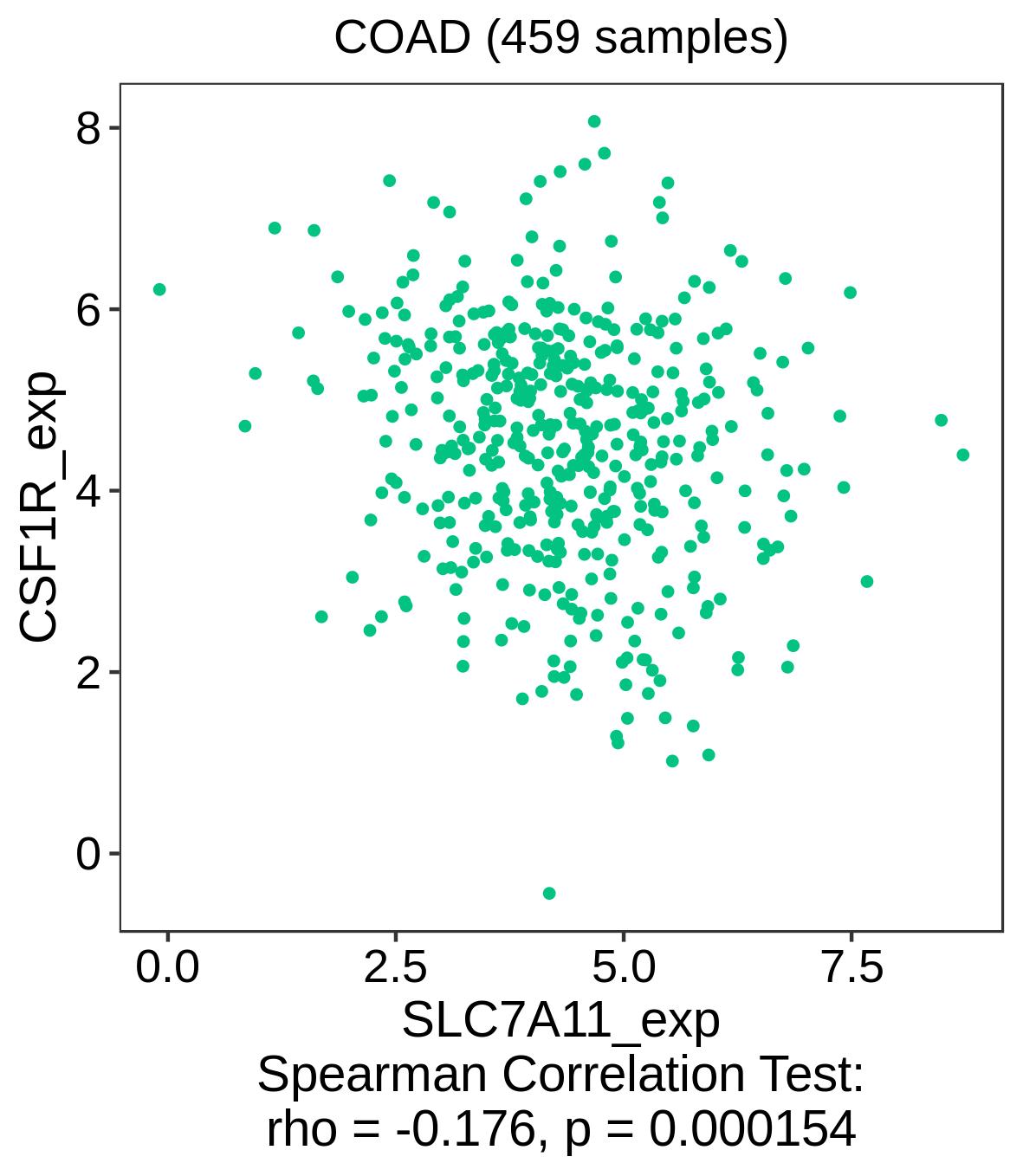

Supplement: Supplementary file 1 [file DataSheet1.ZIP › Raw data/original data/Figure 6 Immune Characteristics/Figure. 6C immunoinhibitors/SLC7A11_exp_COAD_Immunoinhibitor_CSF1R.jpg]

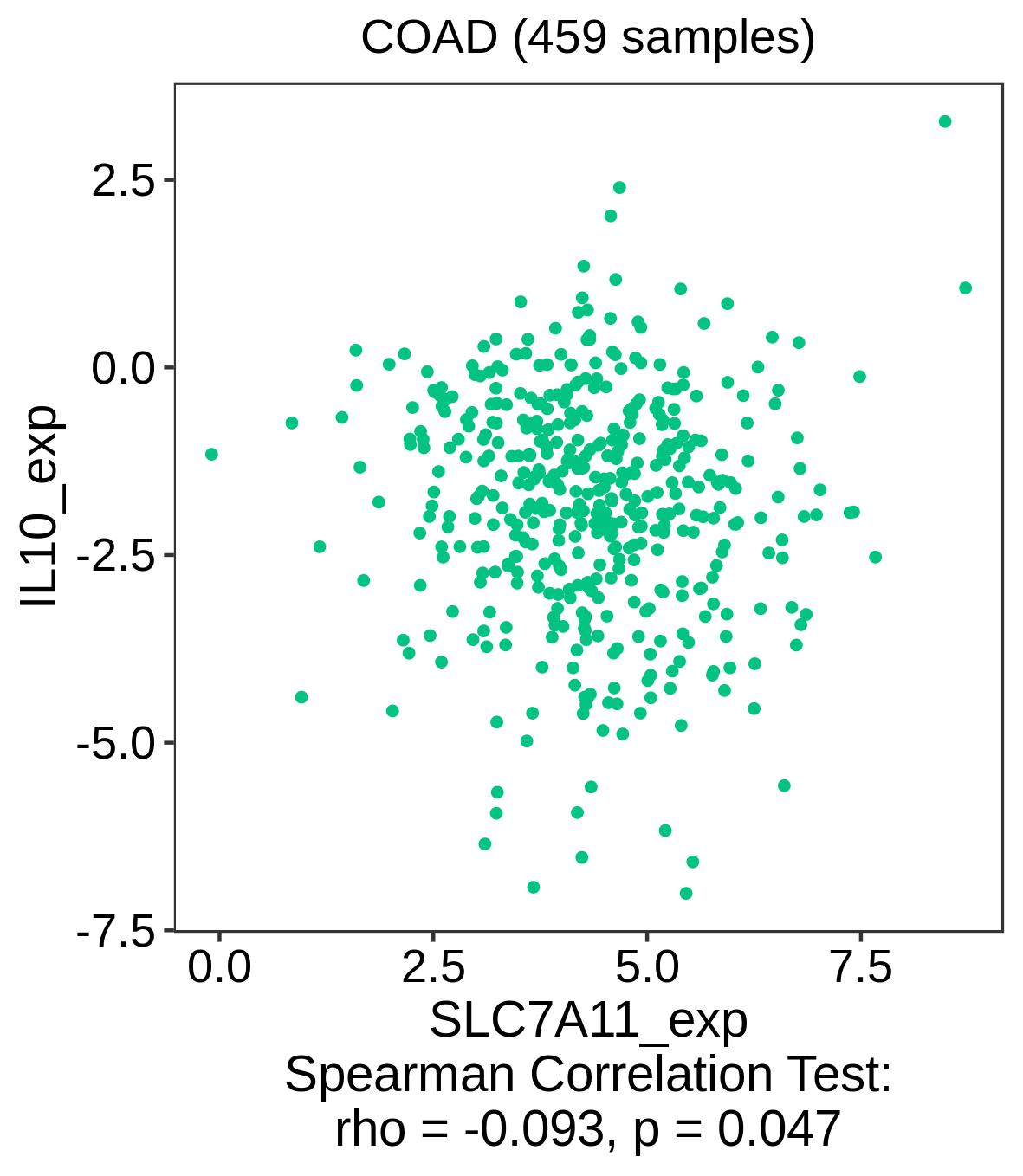

Supplement: Supplementary file 1 [file DataSheet1.ZIP › Raw data/original data/Figure 6 Immune Characteristics/Figure. 6C immunoinhibitors/SLC7A11_exp_COAD_Immunoinhibitor_IL10.jpg]

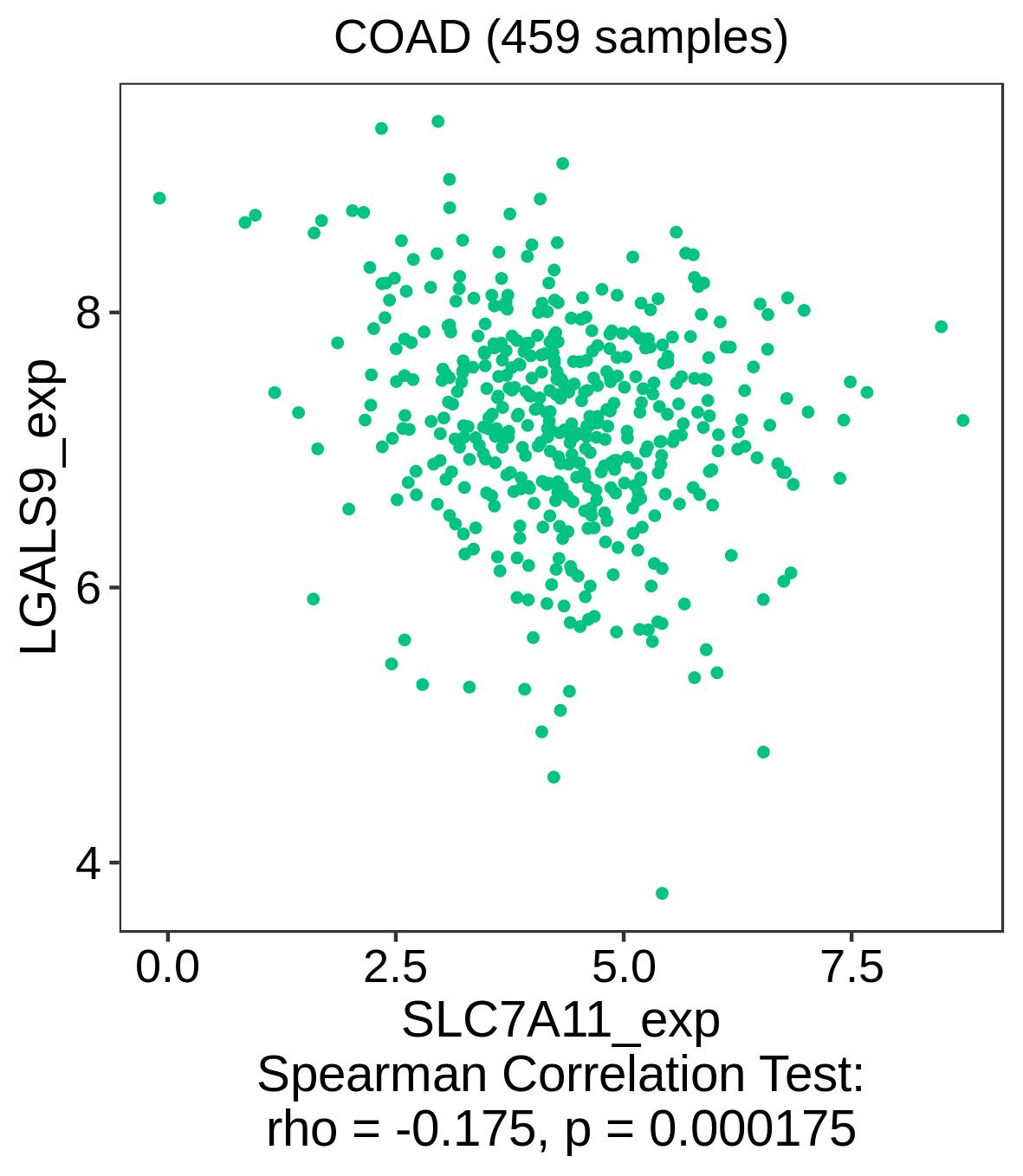

Supplement: Supplementary file 1 [file DataSheet1.ZIP › Raw data/original data/Figure 6 Immune Characteristics/Figure. 6C immunoinhibitors/SLC7A11_exp_COAD_Immunoinhibitor_LGALS9.jpg]

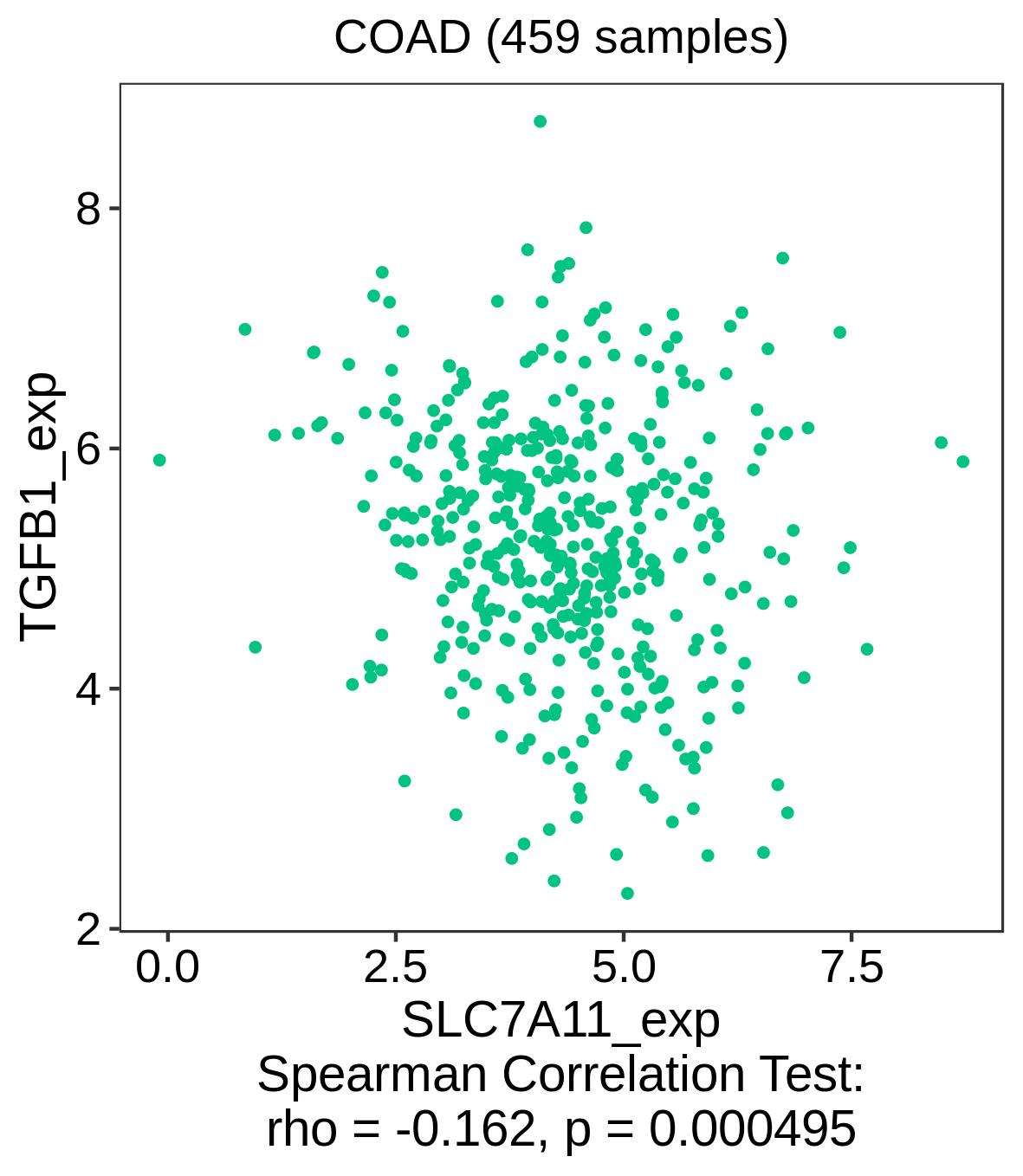

Supplement: Supplementary file 1 [file DataSheet1.ZIP › Raw data/original data/Figure 6 Immune Characteristics/Figure. 6C immunoinhibitors/SLC7A11_exp_COAD_Immunoinhibitor_TGFB1.jpg]

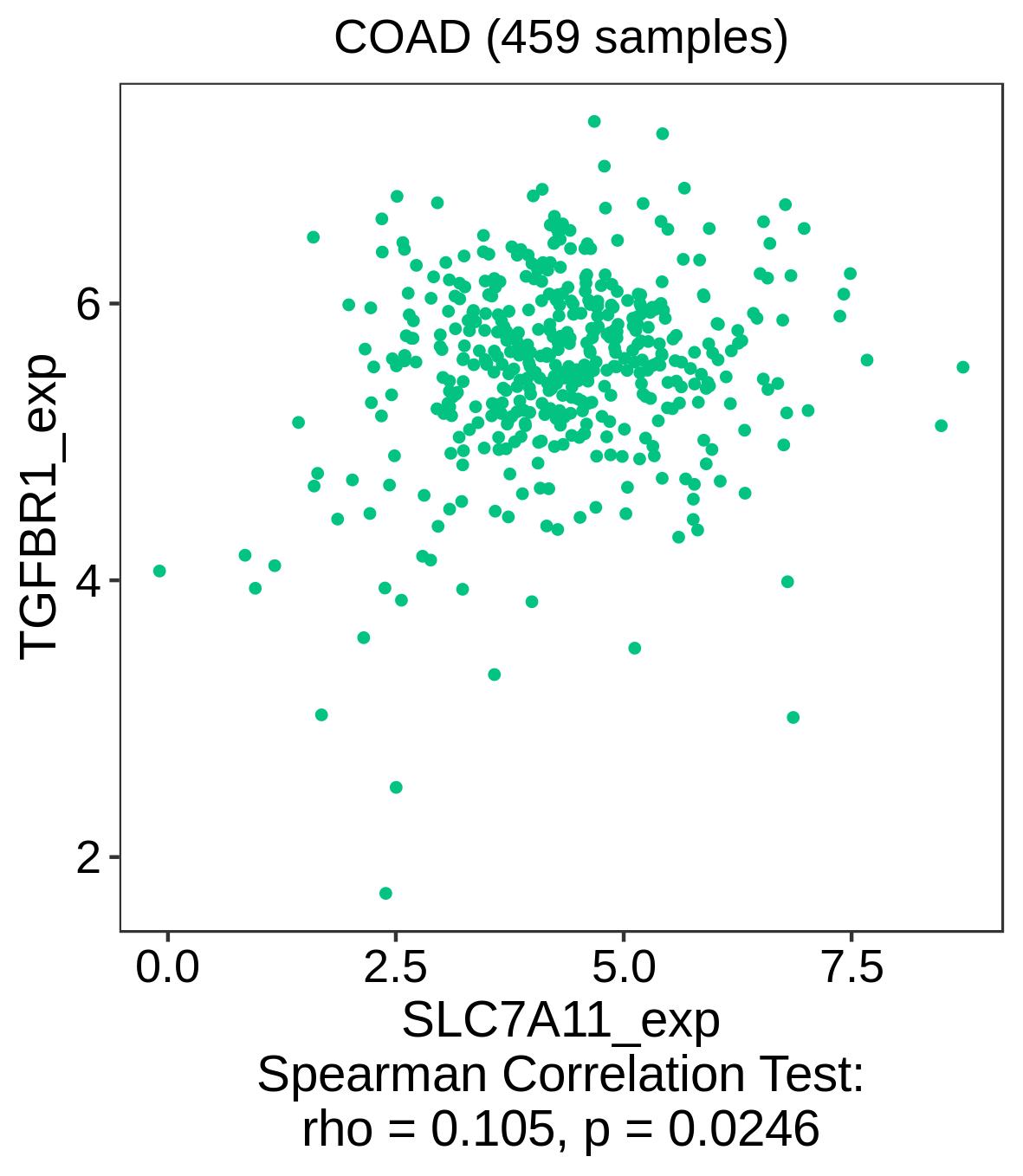

Supplement: Supplementary file 1 [file DataSheet1.ZIP › Raw data/original data/Figure 6 Immune Characteristics/Figure. 6C immunoinhibitors/SLC7A11_exp_COAD_Immunoinhibitor_TGFBR1.jpg]

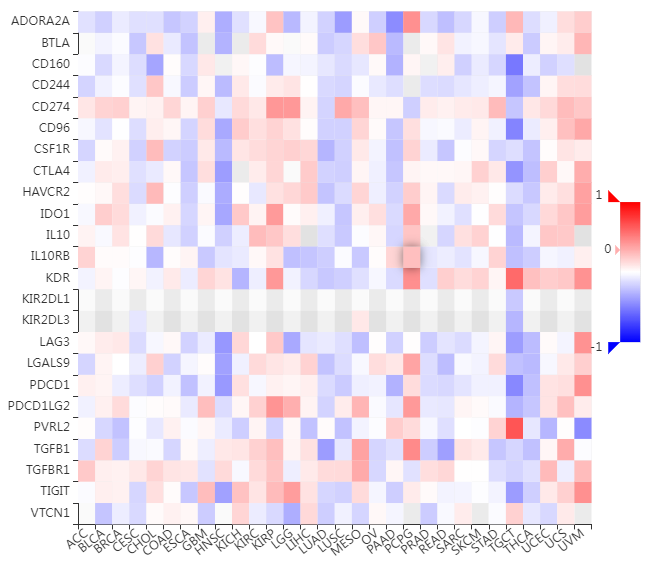

Supplement: Supplementary file 1 [file DataSheet1.ZIP › Raw data/original data/Figure 6 Immune Characteristics/Figure. 6C immunoinhibitors/╧┬╘╪ (1).png]

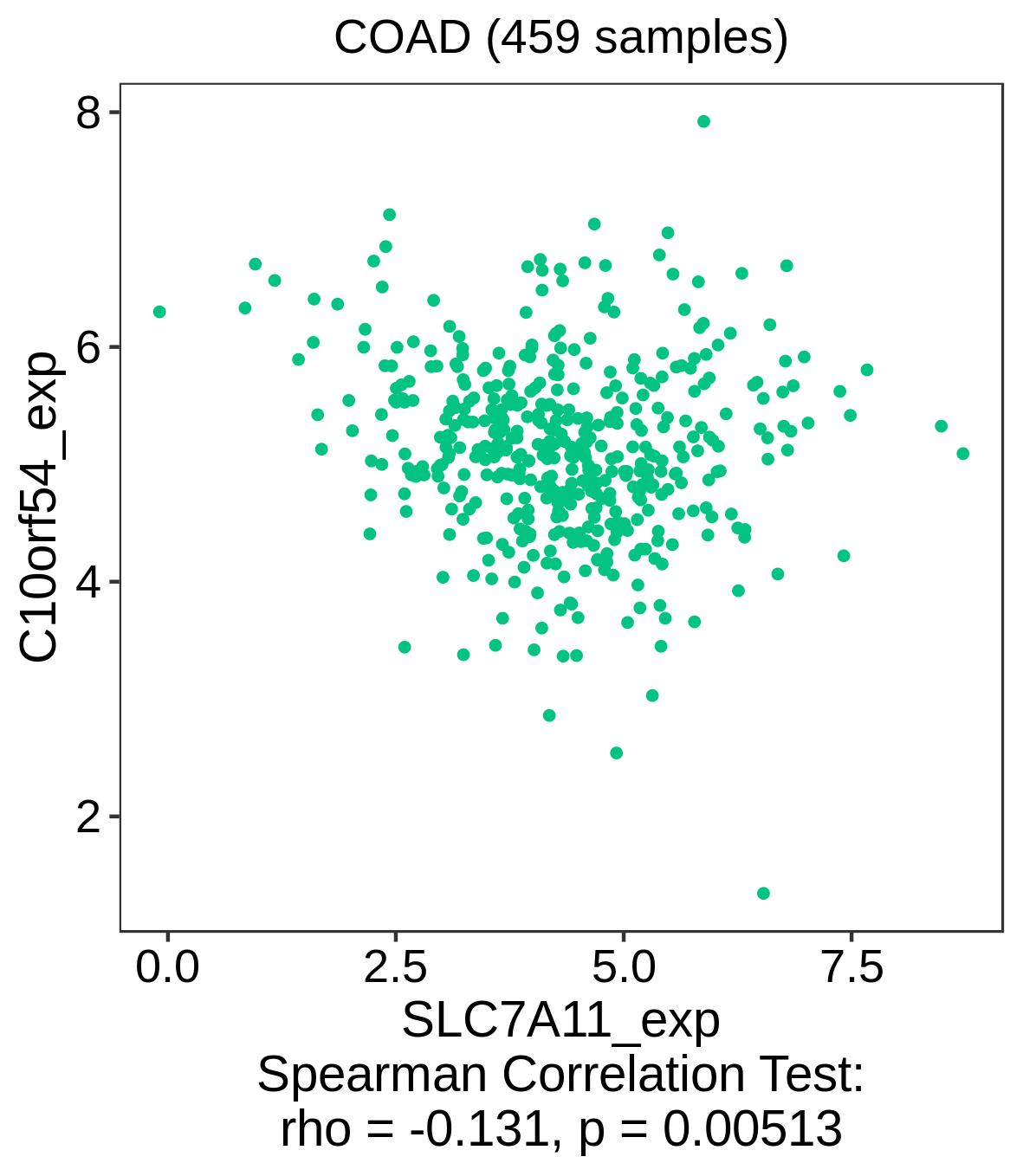

Supplement: Supplementary file 1 [file DataSheet1.ZIP › Raw data/original data/Figure 6 Immune Characteristics/Figure. 6D immunostimulators/SLC7A11_exp_COAD_Immunostimulator_C10orf54.jpg]

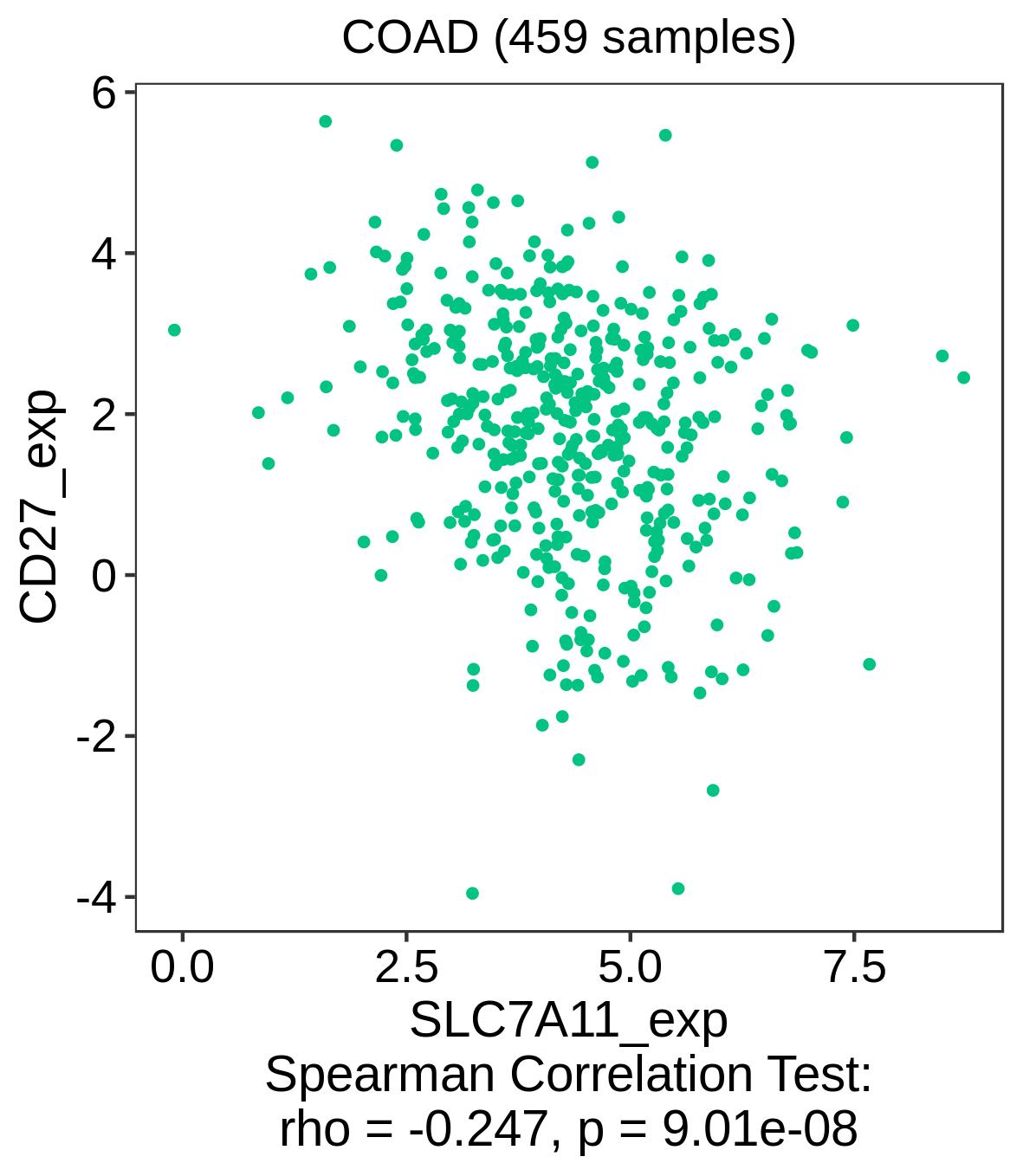

Supplement: Supplementary file 1 [file DataSheet1.ZIP › Raw data/original data/Figure 6 Immune Characteristics/Figure. 6D immunostimulators/SLC7A11_exp_COAD_Immunostimulator_CD27.jpg]

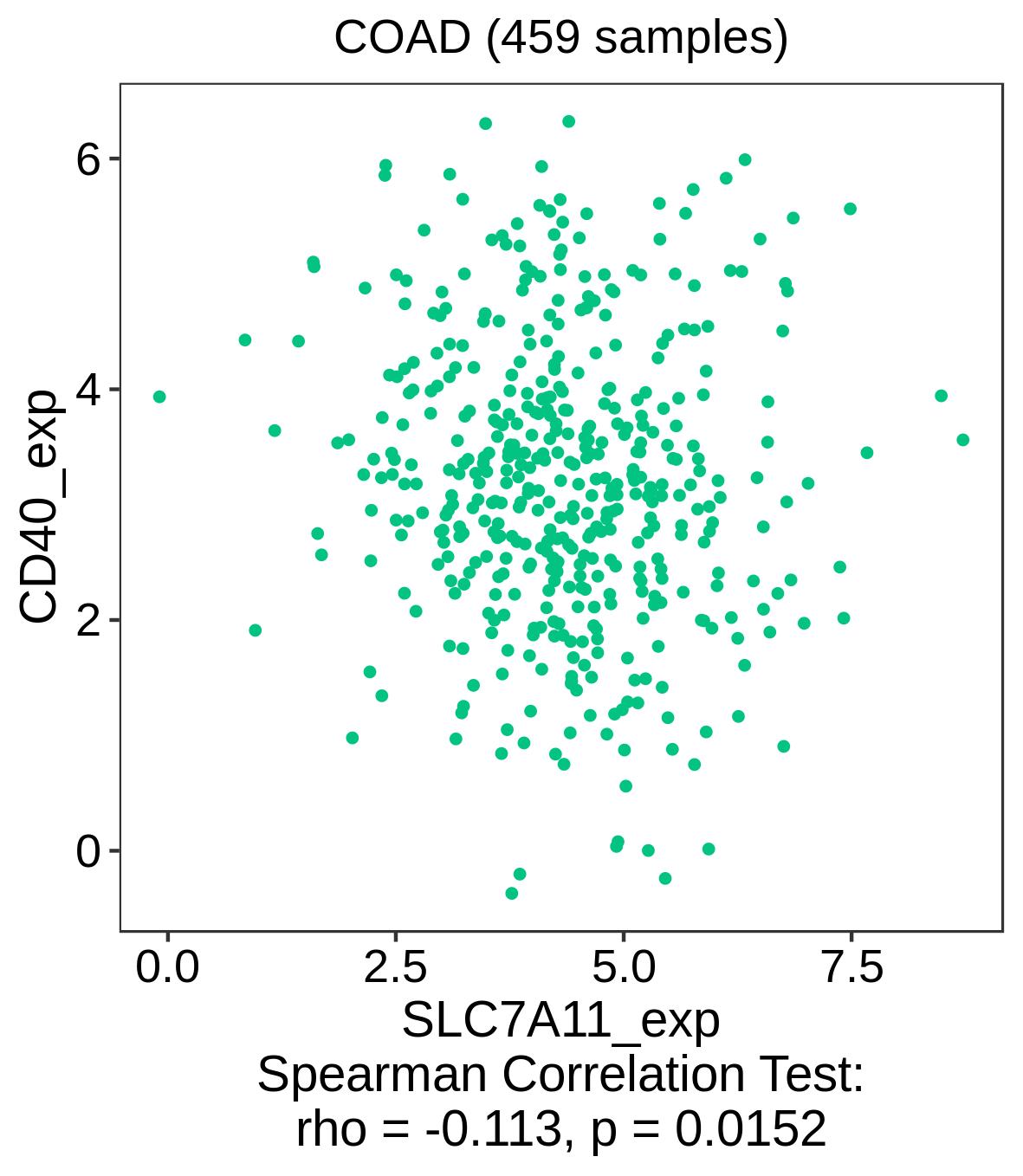

Supplement: Supplementary file 1 [file DataSheet1.ZIP › Raw data/original data/Figure 6 Immune Characteristics/Figure. 6D immunostimulators/SLC7A11_exp_COAD_Immunostimulator_CD40.jpg]

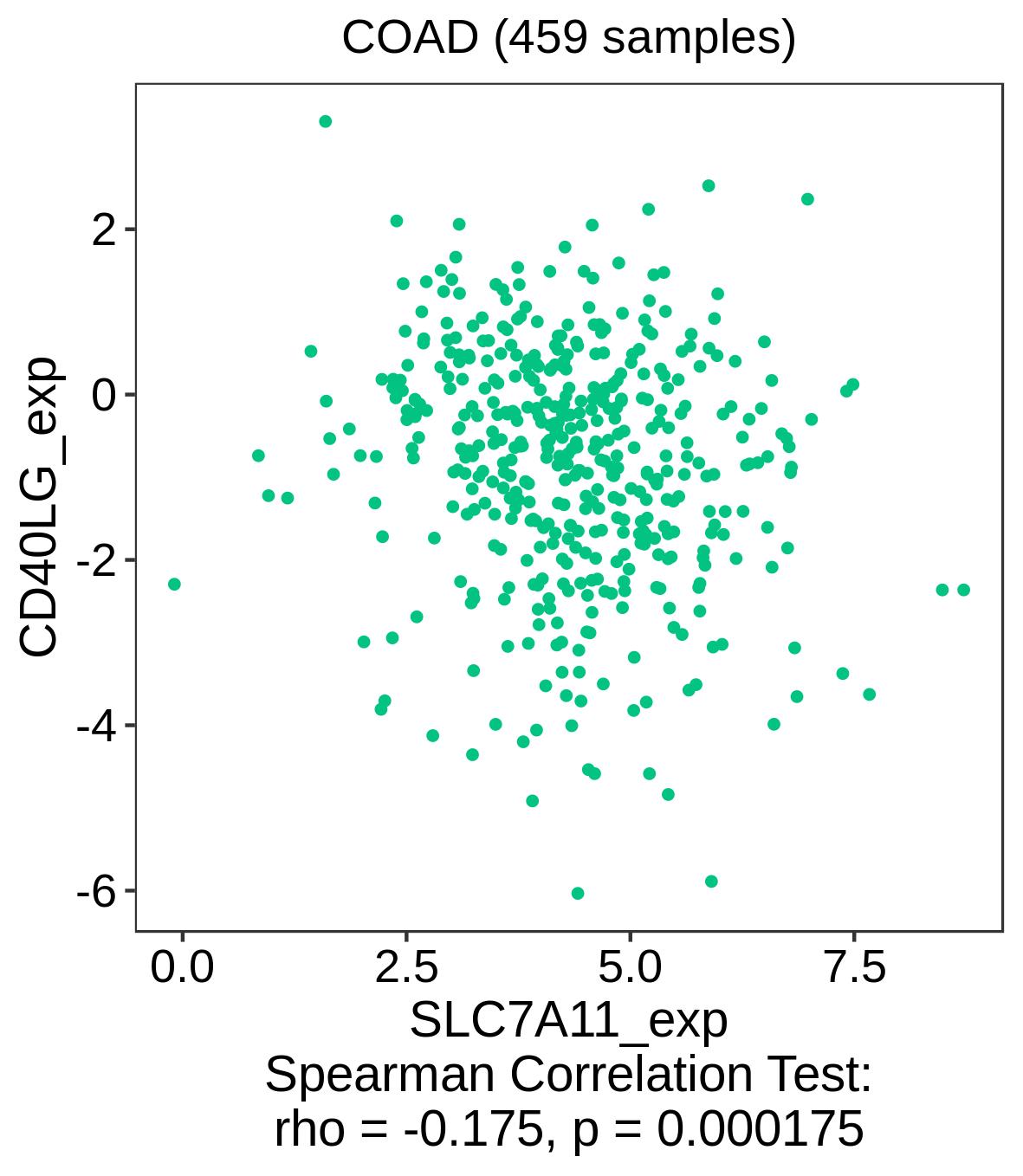

Supplement: Supplementary file 1 [file DataSheet1.ZIP › Raw data/original data/Figure 6 Immune Characteristics/Figure. 6D immunostimulators/SLC7A11_exp_COAD_Immunostimulator_CD40LG.jpg]

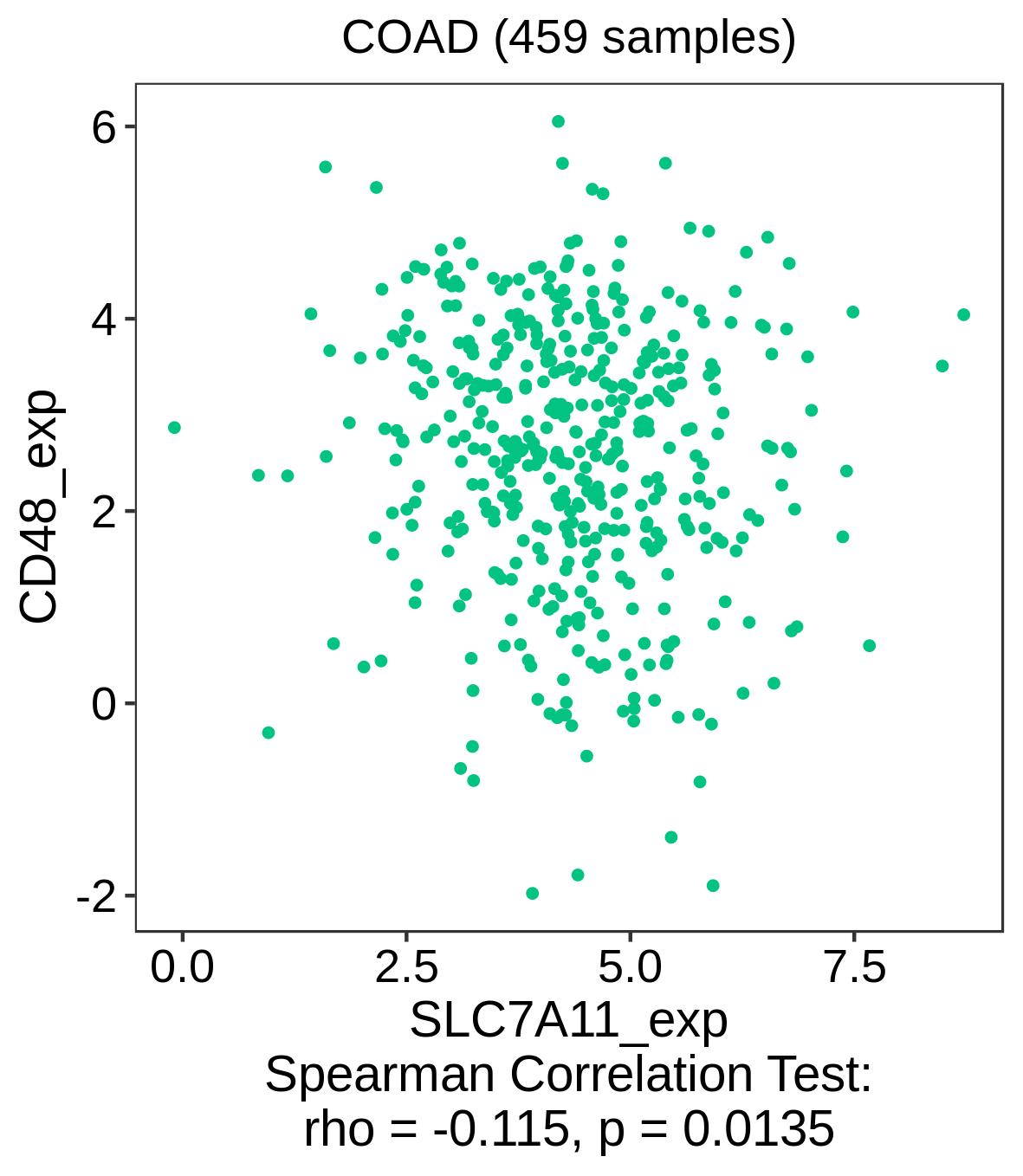

Supplement: Supplementary file 1 [file DataSheet1.ZIP › Raw data/original data/Figure 6 Immune Characteristics/Figure. 6D immunostimulators/SLC7A11_exp_COAD_Immunostimulator_CD48.jpg]

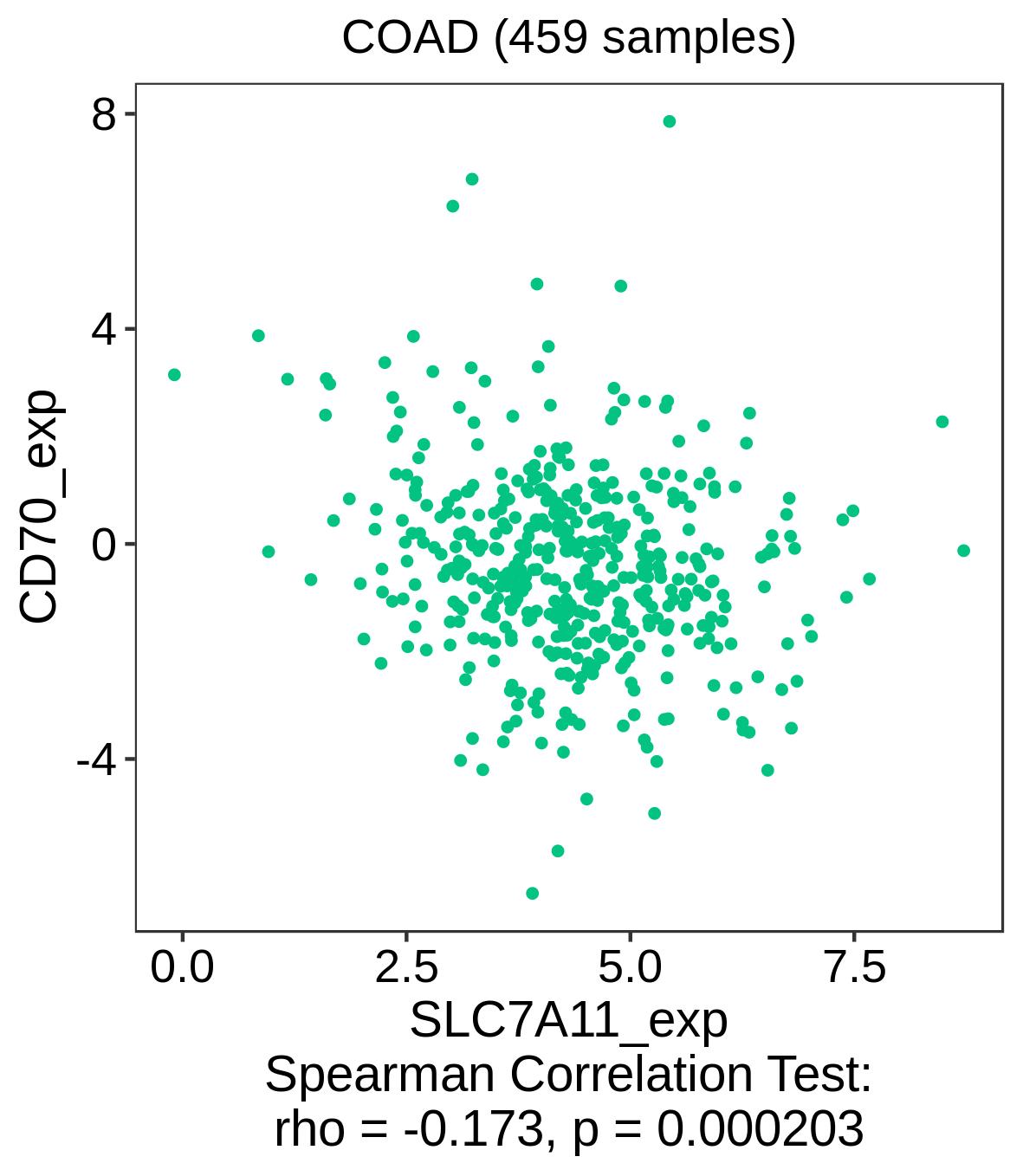

Supplement: Supplementary file 1 [file DataSheet1.ZIP › Raw data/original data/Figure 6 Immune Characteristics/Figure. 6D immunostimulators/SLC7A11_exp_COAD_Immunostimulator_CD70.jpg]

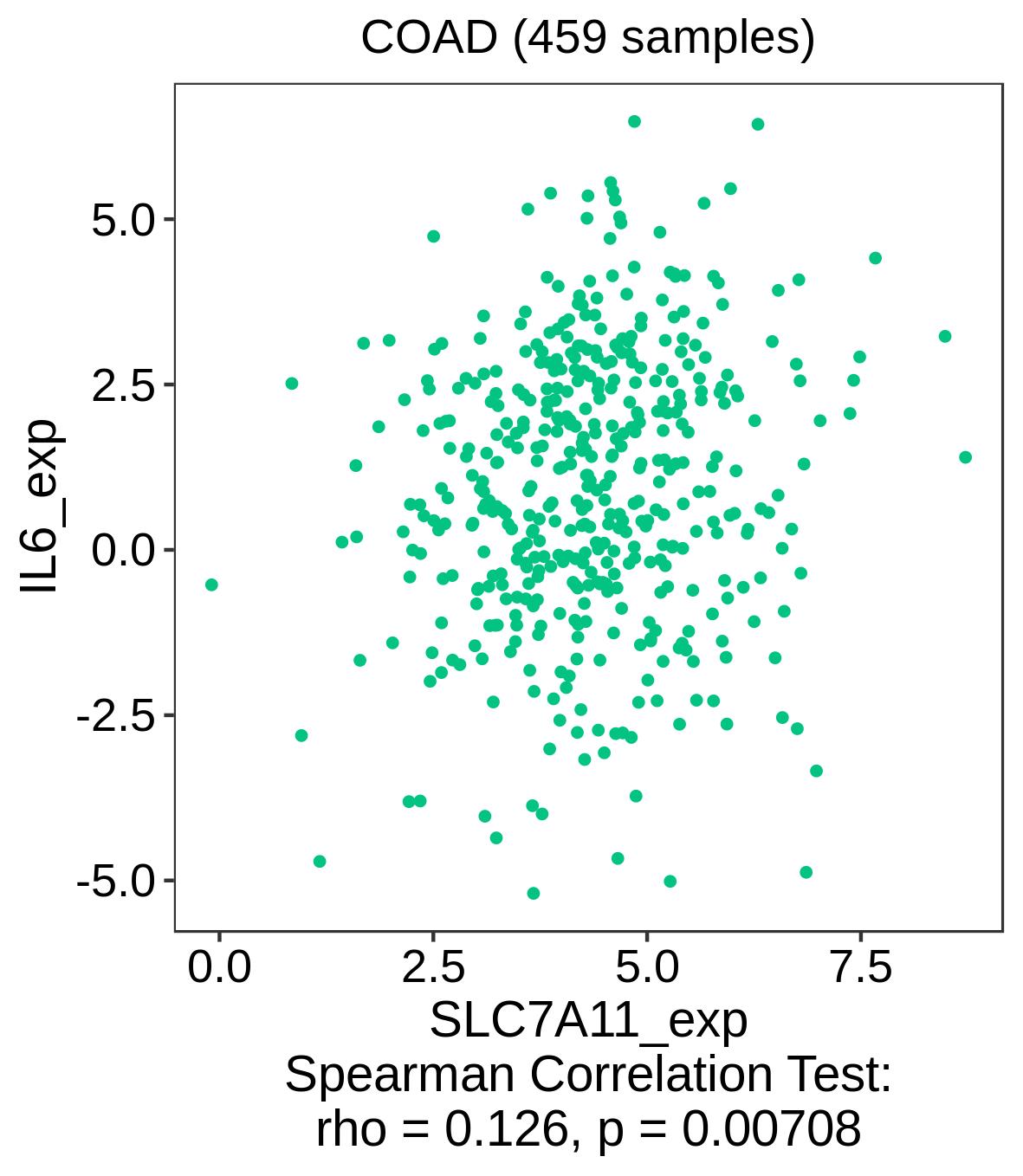

Supplement: Supplementary file 1 [file DataSheet1.ZIP › Raw data/original data/Figure 6 Immune Characteristics/Figure. 6D immunostimulators/SLC7A11_exp_COAD_Immunostimulator_IL6.jpg]

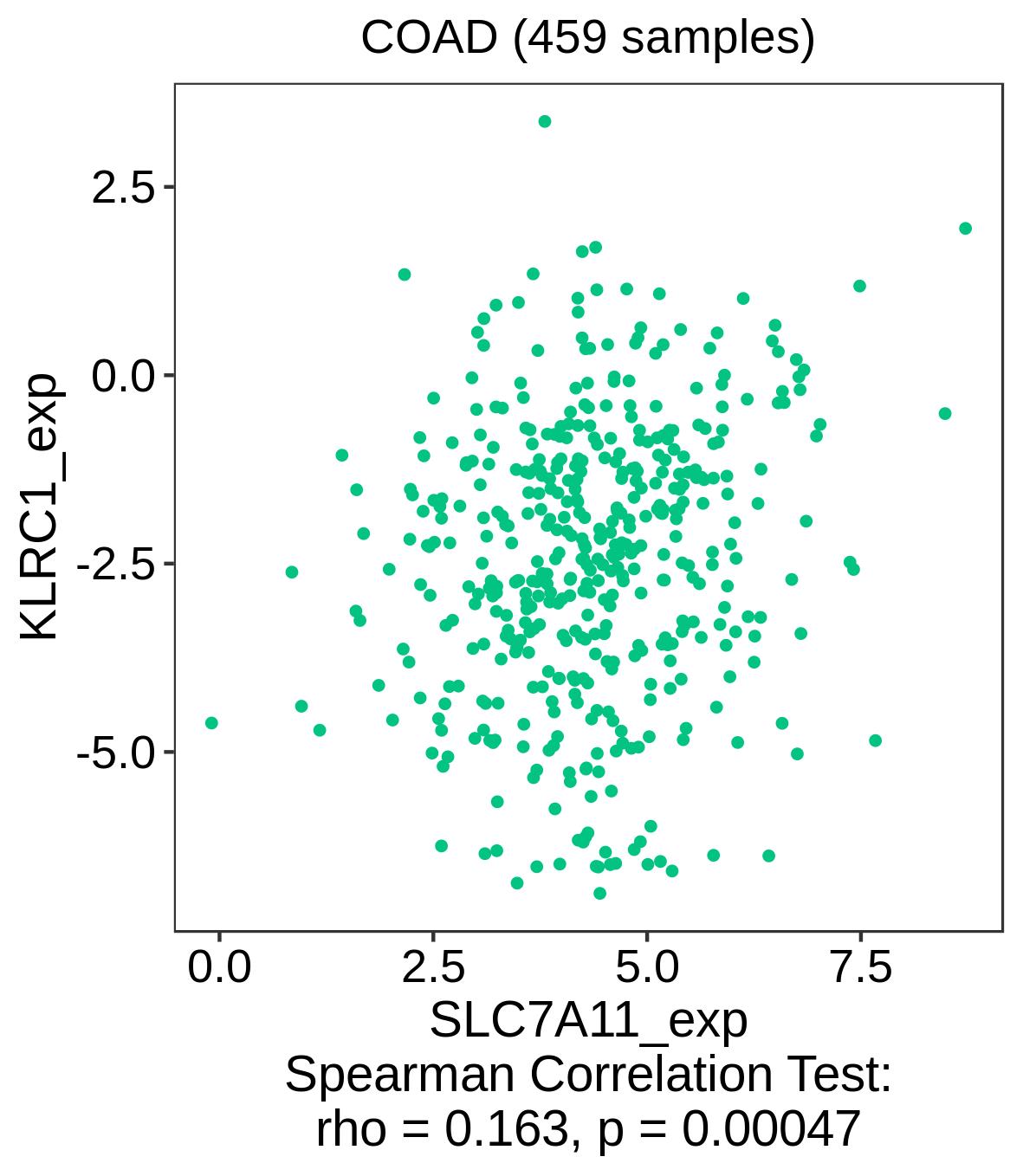

Supplement: Supplementary file 1 [file DataSheet1.ZIP › Raw data/original data/Figure 6 Immune Characteristics/Figure. 6D immunostimulators/SLC7A11_exp_COAD_Immunostimulator_KLRC1.jpg]

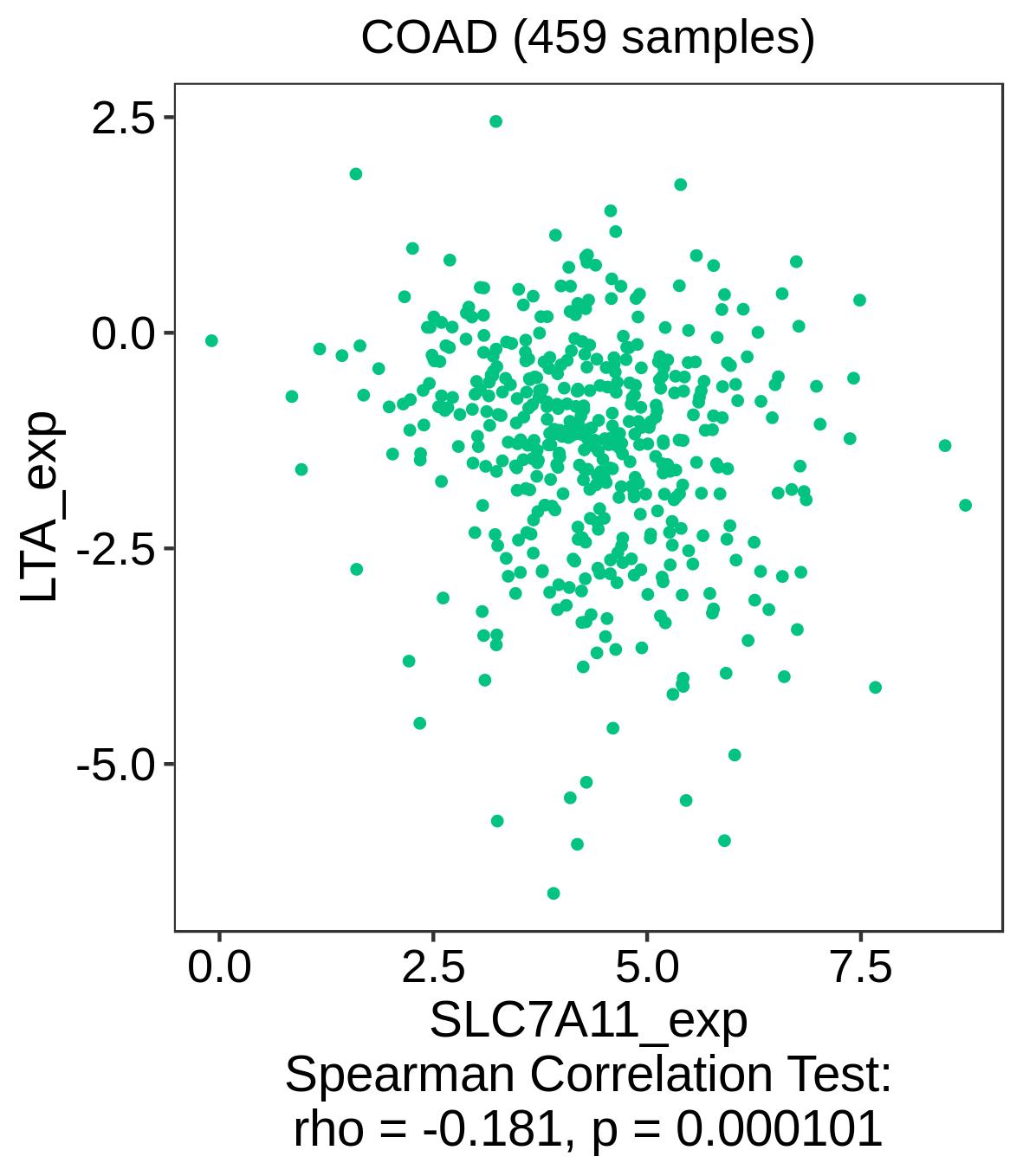

Supplement: Supplementary file 1 [file DataSheet1.ZIP › Raw data/original data/Figure 6 Immune Characteristics/Figure. 6D immunostimulators/SLC7A11_exp_COAD_Immunostimulator_LTA.jpg]

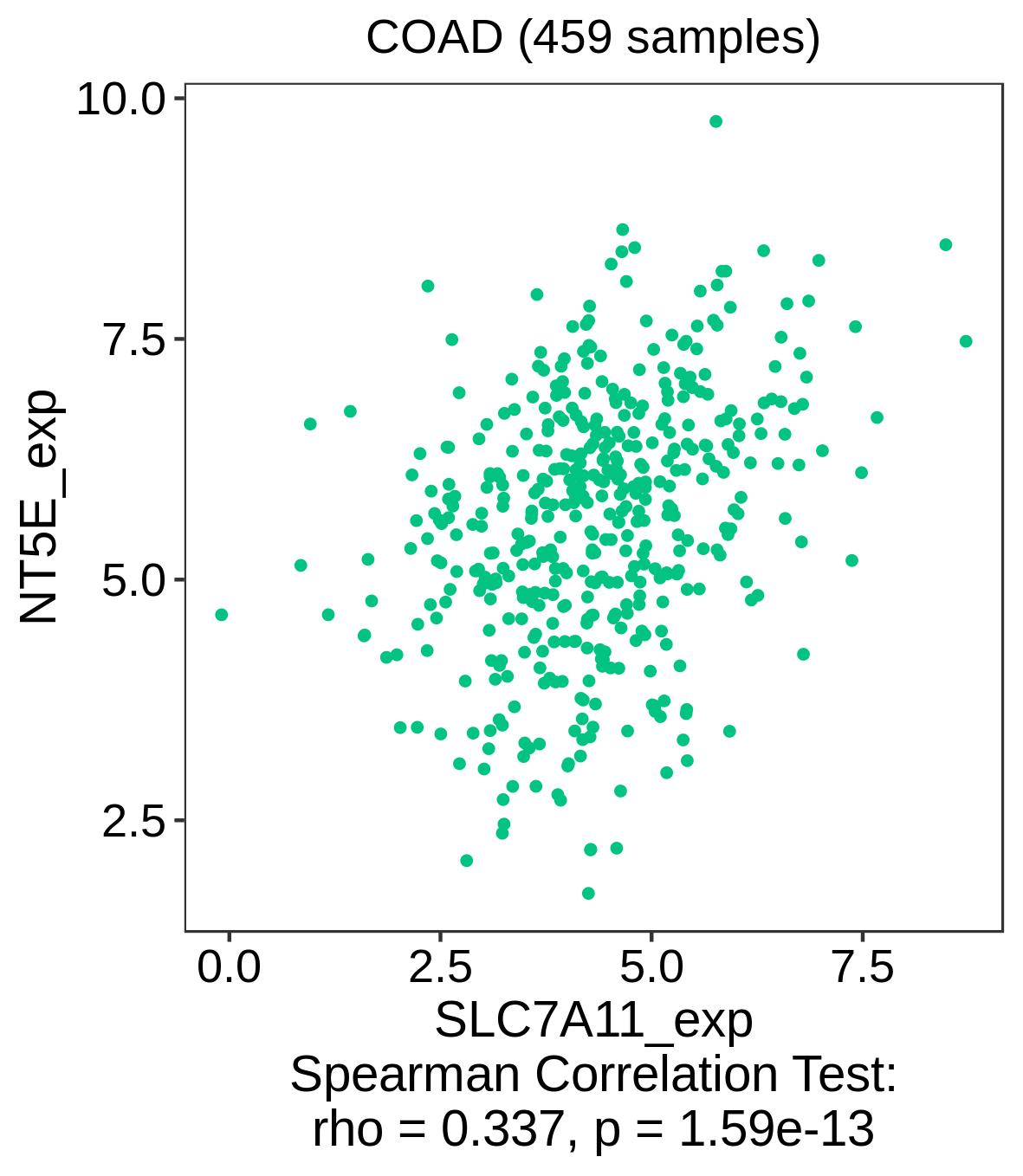

Supplement: Supplementary file 1 [file DataSheet1.ZIP › Raw data/original data/Figure 6 Immune Characteristics/Figure. 6D immunostimulators/SLC7A11_exp_COAD_Immunostimulator_NT5E.jpg]

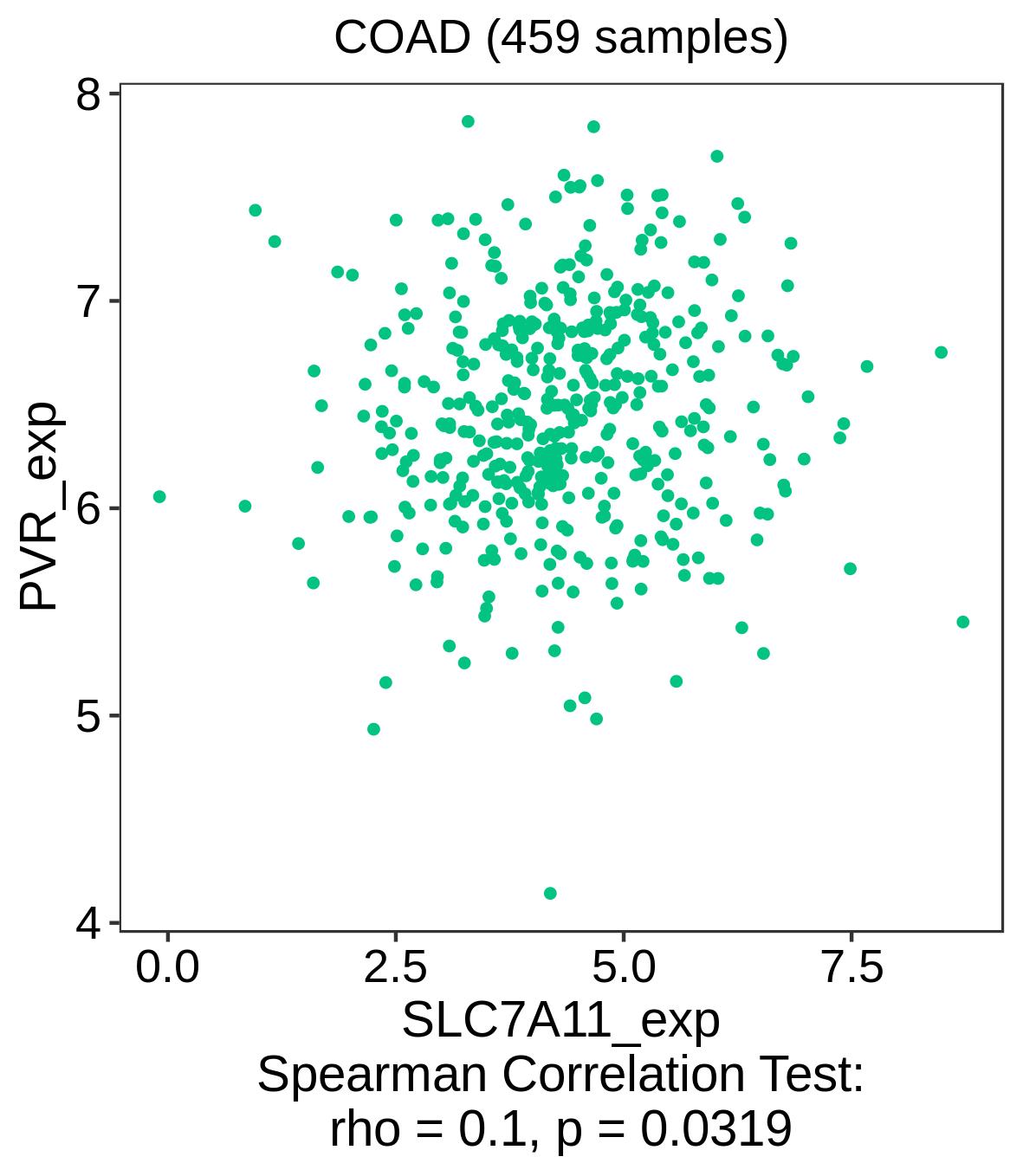

Supplement: Supplementary file 1 [file DataSheet1.ZIP › Raw data/original data/Figure 6 Immune Characteristics/Figure. 6D immunostimulators/SLC7A11_exp_COAD_Immunostimulator_PVR.jpg]

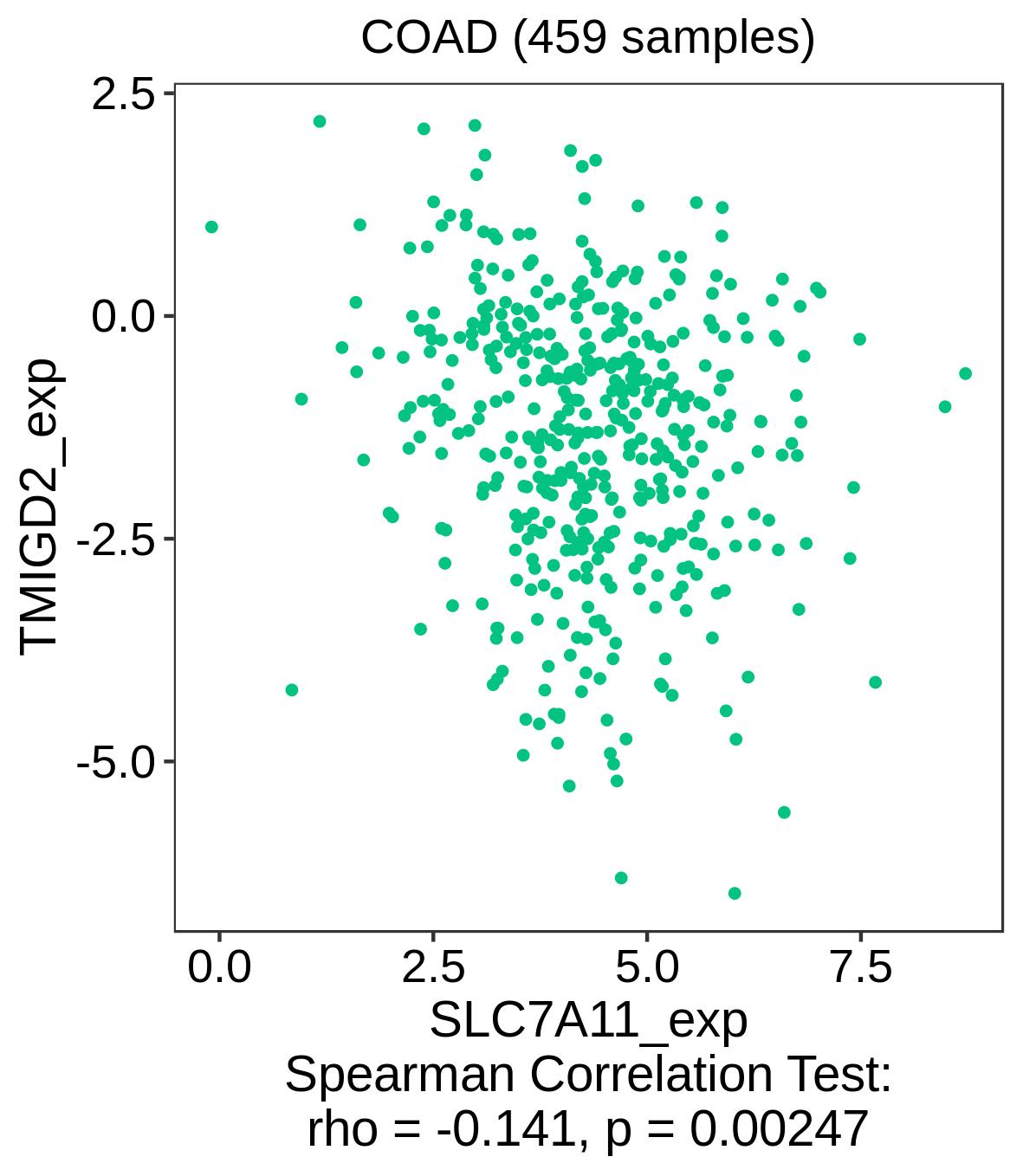

Supplement: Supplementary file 1 [file DataSheet1.ZIP › Raw data/original data/Figure 6 Immune Characteristics/Figure. 6D immunostimulators/SLC7A11_exp_COAD_Immunostimulator_TMIGD2.jpg]

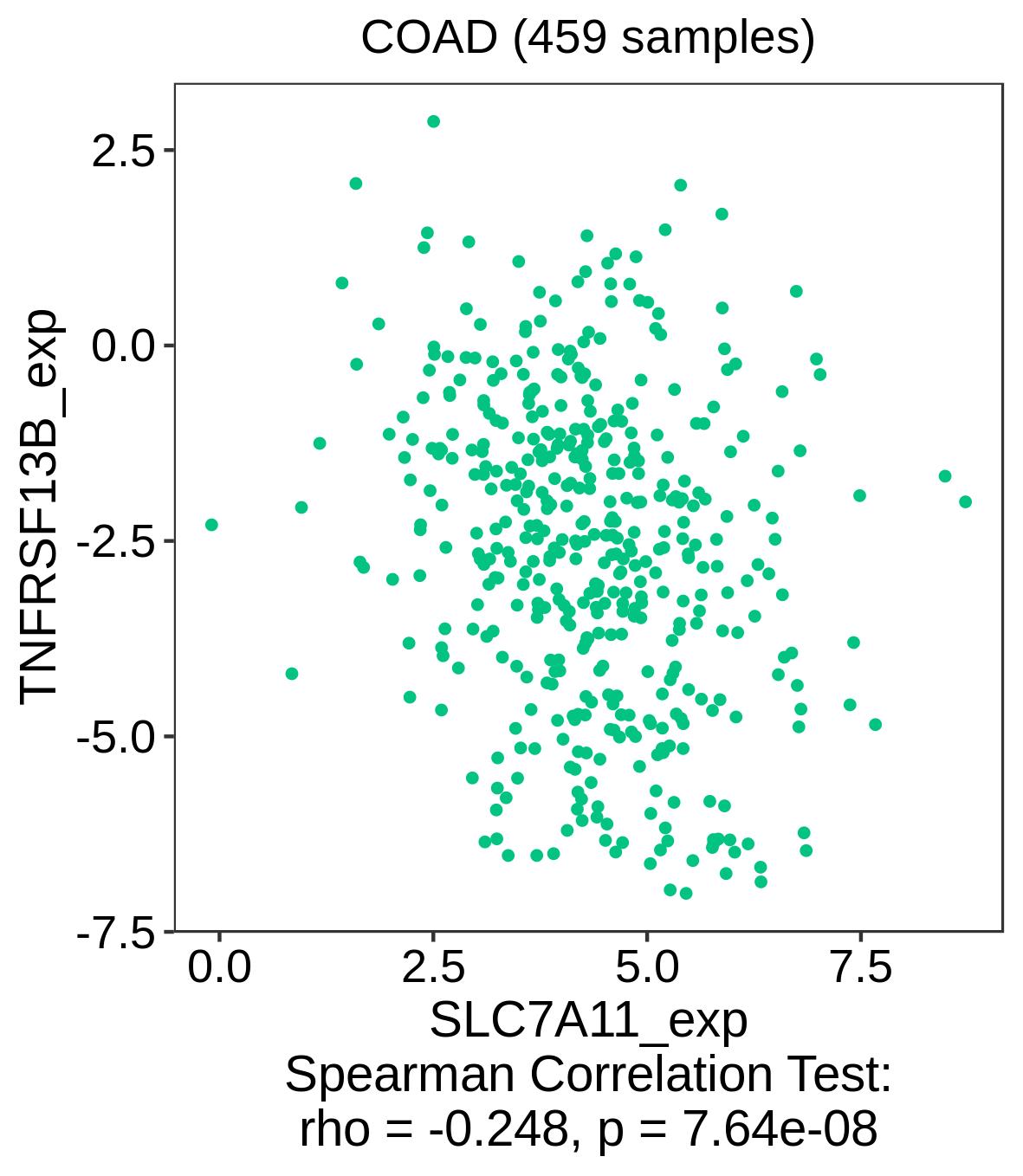

Supplement: Supplementary file 1 [file DataSheet1.ZIP › Raw data/original data/Figure 6 Immune Characteristics/Figure. 6D immunostimulators/SLC7A11_exp_COAD_Immunostimulator_TNFRSF13B.jpg]

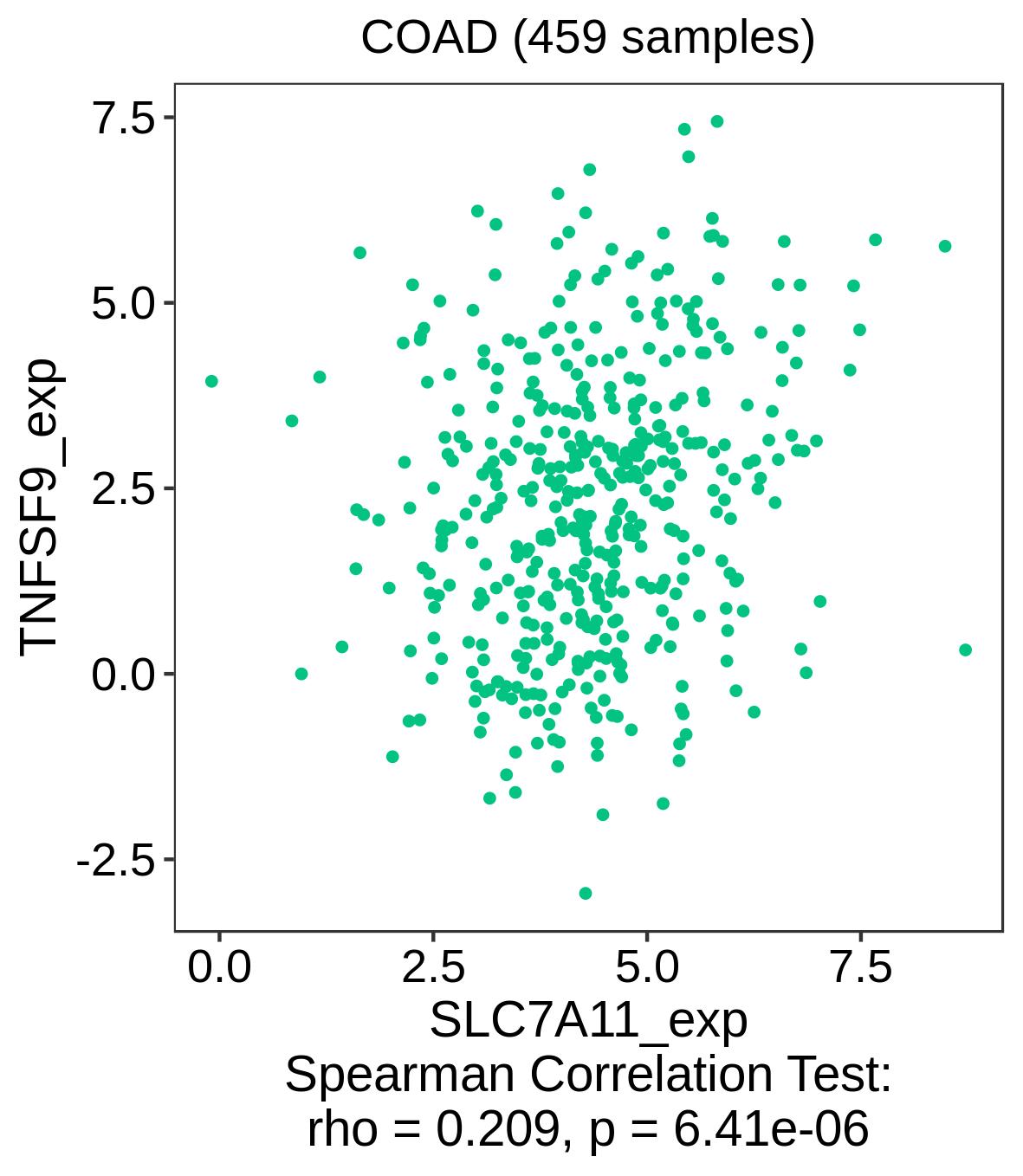

Supplement: Supplementary file 1 [file DataSheet1.ZIP › Raw data/original data/Figure 6 Immune Characteristics/Figure. 6D immunostimulators/SLC7A11_exp_COAD_Immunostimulator_TNFSF9.jpg]

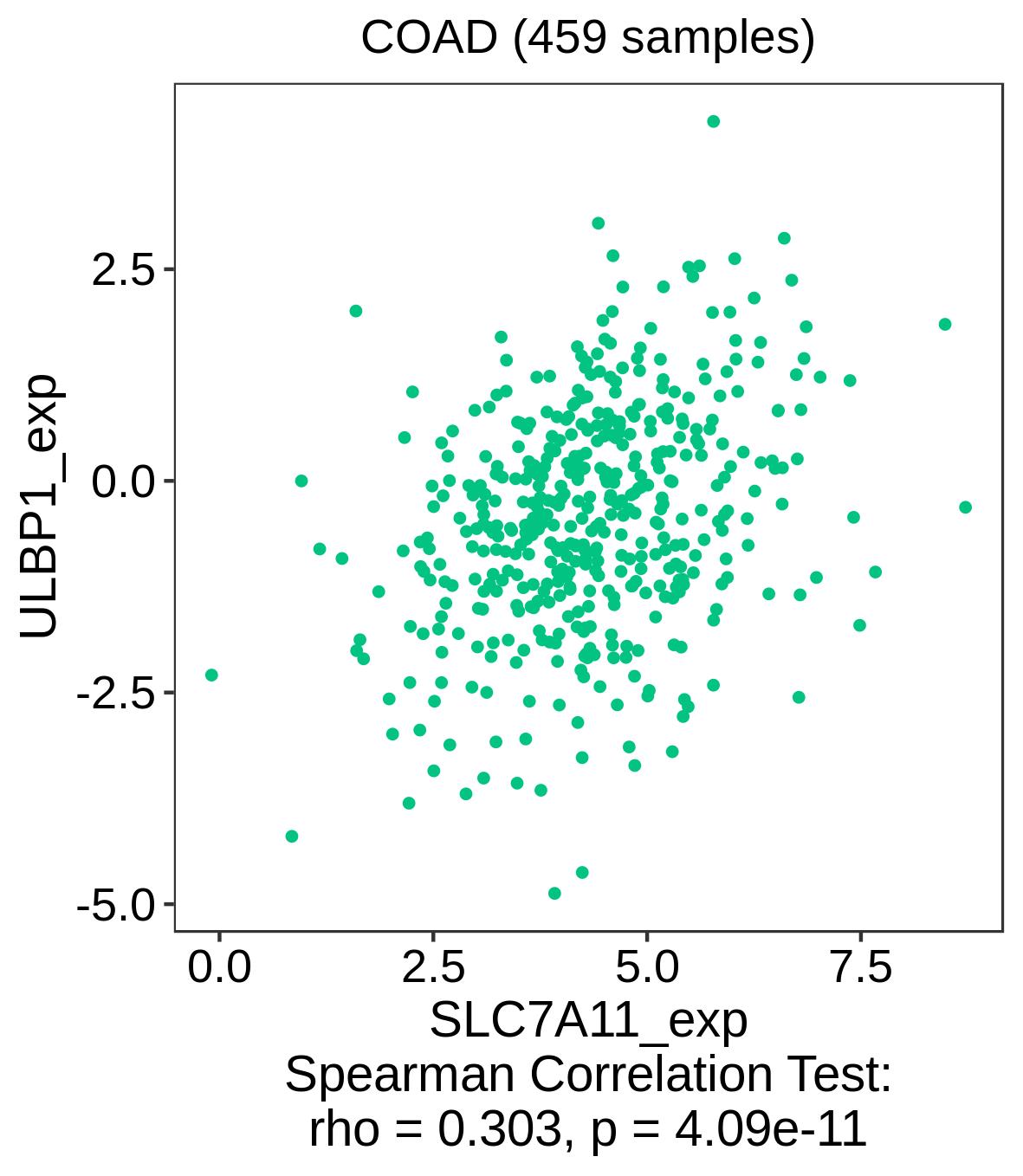

Supplement: Supplementary file 1 [file DataSheet1.ZIP › Raw data/original data/Figure 6 Immune Characteristics/Figure. 6D immunostimulators/SLC7A11_exp_COAD_Immunostimulator_ULBP1.jpg]

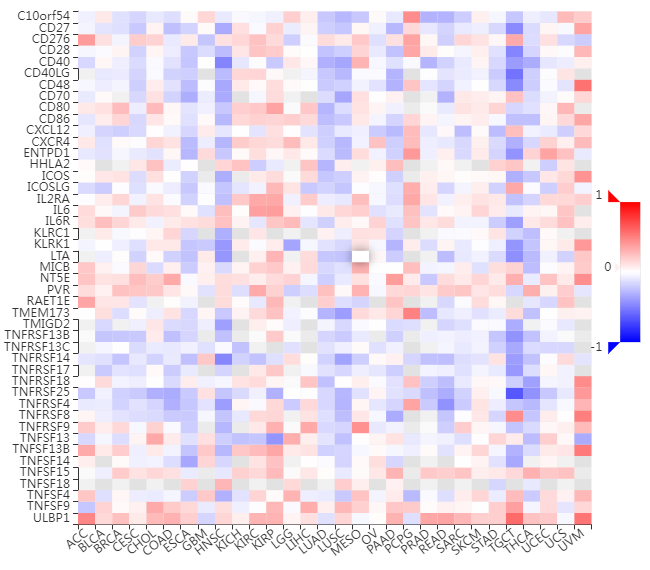

Supplement: Supplementary file 1 [file DataSheet1.ZIP › Raw data/original data/Figure 6 Immune Characteristics/Figure. 6D immunostimulators/╧┬╘╪.png]
