# Supplementary material for: SLC7A11, a Potential Therapeutic Target Through Induced Ferroptosis in Colon Adenocarcinoma
Source: Front Mol Biosci. 2022 Apr 20;9:889688. doi: 10.3389/fmolb.2022.889688 (PMC9065265; doi:10.3389/fmolb.2022.889688)
Supplement: Supplementary file 2 [file DataSheet2.docx]

Supplementary Material


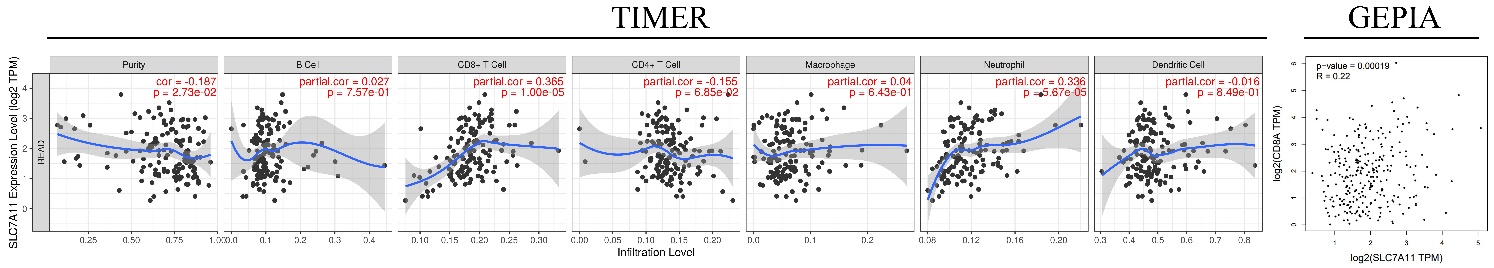


**Supplementary Figure 1.** SLC7A11 affects the infiltration of immune cells in rectal cancer. The TIMER database showed that the expression level of SLC7A11 had obviously positive correlation with infiltrating levels of CD8^+^ T cells (*r* = 0.368, *P* = 1.00e-05) and neutrophils (*r* = 0.336, *P* = 5.67e-05), but it was negatively correlated with the tumor purity (*r* = -0.187, *P* = 2.73e-02) in rectal cancer. The GEPIA database showed that the expression level of SLC7A11 had obviously positive correlation with CD8A ((*r* = 0.22, *P* = 0.00019), a marker of CD8^+^ T cells


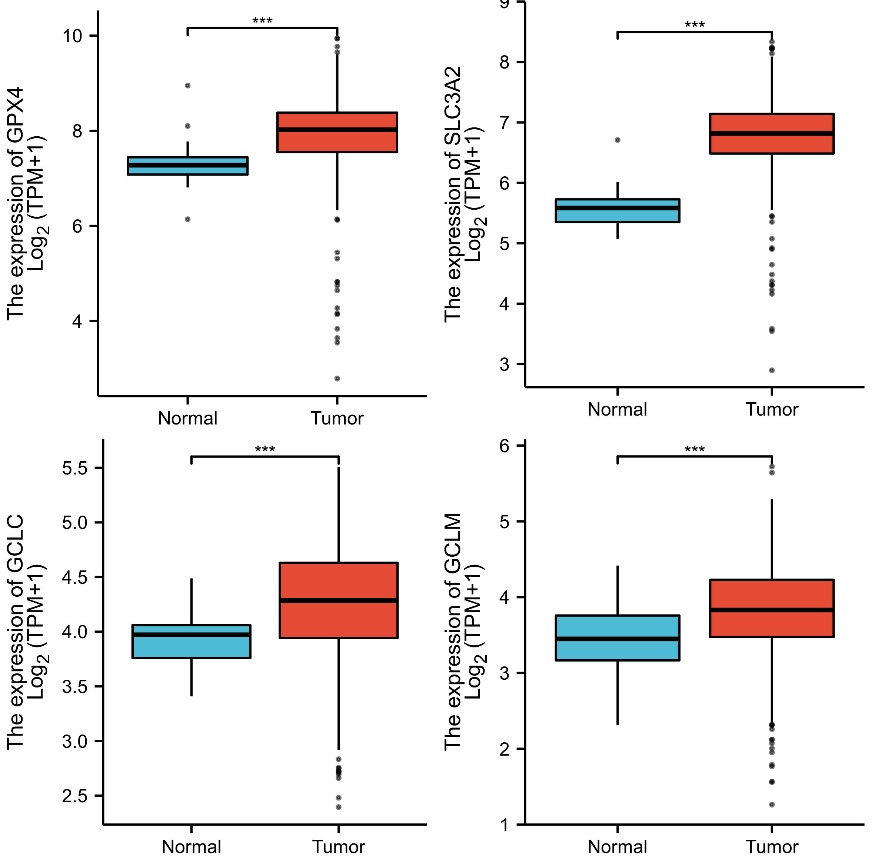


**Supplementary Figure 2.** Expression of GPX4, SLC3A2, GCLC, and GCLM in COAD compared with normal tissues in TCGA database. GPX4, SLC3A2, GCLC, and GCLM were highly expressed in COAD; n (normal) = 41, n (tumor) = 480


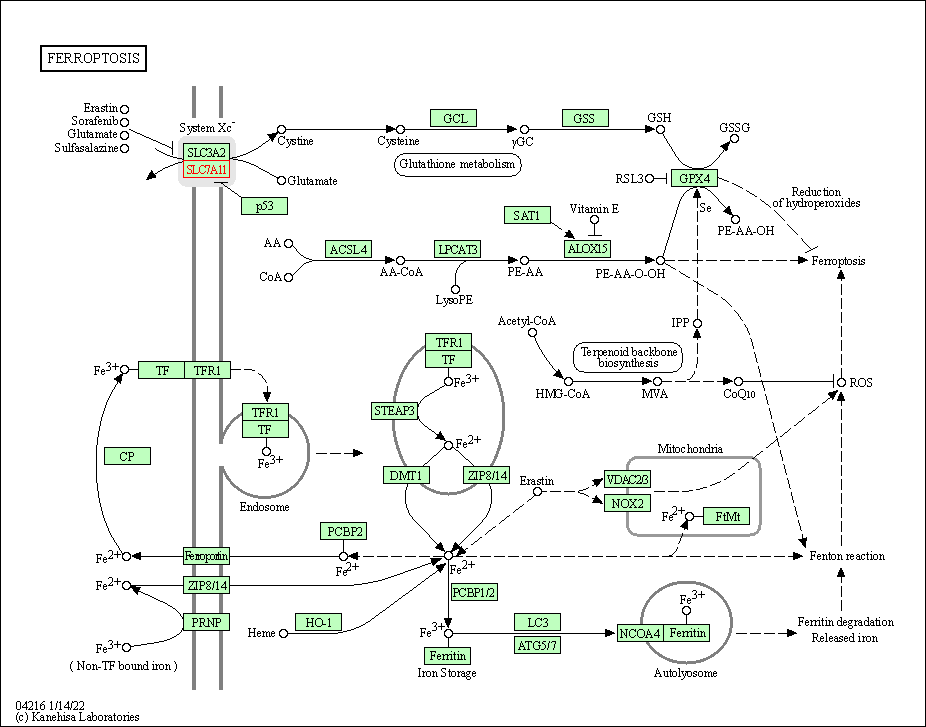


**Supplementary Figure 3.** Ferroptosis signaling pathway (KEGG database)
